# Supplementary material for: Dicationic Bis-Pyridinium Hydrazone-Based Amphiphiles Encompassing Fluorinated Counteranions: Synthesis, Characterization, TGA-DSC, and DFT Investigations
Source: Molecules. 2022 Apr 12;27(8):2492. doi: 10.3390/molecules27082492 (PMC9030580; doi:10.3390/molecules27082492)
Supplement: Supplementary file 1 [file molecules-27-02492-s001.zip › molecules-1619504-supplementary.pdf]

Supplementary Materials

Dicationic Bis-Pyridinium Hydrazone-Based Amphiphiles Encompassing Fluorinated Counteranions: Synthesis, Characterization, TGA-DSC, and DFT Investigations

Ateyatallah Aljuhani [1], Nadjet Rezki [1,*], Salsabeel Al-Sodies [1], Mouslim Messali [1], Gamal M.S. ElShafei [2], Mohamed Hagar [3] and Mohamed R. Aouad [1,*]

1. Chemicals, reagents and instruments used*:*

All of the chemicals and solvents employed were of the greatest purity and analytical reagent grade, and none of them were refined any further. An uncorrected Stuart Scientific SMP1 apparatus was used to measure melting points (Stuart, Red Hill, UK). The NMR measurements were performed on a Bruker spectrometer (400 Brucker, Fällanden, Switzerland) with Tetramethylsilane (TMS) as an internal standard (0.00 ppm). For high resolution mass spectroscopy, an LCMS/MS impact II was used (HRMS). A TA Instruments SDT-Q600 was used to perform thermal gravimetric analysis (TGA). The DSC measurements of the examined substances were conducted using a Differential Scanning Calorimeter (DSC; MA, USA). The melting temperature, enthalpy, and lead were used to calibrate the DSC. DSC experiments were carried out with small samples (2-3 mg) placed in aluminum pans. All measurements were taken at a heating rate of 10 °C/min in a nitrogen gas inert atmosphere (30 ml/min), and all transitions were recorded from the second heating scan.

2. Spectroscopic characterization of the synthesized compounds 9-36

Characterization of 1-octyl-4-((2-(1-octylpyridin-1-ium-4-carbonyl)hydrazono)methyl) pyridin-1-ium iodide (9)

It was obtained as yellow crystals in 88% yield; mp: 211-212 ºC. FT-IR (KBr), cm^{-1}: $\overline{\nu}$ = 1549 (C=C), 1638 (C=N), 1686 (C=O), 2848-2914 (Al-H). ^{1}H NMR (400 MHz, DMSO-d_6): δ_H = 0.85 (t, 6H, *J* = 8 Hz, 2×C**H**$_3$), 1.25-1.29 (m, 20H, 10×C**H**$_2$), 1.96 (bs, 4H, 2×NCH$_2$C**H**$_2$), 4.60-4.72 (m, 4H, 2×NC**H**$_2$), 8.20 (d, 0.5H, *J* = 8 Hz, Ar-**H**), 8.30 (s, 0.25H, **H**-C=N), 8.43 (d, 0.5H, *J* = 8 Hz, Ar-**H**), 8.46 (d, 1.5H, *J* = 8 Hz, Ar-**H**), 8.56 (d, 1.5H, *J* = 4 Hz, Ar-**H**), 8.64 (s, 0.75H, **H**-C=N), 9.04 (d, 0.5H, *J* = 8 Hz, Ar-**H**), 9.16 (d, 1.5H, *J* = 8 Hz, Ar-**H**), 9.28 (d, 0.5H, *J* = 4 Hz, Ar-**H**), 9.38 (d, 1.5H, *J* = 4 Hz, Ar-**H**), 13.20 (s, 1H, CON**H**). ^{13}C NMR (100MHz, DMSO-d_6): δ_C = 14.43 (2×**C**H$_3$), 22.52, 25.87, 28.82, 28.92, 31.15, 31.60 (12×**C**H$_2$), 60.96, 61.62 (2×N**C**H$_2$), 125.26, 125.42, 126.91, 127.74, 144.95, 145.37, 145.66, 146.33, 146.96, 149.14 (Ar-**C**), 160.32, 160.35 (**C**=N, **C**=O). HRMS (ESI) m/z = 706.1604 [M^+].

Characterization of 1-nonyl-4-((2-(1-nonylpyridin-1-ium-4-carbonyl)hydrazono) methyl)pyridin-1-ium iodide (10)

It was obtained as yellow crystals in 85% yield; mp: 215-216 ºC. FT-IR (KBr), cm^{-1}: $\bar{\nu}$ = 1553 (C=C), 1638 (C=N), 1694 (C=O), 2856-2927 (Al-H). ^1H NMR (400 MHz, DMSO-d_6): δ_H = 0.85 (t, 6H, *J* = 8 Hz, 2×C**H**$_3$), 1.23-1.29 (m, 24H, 12×C**H**$_2$), 1.95 (bs, 4H, 2×NCH$_2$C**H**$_2$), 4.59-4.70 (m, 4H, 2×NC**H**$_2$), 8.17 (d, 0.5H, *J* = 4 Hz, Ar-**H**), 8.29 (s, 0.25H, **H**-C=N), 8.43 (d, 0.5H, *J* = 8 Hz, Ar-**H**), 8.45 (d, 1.5H, *J* = 4 Hz, Ar-**H**), 8.56 (d, 1.5H, *J* = 8 Hz, Ar-**H**), 8.63 (s, 0.75H, **H**-C=N), 9.02 (d, 0.5H, *J* = 8 Hz, Ar-**H**), 9.14 (d, 1.5H, *J* = 8 Hz, Ar-**H**), 9.26 (d, 0.5H, *J* = 4 Hz, Ar-**H**), 9.36 (d, 1.5H, *J* = 8 Hz, Ar-**H**), 13.18 (s, 1H, CON**H**). ^{13}C NMR (100 MHz, DMSO-d_6): δ_C = 14.44 (2×**C**H$_3$), 22.57, 25.88, 25.91, 28.87, 29.18, 29.27, 29.39, 29.48, 29.53, 31.16, 31.76 (14×**C**H$_2$), 61.67, 61.72 (2×N**C**H$_2$), 125.26, 125.42, 126.91, 127.74, 144.95, 145.37, 145.66, 146.33, 146.96, 149.14 (Ar-**C**), 160.32, 160.35 (**C**=N, **C**=O). HRMS (ESI) m/z = 734.1917 [M^+].

Characterization of 1-decyl-4-((2-(1-decylpyridin-1-ium-4-carbonyl)hydrazono)methyl) pyridin-1-ium iodide (11)

It was obtained as yellow crystals in 86% yield; mp: 217-218 ºC. FT-IR (KBr), cm^{-1}: $\bar{\nu}$ = 1549 (C=C), 1639 (C=N), 1691 (C=O), 2849-2918 (Al-H). ^1H NMR (400 MHz, DMSO-d_6): δ_H = 0.84 (t, 6H, *J* = 4 Hz, 2×C**H**$_3$), 1.24-1.29 (m, 28H, 14×C**H**$_2$), 1.95 (bs, 4H, 2×NCH$_2$C**H**$_2$), 4.59-4.71 (m, 4H, 2×NC**H**$_2$), 8.19 (d, 0.5H, *J* = 4 Hz, Ar-**H**), 8.30 (s, 0.25H, **H**-C=N), 8.43 (d, 0.5H, *J* = 8 Hz, Ar-**H**), 8.46 (d, 1.5H, *J* = 4 Hz, Ar-**H**), 8.56 (d, 1.5H, *J* = 4 Hz, Ar-**H**), 8.63 (s, 0.75H, **H**-C=N), 9.02 (d, 0.5H, *J* = 4 Hz, Ar-**H**), 9.15 (d, 1.5H, *J* = 4 Hz, Ar-**H**), 9.28 (d, 0.5H, *J* = 4 Hz, Ar-**H**), 9.37 (d, 1.5H, *J* = 4 Hz, Ar-**H**), 13.20 (s, 1H, CON**H**). ^{13}C NMR (100 MHz, DMSO-d_6): δ_C = 14.45 (2×**C**H$_3$), 22.57, 25.87, 25.91, 28.86, 29.13, 29.27, 29.35, 29.48, 31.15, 31.75 (16×**C**H$_2$), 60.95, 61.62 (2×N**C**H$_2$), 125.26, 125.42, 126.91, 127.74, 144.95, 145.37, 145.66, 146.33, 146.96, 149.14 (Ar-**C**), 160.25, 160.33 (**C**=N, **C**=O). HRMS (ESI) m/z = 762.2230 [M^+].

Characterization of 1-dodecyl-4-((2-(1-dodecylpyridin-1-ium-4-carbonyl)hydrazono) methyl)pyridin-1-ium iodide (12)

It was obtained as yellow crystals in 88% yield; mp: 209-210 ºC. FT-IR (KBr), cm^{-1}: $\bar{\nu}$ = 1547 (C=C), 1640 (C=N), 1689 (C=O), 2842-2917 (Al-H). ^1H NMR (400 MHz,

DMSO-d_6): δ_H = 0.84 (t, 6H, *J* = 4 Hz, 2×C**H**$_3$), 1.23-1.29 (m, 36H, 18×C**H**$_2$), 1.95 (bs, 4H, 2×NCH$_2$C**H**$_2$), 4.59-4.71 (m, 4H, 2×NC**H**$_2$), 8.18 (d, 0.5H, *J* = 4 Hz, Ar-**H**), 8.30 (s, 0.25H, **H**-C=N), 8.43 (d, 0.5H, *J* = 8 Hz, Ar-**H**), 8.45 (d, 1.5H, *J* = 4 Hz, Ar-**H**), 8.56 (d, 1.5H, *J* = 4 Hz, Ar-**H**), 8.63 (s, 0.75H, **H**-C=N), 9.03 (d, 0.5H, *J* = 8 Hz, Ar-**H**), 9.15 (d, 1.5H, *J* = 8 Hz, Ar-**H**), 9.28 (d, 0.5H, *J* = 4 Hz, Ar-**H**), 9.37 (d, 1.5H, *J* = 4 Hz, Ar-**H**), 13.20 (s, 1H, CON**H**). ^{13}C NMR (100 MHz, DMSO-d_6): δ_C = 14.44 (2×**C**H$_3$), 22.57, 25.86, 29.19, 29.27, 29.38, 29.39, 29.48, 31.15, 31.76 (20×**C**H$_2$), 60.95, 61.62 (2×N**C**H$_2$), 125.26, 125.42, 126.91, 127.74, 144.93, 145.37, 145.66, 146.35, 146.96, 149.17 (Ar-**C**), 160.25, 160.33 (**C**=N, **C**=O). HRMS (ESI) m/z = 818.2856 [M$^+$].

Characterization of 1-tetradecyl-4-((2-(1-tetradecylpyridin-1-ium-4-carbonyl) hydrazono)methyl)pyridin-1-ium iodide (13)

It was obtained as yellow crystals in 87% yield; mp: 194-195 ºC. FT-IR (KBr), cm^{-1}: $\bar{\nu}$ = 1552 (C=C), 1642 (C=N), 1695 (C=O), 2847-2919 (Al-H). ^{1}H NMR (400 MHz, DMSO-d_6): δ_H = 0.84 (t, 6H, *J* = 4 Hz, 2×C**H**$_3$), 1.24-1.29 (m, 44H, 22×C**H**$_2$), 1.96 (bs, 4H, 2×NCH$_2$C**H**$_2$), 4.60-4.72 (m, 4H, 2×NC**H**$_2$), 8.20 (d, 0.5H, *J* = 4 Hz, Ar-**H**), 8.30 (s, 0.25H, **H**-C=N), 8.43 (d, 0.5H, *J* = 8 Hz, Ar-**H**), 8.46 (d, 1.5H, *J* = 8 Hz, Ar-**H**), 8.56 (d, 1.5H, *J* = 4 Hz, Ar-**H**), 8.64 (s, 0.75H, **H**-C=N), 9.04 (d, 0.5H, *J* = 4 Hz, Ar-**H**), 9.16 (d, 1.5H, *J* = 4 Hz, Ar-**H**), 9.29 (d, 0.5H, *J* = 4 Hz, Ar-**H**), 9.39 (d, 1.5H, *J* = 8 Hz, Ar-**H**), 13.19 (s, 1H, CON**H**). ^{13}C NMR (100 MHz, DMSO-d_6): δ_C = 14.44 (2×**C**H$_3$), 22.56, 25.86, 28.86, 29.09, 29.22, 29.38, 29.48, 31.15, 31.69 (24×**C**H$_2$), 60.94, 61.61 (2×N**C**H$_2$), 125.26, 125.41, 126.90, 127.72, 144.93, 145.34, 145.65, 146.35, 146.98, 149.14 (Ar-**C**), 160.33, 160.40 (**C**=N, **C**=O). HRMS (ESI) m/z = 874.3482 [M$^+$].

Characterization of 1-hexadecyl-4-((2-(1-hexadecylpyridin-1-ium-4-carbonyl) hydrazono)methyl)pyridin-1-ium iodide (14)

It was obtained as yellow crystals in 90% yield; mp: 190-191 ºC. FT-IR (KBr), cm^{-1}: $\bar{\nu}$ = 1546 (C=C), 1639 (C=N), 1688 (C=O), 2846-2918 (Al-H). ^{1}H NMR (400 MHz, DMSO-d_6): δ_H = 0.84 (t, 6H, *J* = 8 Hz, 2×C**H**$_3$), 1.22-1.28 (m, 52H, 26×C**H**$_2$), 1.95 (bs, 4H, 2×NCH$_2$C**H**$_2$), 4.57-4.69 (m, 4H, 2×NC**H**$_2$), 8.15 (d, 0.5H, *J* = 4 Hz, Ar-**H**), 8.28 (s, 0.25H, **H**-C=N), 8.40 (d, 0.5H, *J* = 8 Hz, Ar-**H**), 8.44 (d, 1.5H, *J* = 8 Hz, Ar-**H**), 8.55 (d,

1.5H, J = 8 Hz, Ar-**H**), 8.61 (s, 0.75H, **H**-C=N), 8.99 (d, 0.5H, J = 4 Hz, Ar-**H**), 9.12 (d, 1.5H, J = 4 Hz, Ar-**H**), 9.24 (d, 0.5H, J = 4 Hz, Ar-**H**), 9.33 (d, 1.5H, J = 8 Hz, Ar-**H**), 13.19 (s, 1H, CON**H**). ^{13}C NMR (100 MHz, DMSO-d_6): δ_C = 14.43 (2×**C**H$_3$), 22.55, 25.85, 29.16, 29.25, 29.38, 29.46, 29.50, 31.15, 31.75 (28×**C**H$_2$), 60.94, 61.61 (2×N**C**H$_2$), 125.26, 125.41, 126.90, 127.72, 144.93, 145.34, 145.65, 146.35, 146.98, 149.14 (Ar-**C**), 160.35, 161.33 (**C**=N, **C**=O). HRMS (ESI) m/z = 930.4108 [M$^+$].

Characterization of 1-octadecyl-4-((2-(1-octadecylpyridin-1-ium-4-carbonyl) hydrazono)methyl)pyridin-1-ium iodide (15)

It was obtained as yellow crystals in 88% yield; mp: 192-193 ºC. FT-IR (KBr), cm^{-1}: $\bar{\nu}$ = 1545 (C=C), 1643 (C=N), 1691 (C=O), 2845-2920 (Al-H). ^{1}H NMR (400 MHz, DMSO-d_6): δ_H = 0.84 (t, 6H, J = 8 Hz, 2×C**H$_3$**), 1.23-1.29 (m, 60H, 30×C**H$_2$**), 1.94 (bs, 4H, 2×NCH$_2$C**H$_2$**), 4.59-4.69 (m, 4H, 2×NC**H$_2$**), 8.18 (d, 0.5H, J = 8 Hz, Ar-**H**), 8.29 (s, 0.25H, **H**-C=N), 8.43 (d, 0.5H, J = 8 Hz, Ar-**H**), 8.46 (d, 1.5H, J = 8 Hz, Ar-**H**), 8.56 (d, 1.5H, J = 8 Hz, Ar-**H**), 8.63 (s, 0.75H, **H**-C=N), 9.02 (d, 0.5H, J = 8 Hz, Ar-**H**), 9.14 (d, 1.5H, J = 4 Hz, Ar-**H**), 9.28 (d, 0.5H, J = 4 Hz, Ar-**H**), 9.36 (d, 1.5H, J = 4 Hz, Ar-**H**), 13.18 (s, 1H, CON**H**). ^{13}C NMR (100 MHz, DMSO-d_6): δ_C = 14.44 (2×**C**H$_3$), 22.57, 25.86, 28.87, 29.18, 29.27, 29.39, 29.48, 31.16, 31.76 (24×**C**H$_2$), 60.97, 61.65 (2×N**C**H$_2$), 125.26, 125.41, 126.90, 127.72, 144.93, 145.34, 145.65, 146.35, 146.98, 149.14 (Ar-**C**), 160.33, 160.40 (**C**=N, **C**=O). HRMS (ESI) m/z = 986.4734 [M$^+$].

Characterization of 1-octyl-4-((2-(1-octylpyridin-1-ium-4-carbonyl)hydrazono)methyl) pyridin-1-ium hexafluorophosphate (16)

It was obtained as yellow crystals in 89% yield; mp: 200-201 ºC. FT-IR (KBr), cm^{-1}: $\bar{\nu}$ = 1548 (C=C), 1639 (C=N), 1688 (C=O), 2842-2910 (Al-H). ^{1}H NMR (400 MHz, DMSO-d_6): δ_H = 0.85 (t, 6H, J = 8 Hz, 2×C**H$_3$**), 1.25-1.29 (m, 20H, 10×C**H$_2$**), 1.96 (bs, 4H, 2×NCH$_2$C**H$_2$**), 4.60-4.72 (m, 4H, 2×NC**H$_2$**), 8.20 (d, 0.5H, J = 8 Hz, Ar-**H**), 8.30 (s, 0.25H, **H**-C=N), 8.43 (d, 0.5H, J = 8 Hz, Ar-**H**), 8.46 (d, 1.5H, J = 8 Hz, Ar-**H**), 8.56 (d, 1.5H, J = 4 Hz, Ar-**H**), 8.64 (s, 0.75H, **H**-C=N), 9.04 (d, 0.5H, J = 8 Hz, Ar-**H**), 9.16 (d, 1.5H, J = 8 Hz, Ar-**H**), 9.28 (d, 0.5H, J = 4 Hz, Ar-**H**), 9.38 (d, 1.5H, J = 4 Hz, Ar-**H**), 13.20 (s, 1H, CON**H**). ^{13}C NMR (100 MHz, DMSO-d_6): δ_C = 14.43 (2×**C**H$_3$), 22.52,

25.87, 28.82, 28.92, 31.15, 31.60 (12×**C**H_2), 60.96, 61.62 (2×N**C**H_2), 125.26, 125.42, 126.91, 127.74, 144.95, 145.37, 145.66, 146.33, 146.96, 149.14 (Ar-**C**), 160.32, 160.35 (**C**=N, **C**=O). ^{31}P NMR (162 MHz, DMSO-d_6): δ_P = (-153.03) to (-135.47) (m, 2P, 2×**P**F_6). ^{19}F NMR (377 MHz, DMSO-d_6): δ_F = -69.18 (d, 12F, 2×P**F**$_6$). HRMS (ESI) *m*/*z* = 742.2799 [M^+].

Characterization of 1-octyl-4-((2-(1-octylpyridin-1-ium-4-carbonyl)hydrazono)methyl) pyridin-1-ium tetrafluoroborate (17)

It was obtained as yellow crystals in 91% yield; mp: 205-206 ºC. FT-IR (KBr), cm^{-1}: $\bar{\nu}$ = 1549 (C=C), 1638 (C=N), 1686 (C=O), 2848-2914 (Al-H). ^{1}H NMR (400 MHz, DMSO-d_6): δ_H = 0.85 (t, 6H, *J* = 8 Hz, 2×C**H**$_3$), 1.25-1.29 (m, 20H, 10×C**H**$_2$), 1.96 (bs, 4H, 2×NCH$_2$C**H**$_2$), 4.60-4.72 (m, 4H, 2×NC**H**$_2$), 8.20 (d, 0.5H, *J* = 8 Hz, Ar-**H**), 8.30 (s, 0.25H, **H**-C=N), 8.43 (d, 0.5H, *J* = 8 Hz, Ar-**H**), 8.46 (d, 1.5H, *J* = 8 Hz, Ar-**H**), 8.56 (d, 1.5H, *J* = 4 Hz, Ar-**H**), 8.64 (s, 0.75H, **H**-C=N), 9.04 (d, 0.5H, *J* = 8 Hz, Ar-**H**), 9.16 (d, 1.5H, *J* = 8 Hz, Ar-**H**), 9.28 (d, 0.5H, *J* = 4 Hz, Ar-**H**), 9.38 (d, 1.5H, *J* = 4 Hz, Ar-**H**), 13.20 (s, 1H, CON**H**). ^{13}C NMR (100 MHz, DMSO-d_6): δ_C = 14.43 (2×**C**H_3), 22.52, 25.87, 28.82, 28.92, 31.15, 31.60 (12×**C**H_2), 60.96, 61.62 (2×N**C**H_2), 125.26, 125.42, 126.91, 127.74, 144.95, 145.37, 145.66, 146.33, 146.96, 149.14 (Ar-**C**), 160.32, 160.35 (**C**=N, **C**=O). ^{11}B NMR (128 MHz, DMSO-d_6): δ_B = (-1.39) to (-1.23) (m, 2B, 2×**B**F_4). ^{19}F NMR (377 MHz, DMSO-d_6): δ_F = -148.20, -148.14 (2d, 8F, 2×B**F**$_4$). HRMS (ESI) *m*/*z* = 626.3573 [M^+].

Characterization of 1-octyl-4-((2-(1-octylpyridin-1-ium-4-carbonyl)hydrazono)methyl) pyridin-1-ium trifluoroacetate (18)

It was obtained as yellow crystals in 95% yield; mp: 196-197 ºC. FT-IR (KBr), cm^{-1}: $\bar{\nu}$ = 1550 (C=C), 1640 (C=N), 1690 (C=O), 2847-2911 (Al-H). ^{1}H NMR (400 MHz, DMSO-d_6): δ_H = 0.85 (t, 6H, *J* = 8 Hz, 2×C**H**$_3$), 1.25-1.29 (m, 20H, 10×C**H**$_2$), 1.96 (bs, 4H, 2×NCH$_2$C**H**$_2$), 4.60-4.72 (m, 4H, 2×NC**H**$_2$), 8.20 (d, 0.5H, *J* = 8 Hz, Ar-**H**), 8.30 (s, 0.25H, **H**-C=N), 8.43 (d, 0.5H, *J* = 8 Hz, Ar-**H**), 8.46 (d, 1.5H, *J* = 8 Hz, Ar-**H**), 8.56 (d, 1.5H, *J* = 4 Hz, Ar-**H**), 8.64 (s, 0.75H, **H**-C=N), 9.04 (d, 0.5H, *J* = 8 Hz, Ar-**H**), 9.16 (d, 1.5H, *J* = 8 Hz, Ar-**H**), 9.28 (d, 0.5H, *J* = 4 Hz, Ar-**H**), 9.38 (d, 1.5H, *J* = 4 Hz, Ar-**H**),

13.20 (s, 1H, CON**H**). ^{13}C NMR (100 MHz, DMSO-*d*$_6$): δ_C = 14.43 (2×**C**H$_3$), 22.52, 25.87, 28.82, 28.92, 31.15, 31.60 (12×**C**H$_2$), 60.96, 61.62 (2×N**C**H$_2$), 125.26, 125.42, 126.91, 127.74, 144.95, 145.37, 145.66, 146.33, 146.96, 149.14 (Ar-**C**), 160.32, 160.35 (**C**=N, **C**=O). ^{19}F NMR (377 MHz, DMSO-*d*$_6$): δ_F = -73.63 (s, 6F, 2×C**F**$_3$). HRMS (ESI) m/z = 678.3216 [M$^+$].

Characterization of 1-nonyl-4-((2-(1-nonylpyridin-1-ium-4-carbonyl)hydrazono) methyl)pyridin-1-ium hexafluorophosphate (19)

It was obtained as yellow crystals in 89% yield; mp: 202-203 ºC. FT-IR (KBr), cm^{-1}: $\bar{\nu}$ = 1544 (C=C), 1636 (C=N), 1685 (C=O), 2844-2910 (Al-H). ^{1}H NMR (400 MHz, DMSO-*d*$_6$): δ_H = 0.85 (t, 6H, *J* = 8 Hz, 2×C**H**$_3$), 1.23-1.29 (m, 24H, 12×C**H**$_2$), 1.95 (bs, 4H, 2×NCH$_2$C**H**$_2$), 4.59-4.70 (m, 4H, 2×NC**H**$_2$), 8.17 (d, 0.5H, *J* = 4 Hz, Ar-**H**), 8.29 (s, 0.25H, **H**-C=N), 8.43 (d, 0.5H, *J* = 8 Hz, Ar-**H**), 8.45 (d, 1.5H, *J* = 4 Hz, Ar-**H**), 8.56 (d, 1.5H, *J* = 8 Hz, Ar-**H**), 8.63 (s, 0.75H, **H**-C=N), 9.02 (d, 0.5H, *J* = 8 Hz, Ar-**H**), 9.14 (d, 1.5H, *J* = 8 Hz, Ar-**H**), 9.26 (d, 0.5H, *J* = 4 Hz, Ar-**H**), 9.36 (d, 1.5H, *J* = 8 Hz, Ar-**H**), 13.18 (s, 1H, CON**H**). ^{13}C NMR (100 MHz, DMSO-*d*$_6$): δ_C = 14.44 (2×**C**H$_3$), 22.57, 25.88, 25.91, 28.87, 29.18, 29.27, 29.39, 29.48, 29.53, 31.16, 31.76 (14×**C**H$_2$), 61.67, 61.72 (2×N**C**H$_2$), 125.26, 125.42, 126.91, 127.74, 144.95, 145.37, 145.66, 146.33, 146.96, 149.14 (Ar-**C**), 160.32, 160.35 (**C**=N, **C**=O). ^{31}P NMR (162 MHz, DMSO-*d*$_6$): δ_P = (-153.19) to (-135.75) (m, 2P, 2×**P**F$_6$). ^{19}F NMR (377 MHz, DMSO-*d*$_6$): δ_F = -69.19 (d, 12F, 2×P**F**$_6$). HRMS (ESI) *m*/*z* = 770.3112 [M$^+$].

Characterization of 1-nonyl-4-((2-(1-nonylpyridin-1-ium-4-carbonyl)hydrazono) methyl)pyridin-1-ium tetrafluoroborate (20)

It was obtained as yellow crystals in 96% yield; mp: 210-211 ºC. FT-IR (KBr), cm^{-1}: $\bar{\nu}$ = 1552 (C=C), 1639 (C=N), 1692 (C=O), 2849-2917 (Al-H). ^{1}H NMR (400 MHz, DMSO-*d*$_6$): δ_H = 0.85 (t, 6H, *J* = 8 Hz, 2×C**H**$_3$), 1.23-1.29 (m, 24H, 12×C**H**$_2$), 1.95 (bs, 4H, 2×NCH$_2$C**H**$_2$), 4.59-4.70 (m, 4H, 2×NC**H**$_2$), 8.17 (d, 0.5H, *J* = 4 Hz, Ar-**H**), 8.29 (s, 0.25H, **H**-C=N), 8.43 (d, 0.5H, *J* = 8 Hz, Ar-**H**), 8.45 (d, 1.5H, *J* = 4 Hz, Ar-**H**), 8.56 (d, 1.5H, *J* = 8 Hz, Ar-**H**), 8.63 (s, 0.75H, **H**-C=N), 9.02 (d, 0.5H, *J* = 8 Hz, Ar-**H**), 9.14 (d, 1.5H, *J* = 8 Hz, Ar-**H**), 9.26 (d, 0.5H, *J* = 4 Hz, Ar-**H**), 9.36 (d, 1.5H, *J* = 8 Hz, Ar-**H**),

13.18 (s, 1H, CON**H**). ^{13}C NMR (100 MHz, DMSO-d_6): δ_C = 14.44 (2×**C**H_3), 22.57, 25.88, 25.91, 28.87, 29.18, 29.27, 29.39, 29.48, 29.53, 31.16, 31.76 (14×**C**H_2), 61.67, 61.72 (2×N**C**H_2), 125.26, 125.42, 126.91, 127.74, 144.95, 145.37, 145.66, 146.33, 146.96, 149.14 (Ar-**C**), 160.32, 160.35 (**C**=N, **C**=O). ^{11}B NMR (128 MHz, DMSO-d_6): δ_B = (-1.42) to (-1.23) (m, 2B, 2×**B**F_4). ^{19}F NMR (377 MHz, DMSO-d_6): δ_F = -148.21, -148.14 (2d, 8F, 2×B**F**$_4$). HRMS (ESI) m/z = 654.3886 [M$^+$].

Characterization of 1-nonyl-4-((2-(1-nonylpyridin-1-ium-4-carbonyl)hydrazono) methyl)pyridin-1-ium trifluoroacetate (21)

It was obtained as yellow crystals in 95% yield; mp: 188-189 ºC. FT-IR (KBr), cm^{-1}: $\bar{\nu}$ = 1550 (C=C), 1638 (C=N), 1689 (C=O), 2847-2917 (Al-H). ^{1}H NMR (400 MHz, DMSO-d_6): δ_H = 0.85 (t, 6H, J = 8 Hz, 2×C**H**$_3$), 1.23-1.29 (m, 24H, 12×C**H**$_2$), 1.95 (bs, 4H, 2×NCH$_2$C**H**$_2$), 4.59-4.70 (m, 4H, 2×NC**H**$_2$), 8.17 (d, 0.5H, J = 4 Hz, Ar-**H**), 8.29 (s, 0.25H, **H**-C=N), 8.43 (d, 0.5H, J = 8 Hz, Ar-**H**), 8.45 (d, 1.5H, J = 4 Hz, Ar-**H**), 8.56 (d, 1.5H, J = 8 Hz, Ar-**H**), 8.63 (s, 0.75H, **H**-C=N), 9.02 (d, 0.5H, J = 8 Hz, Ar-**H**), 9.14 (d, 1.5H, J = 8 Hz, Ar-**H**), 9.26 (d, 0.5H, J = 4 Hz, Ar-**H**), 9.36 (d, 1.5H, J = 8 Hz, Ar-**H**), 13.18 (s, 1H, CON**H**). ^{13}C NMR (100 MHz, DMSO-d_6): δ_C = 14.44 (2×**C**H_3), 22.57, 25.88, 25.91, 28.87, 29.18, 29.27, 29.39, 29.48, 29.53, 31.16, 31.76 (14×**C**H_2), 61.67, 61.72 (2×N**C**H_2), 125.26, 125.42, 126.91, 127.74, 144.95, 145.37, 145.66, 146.33, 146.96, 149.14 (Ar-**C**), 160.32, 160.35 (**C**=N, **C**=O). ^{19}F NMR (377 MHz, DMSO-d_6): δ_F = -73.59 (s, 6F, 2×C**F**$_3$). HRMS (ESI) m/z = 706.3529 [M$^+$].

Characterization of 1-decyl-4-((2-(1-decylpyridin-1-ium-4-carbonyl)hydrazono) methyl)pyridin-1-ium hexafluorophosphate (22)

It was obtained as yellow crystals in 93% yield; mp: 135-136 ºC. FT-IR (KBr), cm^{-1}: $\bar{\nu}$ = 1553 (C=C), 1640 (C=N), 1688 (C=O), 2846-2915 (Al-H). ^{1}H NMR (400 MHz, DMSO-d_6): δ_H = 0.84 (t, 6H, J = 4 Hz, 2×C**H**$_3$), 1.24-1.29 (m, 28H, 14×C**H**$_2$), 1.95 (bs, 4H, 2×NCH$_2$C**H**$_2$), 4.59-4.71 (m, 4H, 2×NC**H**$_2$), 8.19 (d, 0.5H, J = 4 Hz, Ar-**H**), 8.30 (s, 0.25H, **H**-C=N), 8.43 (d, 0.5H, J = 8 Hz, Ar-**H**), 8.46 (d, 1.5H, J = 4 Hz, Ar-**H**), 8.56 (d, 1.5H, J = 4 Hz, Ar-**H**), 8.63 (s, 0.75H, **H**-C=N), 9.02 (d, 0.5H, J = 4 Hz, Ar-**H**), 9.15 (d, 1.5H, J = 4 Hz, Ar-**H**), 9.28 (d, 0.5H, J = 4 Hz, Ar-**H**), 9.37 (d, 1.5H, J = 4 Hz, Ar-**H**),

13.20 (s, 1H, CONH). ^{13}C NMR (100 MHz, DMSO-d_6): δ_C = 14.45 (2×**C**H_3), 22.57, 25.87, 25.91, 28.86, 29.13, 29.27, 29.35, 29.48, 31.15, 31.75 (16×**C**H_2), 60.95, 61.62 (2×N**C**H_2), 125.26, 125.42, 126.91, 127.74, 144.95, 145.37, 145.66, 146.33, 146.96, 149.14 (Ar-**C**), 160.25, 160.33 (**C**=N, **C**=O). ^{31}P NMR (162 MHz, DMSO-d_6): δ_P = (-153.03) to (-135.47) (m, 2P, 2×**P**F_6). ^{19}F NMR (377 MHz, DMSO-d_6): δ_F = -69.19 (d, 12F, 2×P**F**$_6$). HRMS (ESI) m/z = 798.3425 [M$^+$].

Characterization of 1-decyl-4-((2-(1-decylpyridin-1-ium-4-carbonyl)hydrazono) methyl)pyridin-1-ium tetrafluoroborate (23)

It was obtained as yellow crystals in 88% yield; mp: 189-190 ºC. FT-IR (KBr), cm^{-1}: $\bar{\nu}$ = 1548 (C=C), 1639 (C=N), 1685 (C=O), 2842-2910 (Al-H). ^{1}H NMR (400 MHz, DMSO-d_6): δ_H = 0.84 (t, 6H, J = 4 Hz, 2×C**H**$_3$), 1.24-1.29 (m, 28H, 14×C**H**$_2$), 1.95 (bs, 4H, 2×NCH$_2$C**H**$_2$), 4.59-4.71 (m, 4H, 2×NC**H**$_2$), 8.19 (d, 0.5H, J = 4 Hz, Ar-**H**), 8.30 (s, 0.25H, **H**-C=N), 8.43 (d, 0.5H, J = 8 Hz, Ar-**H**), 8.46 (d, 1.5H, J = 4 Hz, Ar-**H**), 8.56 (d, 1.5H, J = 4 Hz, Ar-**H**), 8.63 (s, 0.75H, **H**-C=N), 9.02 (d, 0.5H, J = 4 Hz, Ar-**H**), 9.15 (d, 1.5H, J = 4 Hz, Ar-**H**), 9.28 (d, 0.5H, J = 4 Hz, Ar-**H**), 9.37 (d, 1.5H, J = 4 Hz, Ar-**H**), 13.20 (s, 1H, CON**H**). ^{13}C NMR (100 MHz, DMSO-d_6): δ_C = 14.45 (2×**C**H_3), 22.57, 25.87, 25.91, 28.86, 29.13, 29.27, 29.35, 29.48, 31.15, 31.75 (16×**C**H_2), 60.95, 61.62 (2×N**C**H_2), 125.26, 125.42, 126.91, 127.74, 144.95, 145.37, 145.66, 146.33, 146.96, 149.14 (Ar-**C**), 160.25, 160.33 (**C**=N, **C**=O). ^{11}B NMR (128 MHz, DMSO-d_6): δ_B = (-1.40) to (-1.24) (m, 2B, 2×**B**F_4). ^{19}F NMR (377 MHz, DMSO-d_6): δ_F = -148.21, -148.14 (2d, 8F, 2×B**F**$_4$). HRMS (ESI) m/z = 682.4199 [M$^+$].

Characterization of 1-decyl-4-((2-(1-decylpyridin-1-ium-4-carbonyl)hydrazono) methyl)pyridin-1-ium trifluoroacetate (24)

It was obtained as yellow crystals in 89% yield; mp: 185-186 ºC. FT-IR (KBr), cm^{-1}: $\bar{\nu}$ = 1550 (C=C), 1636 (C=N), 1691 (C=O), 2846-2918 (Al-H). ^{1}H NMR (400 MHz, DMSO-d_6): δ_H = 0.84 (t, 6H, J = 4 Hz, 2×C**H**$_3$), 1.24-1.29 (m, 28H, 14×C**H**$_2$), 1.95 (bs, 4H, 2×NCH$_2$C**H**$_2$), 4.59-4.71 (m, 4H, 2×NC**H**$_2$), 8.19 (d, 0.5H, J = 4 Hz, Ar-**H**), 8.30 (s, 0.25H, **H**-C=N), 8.43 (d, 0.5H, J = 8 Hz, Ar-**H**), 8.46 (d, 1.5H, J = 4 Hz, Ar-**H**), 8.56 (d, 1.5H, J = 4 Hz, Ar-**H**), 8.63 (s, 0.75H, **H**-C=N), 9.02 (d, 0.5H, J = 4 Hz, Ar-**H**), 9.15 (d,

1.5H, J = 4 Hz, Ar-**H**), 9.28 (d, 0.5H, J = 4 Hz, Ar-**H**), 9.37 (d, 1.5H, J = 4 Hz, Ar-**H**), 13.20 (s, 1H, CON**H**). ^{13}C NMR (100 MHz, DMSO-d_6): δ_C = 14.45 (2×**C**H$_3$), 22.57, 25.87, 25.91, 28.86, 29.13, 29.27, 29.35, 29.48, 31.15, 31.75 (16×**C**H$_2$), 60.95, 61.62 (2×N**C**H$_2$), 125.26, 125.42, 126.91, 127.74, 144.95, 145.37, 145.66, 146.33, 146.96, 149.14 (Ar-**C**), 160.25, 160.33 (**C**=N, **C**=O). ^{19}F NMR (377 MHz, DMSO-d_6): δ_F = -73.61 (s, 6F, 2×C**F**$_3$). HRMS (ESI) m/z = 734.3842 [M$^+$].

Characterization of 1-dodecyl-4-((2-(1-dodecylpyridin-1-ium-4-carbonyl)hydrazono) methyl)pyridin-1-ium hexafluorophosphate (25)

It was obtained as yellow crystals in 90% yield; mp: 184-185 ºC. FT-IR (KBr), cm^{-1}: $\bar{\nu}$ = 1547 (C=C), 1639 (C=N), 1690 (C=O), 2846-2913 (Al-H). ^{1}H NMR (400 MHz, DMSO-d_6): δ_H = 0.84 (t, 6H, J = 4 Hz, 2×C**H**$_3$), 1.23-1.29 (m, 36H, 18×C**H**$_2$), 1.95 (bs, 4H, 2×NCH$_2$C**H**$_2$), 4.59-4.71 (m, 4H, 2×NC**H**$_2$), 8.18 (d, 0.5H, J = 4 Hz, Ar-**H**), 8.30 (s, 0.25H, **H**-C=N), 8.43 (d, 0.5H, J = 8 Hz, Ar-**H**), 8.45 (d, 1.5H, J = 4 Hz, Ar-**H**), 8.56 (d, 1.5H, J = 4 Hz, Ar-**H**), 8.63 (s, 0.75H, **H**-C=N), 9.03 (d, 0.5H, J = 8 Hz, Ar-**H**), 9.15 (d, 1.5H, J = 8 Hz, Ar-**H**), 9.28 (d, 0.5H, J = 4 Hz, Ar-**H**), 9.37 (d, 1.5H, J = 4 Hz, Ar-**H**), 13.20 (s, 1H, CON**H**). ^{13}C NMR (100 MHz, DMSO-d_6): δ_C = 14.44 (2×**C**H$_3$), 22.57, 25.86, 29.19, 29.27, 29.38, 29.39, 29.48, 31.15, 31.76 (20×**C**H$_2$), 60.95, 61.62 (2×N**C**H$_2$), 125.26, 125.42, 126.91, 127.74, 144.93, 145.37, 145.66, 146.35, 146.96, 149.17 (Ar-**C**), 160.25, 160.33 (**C**=N, **C**=O). ^{31}P NMR (162 MHz, DMSO-d_6): δ_P = (-153.02) to (-135.45) (m, 2P, 2×**P**F$_6$). ^{19}F NMR (377 MHz, DMSO-d_6): δ_F = -69.19 (d, 12F, 2×P**F**$_6$). HRMS (ESI) m/z = 854.4051 [M$^+$].

Characterization of 1-dodecyl-4-((2-(1-dodecylpyridin-1-ium-4-carbonyl)hydrazono) methyl)pyridin-1-ium tetrafluoroborate (26)

It was obtained as yellow crystals in 95% yield; mp: 193-194 ºC. FT-IR (KBr), cm^{-1}: $\bar{\nu}$ = 1547 (C=C), 1640 (C=N), 1689 (C=O), 2845-2919 (Al-H). ^{1}H NMR (400 MHz, DMSO-d_6): δ_H = 0.84 (t, 6H, J = 4 Hz, 2×C**H**$_3$), 1.23-1.29 (m, 36H, 18×C**H**$_2$), 1.95 (bs, 4H, 2×NCH$_2$C**H**$_2$), 4.59-4.71 (m, 4H, 2×NC**H**$_2$), 8.18 (d, 0.5H, J = 4 Hz, Ar-**H**), 8.30 (s, 0.25H, **H**-C=N), 8.43 (d, 0.5H, J = 8 Hz, Ar-**H**), 8.45 (d, 1.5H, J = 4 Hz, Ar-**H**), 8.56 (d, 1.5H, J = 4 Hz, Ar-**H**), 8.63 (s, 0.75H, **H**-C=N), 9.03 (d, 0.5H, J = 8 Hz, Ar-**H**), 9.15 (d,

1.5H, *J* = 8 Hz, Ar-**H**), 9.28 (d, 0.5H, *J* = 4 Hz, Ar-**H**), 9.37 (d, 1.5H, *J* = 4 Hz, Ar-**H**), 13.20 (s, 1H, CON**H**). ^{13}C NMR (100 MHz, DMSO-d_6): δ_C = 14.44 (2×**C**H$_3$), 22.57, 25.86, 29.19, 29.27, 29.38, 29.39, 29.48, 31.15, 31.76 (20×**C**H$_2$), 60.95, 61.62 (2×N**C**H$_2$), 125.26, 125.42, 126.91, 127.74, 144.93, 145.37, 145.66, 146.35, 146.96, 149.17 (Ar-**C**), 160.25, 160.33 (**C**=N, **C**=O). ^{11}B NMR (128 MHz, DMSO-d_6): δ_B = (-1.41) to (-1.24) (m, 2B, 2×**B**F$_4$). ^{19}F NMR (377 MHz, DMSO-d_6): δ_F = -148.20, -148.14 (2d, 8F, 2×B**F**$_4$). HRMS (ESI) *m/z* = 738.4825 [M$^+$].

Characterization of 1-dodecyl-4-((2-(1-dodecylpyridin-1-ium-4-carbonyl)hydrazono) methyl)pyridin-1-ium trifluoroacetate (27)

It was obtained as yellow crystals in 91% yield; mp: 179-180 ºC. FT-IR (KBr), cm^{-1}: $\bar{\nu}$ = 1546 (C=C), 1641 (C=N), 1692 (C=O), 2845-2918 (Al-H). ^{1}H NMR (400 MHz, DMSO-d_6): δ_H = 0.84 (t, 6H, *J* = 4 Hz, 2×C**H**$_3$), 1.23-1.29 (m, 36H, 18×C**H**$_2$), 1.95 (bs, 4H, 2×NCH$_2$C**H**$_2$), 4.59-4.71 (m, 4H, 2×NC**H**$_2$), 8.18 (d, 0.5H, *J* = 4 Hz, Ar-**H**), 8.30 (s, 0.25H, **H**-C=N), 8.43 (d, 0.5H, *J* = 8 Hz, Ar-**H**), 8.45 (d, 1.5H, *J* = 4 Hz, Ar-**H**), 8.56 (d, 1.5H, *J* = 4 Hz, Ar-**H**), 8.63 (s, 0.75H, **H**-C=N), 9.03 (d, 0.5H, *J* = 8 Hz, Ar-**H**), 9.15 (d, 1.5H, *J* = 8 Hz, Ar-**H**), 9.28 (d, 0.5H, *J* = 4 Hz, Ar-**H**), 9.37 (d, 1.5H, *J* = 4 Hz, Ar-**H**), 13.20 (s, 1H, CON**H**). ^{13}C NMR (100 MHz, DMSO-d_6): δ_C = 14.44 (2×**C**H$_3$), 22.57, 25.86, 29.19, 29.27, 29.38, 29.39, 29.48, 31.15, 31.76 (20×**C**H$_2$), 60.95, 61.62 (2×N**C**H$_2$), 125.26, 125.42, 126.91, 127.74, 144.93, 145.37, 145.66, 146.35, 146.96, 149.17 (Ar-**C**), 160.25, 160.33 (**C**=N, **C**=O). ^{19}F NMR (377 MHz, DMSO-d_6): δ_F = -73.65 (s, 6F, 2×C**F**$_3$). HRMS (ESI) m/z = 790.4468 [M$^+$].

Characterization of 1-tetradecyl-4-((2-(1-tetradecylpyridin-1-ium-4-carbonyl) hydrazono)methyl)pyridin-1-ium hexafluorophosphate (28)

It was obtained as yellow crystals in 92% yield; mp: 180-181 ºC. FT-IR (KBr), cm^{-1}: $\bar{\nu}$ = 1553 (C=C), 1642 (C=N), 1692 (C=O), 2846-2917 (Al-H). ^{1}H NMR (400 MHz, DMSO-d_6): δ_H = 0.84 (t, 6H, *J* = 4 Hz, 2×C**H**$_3$), 1.24-1.29 (m, 44H, 22×C**H**$_2$), 1.96 (bs, 4H, 2×NCH$_2$C**H**$_2$), 4.60-4.72 (m, 4H, 2×NC**H**$_2$), 8.20 (d, 0.5H, *J* = 4 Hz, Ar-**H**), 8.30 (s, 0.25H, **H**-C=N), 8.43 (d, 0.5H, *J* = 8 Hz, Ar-**H**), 8.46 (d, 1.5H, *J* = 8 Hz, Ar-**H**), 8.56 (d, 1.5H, *J* = 4 Hz, Ar-**H**), 8.64 (s, 0.75H, **H**-C=N), 9.04 (d, 0.5H, *J* = 4 Hz, Ar-**H**), 9.16 (d,

1.5H, J = 4 Hz, Ar-**H**), 9.29 (d, 0.5H, J = 4 Hz, Ar-**H**), 9.39 (d, 1.5H, J = 8 Hz, Ar-**H**), 13.19 (s, 1H, CON**H**). ^{13}C NMR (100 MHz, DMSO-d_6): δ_C = 14.44 (2×**C**H_3), 22.56, 25.86, 28.86, 29.09, 29.22, 29.38, 29.48, 31.15, 31.69 (24×**C**H_2), 60.94, 61.61 (2×N**C**H_2), 125.26, 125.41, 126.90, 127.72, 144.93, 145.34, 145.65, 146.35, 146.98, 149.14 (Ar-**C**), 160.33, 160.40 (**C**=N, **C**=O). ^{31}P NMR (162 MHz, DMSO-d_6): δ_P = (-153.31) to (-135.84) (m, 2P, 2×**P**F_6). ^{19}F NMR (377 MHz, DMSO-d_6): δ_F = -69.19 (d, 12F, 2×P$\mathbf{F_6}$). HRMS (ESI) m/z = 910.4677 [M^+].

Characterization of 1-tetradecyl-4-((2-(1-tetradecylpyridin-1-ium-4-carbonyl) hydrazono)methyl)pyridin-1-ium tetrafluoroborate (29)

It was obtained as yellow crystals in 92% yield; mp: 183-184 ºC. FT-IR (KBr), cm^{-1}: $\bar{\nu}$ = 1548 (C=C), 1639 (C=N), 1689 (C=O), 2846-2917 (Al-H). ^{1}H NMR (400 MHz, DMSO-d_6): δ_H = 0.84 (t, 6H, J = 4 Hz, 2×C$\mathbf{H_3}$), 1.24-1.29 (m, 44H, 22×C$\mathbf{H_2}$), 1.96 (bs, 4H, 2×NCH_2C$\mathbf{H_2}$), 4.60-4.72 (m, 4H, 2×NC$\mathbf{H_2}$), 8.20 (d, 0.5H, J = 4 Hz, Ar-**H**), 8.30 (s, 0.25H, **H**-C=N), 8.43 (d, 0.5H, J = 8 Hz, Ar-**H**), 8.46 (d, 1.5H, J = 8 Hz, Ar-**H**), 8.56 (d, 1.5H, J = 4 Hz, Ar-**H**), 8.64 (s, 0.75H, **H**-C=N), 9.04 (d, 0.5H, J = 4 Hz, Ar-**H**), 9.16 (d, 1.5H, J = 4 Hz, Ar-**H**), 9.29 (d, 0.5H, J = 4 Hz, Ar-**H**), 9.39 (d, 1.5H, J = 8 Hz, Ar-**H**), 13.19 (s, 1H, CON**H**). ^{13}C NMR (100 MHz, DMSO-d_6): δ_C = 14.44 (2×**C**H_3), 22.56, 25.86, 28.86, 29.09, 29.22, 29.38, 29.48, 31.15, 31.69 (24×**C**H_2), 60.94, 61.61 (2×N**C**H_2), 125.26, 125.41, 126.90, 127.72, 144.93, 145.34, 145.65, 146.35, 146.98, 149.14 (Ar-**C**), 160.33, 160.40 (**C**=N, **C**=O). ^{11}B NMR (128 MHz, DMSO-d_6): δ_B = (-1.39) to (-1.24) (m, 2B, 2×**B**F_4). ^{19}F NMR (377 MHz, DMSO-d_6): δ_F = -148.21, -148.15 (2d, 8F, 2×B$\mathbf{F_4}$). HRMS (ESI) m/z = 794.5451 [M^+].

Characterization of 1-tetradecyl-4-((2-(1-tetradecylpyridin-1-ium-4-carbonyl) hydrazono)methyl)pyridin-1-ium trifluoroacetate (30)

It was obtained as yellow crystals in 89% yield; mp: 171-172 ºC. FT-IR (KBr), cm^{-1}: $\bar{\nu}$ = 1550 (C=C), 1638 (C=N), 1691 (C=O), 2845-2917 (Al-H). ^{1}H NMR (400 MHz, DMSO-d_6): δ_H = 0.84 (t, 6H, J = 4 Hz, 2×C$\mathbf{H_3}$), 1.24-1.29 (m, 44H, 22×C$\mathbf{H_2}$), 1.96 (bs, 4H, 2×NCH_2C$\mathbf{H_2}$), 4.60-4.72 (m, 4H, 2×NC$\mathbf{H_2}$), 8.20 (d, 0.5H, J = 4 Hz, Ar-**H**), 8.30 (s, 0.25H, **H**-C=N), 8.43 (d, 0.5H, J = 8 Hz, Ar-**H**), 8.46 (d, 1.5H, J = 8 Hz, Ar-**H**), 8.56 (d,

1.5H, *J* = 4 Hz, Ar-**H**), 8.64 (s, 0.75H, **H**-C=N), 9.04 (d, 0.5H, *J* = 4 Hz, Ar-**H**), 9.16 (d, 1.5H, *J* = 4 Hz, Ar-**H**), 9.29 (d, 0.5H, *J* = 4 Hz, Ar-**H**), 9.39 (d, 1.5H, *J* = 8 Hz, Ar-**H**), 13.19 (s, 1H, CON**H**). ^{13}C NMR (100 MHz, DMSO-d_6): δ_C = 14.44 (2×**C**H_3), 22.56, 25.86, 28.86, 29.09, 29.22, 29.38, 29.48, 31.15, 31.69 (24×**C**H_2), 60.94, 61.61 (2×N**C**H_2), 125.26, 125.41, 126.90, 127.72, 144.93, 145.34, 145.65, 146.35, 146.98, 149.14 (Ar-**C**), 160.33, 160.40 (**C**=N, **C**=O). ^{19}F NMR (377 MHz, DMSO-d_6): δ_F = -73.59 (s, 6F, 2×C**F**$_3$). HRMS (ESI) m/z = 846.5094 [M^+].

Characterization of 1-hexadecyl-4-((2-(1-hexadecylpyridin-1-ium-4-carbonyl) hydrazono)methyl)pyridin-1-ium hexafluorophosphate (31)

It was obtained as yellow crystals in 90% yield; mp: 169-170 ºC. FT-IR (KBr), cm^{-1}: $\bar{\nu}$ = 1547 (C=C), 1640 (C=N), 1689 (C=O), 2844-2911 (Al-H). ^{1}H NMR (400 MHz, DMSO-d_6): δ_H = 0.84 (t, 6H, *J* = 8 Hz, 2×C**H**$_3$), 1.22-1.28 (m, 52H, 26×C**H**$_2$), 1.95 (bs, 4H, 2×NCH$_2$C**H**$_2$), 4.57-4.69 (m, 4H, 2×NC**H**$_2$), 8.15 (d, 0.5H, *J* = 4 Hz, Ar-**H**), 8.28 (s, 0.25H, **H**-C=N), 8.40 (d, 0.5H, *J* = 8 Hz, Ar-**H**), 8.44 (d, 1.5H, *J* = 8 Hz, Ar-**H**), 8.55 (d, 1.5H, *J* = 8 Hz, Ar-**H**), 8.61 (s, 0.75H, **H**-C=N), 8.99 (d, 0.5H, *J* = 4 Hz, Ar-**H**), 9.12 (d, 1.5H, *J* = 4 Hz, Ar-**H**), 9.24 (d, 0.5H, *J* = 4 Hz, Ar-**H**), 9.33 (d, 1.5H, *J* = 8 Hz, Ar-**H**), 13.19 (s, 1H, CON**H**). ^{13}C NMR (100 MHz, DMSO-d_6): δ_C = 14.43 (2×**C**H_3), 22.55, 25.85, 29.16, 29.25, 29.38, 29.46, 29.50, 31.15, 31.75 (28×**C**H_2), 60.94, 61.61 (2×N**C**H_2), 125.26, 125.41, 126.90, 127.72, 144.93, 145.34, 145.65, 146.35, 146.98, 149.14 (Ar-**C**), 160.35, 161.33 (**C**=N, **C**=O). ^{31}P NMR (162 MHz, DMSO-d_6): δ_P = (-153.29) to (-135.70) (m, 2P, 2×**P**F_6). ^{19}F NMR (377 MHz, DMSO-d_6): δ_F = -69.19 (d, 12F, 2×P**F**$_6$). HRMS (ESI) m/z = 966.5303 [M^+].

Characterization of 1-hexadecyl-4-((2-(1-hexadecylpyridin-1-ium-4-carbonyl) hydrazono)methyl)pyridin-1-ium tetrafluoroborate (32)

It was obtained as yellow crystals in 92% yield; mp: 177-178 ºC. FT-IR (KBr), cm^{-1}: $\bar{\nu}$ = 1551 (C=C), 1641 (C=N), 1690 (C=O), 2849-2917 (Al-H). ^{1}H NMR (400 MHz, DMSO-d_6): δ_H = 0.84 (t, 6H, *J* = 8 Hz, 2×C**H**$_3$), 1.22-1.28 (m, 52H, 26×C**H**$_2$), 1.95 (bs, 4H, 2×NCH$_2$C**H**$_2$), 4.57-4.69 (m, 4H, 2×NC**H**$_2$), 8.15 (d, 0.5H, *J* = 4 Hz, Ar-**H**), 8.28 (s, 0.25H, **H**-C=N), 8.40 (d, 0.5H, *J* = 8 Hz, Ar-**H**), 8.44 (d, 1.5H, *J* = 8 Hz, Ar-**H**), 8.55 (d,

1.5H, J = 8 Hz, Ar-**H**), 8.61 (s, 0.75H, **H**-C=N), 8.99 (d, 0.5H, J = 4 Hz, Ar-**H**), 9.12 (d, 1.5H, J = 4 Hz, Ar-**H**), 9.24 (d, 0.5H, J = 4 Hz, Ar-**H**), 9.33 (d, 1.5H, J = 8 Hz, Ar-**H**), 13.19 (s, 1H, CON**H**). ^{13}C NMR (100 MHz, DMSO-d_6): δ_C = 14.43 (2×**C**H_3), 22.55, 25.85, 29.16, 29.25, 29.38, 29.46, 29.50, 31.15, 31.75 (28×**C**H_2), 60.94, 61.61 (2×N**C**H_2), 125.26, 125.41, 126.90, 127.72, 144.93, 145.34, 145.65, 146.35, 146.98, 149.14 (Ar-**C**), 160.35, 161.33 (**C**=N, **C**=O). ^{11}B NMR (128 MHz, DMSO-d_6): δ_B = (-1.39) to (-1.24) (m, 2B, 2×**B**F_4). ^{19}F NMR (377 MHz, DMSO-d_6): δ_F = -148.14, -148.21 (2d, 8F, 2×B**F**$_4$). HRMS (ESI) m/z = 850.6077 [M$^+$].

Characterization of 1-hexadecyl-4-((2-(1-hexadecylpyridin-1-ium-4-carbonyl) hydrazono)methyl)pyridin-1-ium trifluoroacetate (33)

It was obtained as yellow crystals in 94% yield; mp: 173-174 ºC. FT-IR (KBr), cm^{-1}: $\bar{\nu}$ = 1547 (C=C), 1636 (C=N), 1689 (C=O), 2843-2919 (Al-H). ^{1}H NMR (400 MHz, DMSO-d_6): δ_H = 0.84 (t, 6H, J = 8 Hz, 2×C**H**$_3$), 1.22-1.28 (m, 52H, 26×C**H**$_2$), 1.95 (bs, 4H, 2×NCH$_2$C**H**$_2$), 4.57-4.69 (m, 4H, 2×NC**H**$_2$), 8.15 (d, 0.5H, J = 4 Hz, Ar-**H**), 8.28 (s, 0.25H, **H**-C=N), 8.40 (d, 0.5H, J = 8 Hz, Ar-**H**), 8.44 (d, 1.5H, J = 8 Hz, Ar-**H**), 8.55 (d, 1.5H, J = 8 Hz, Ar-**H**), 8.61 (s, 0.75H, **H**-C=N), 8.99 (d, 0.5H, J = 4 Hz, Ar-**H**), 9.12 (d, 1.5H, J = 4 Hz, Ar-**H**), 9.24 (d, 0.5H, J = 4 Hz, Ar-**H**), 9.33 (d, 1.5H, J = 8 Hz, Ar-**H**), 13.19 (s, 1H, CON**H**). ^{13}C NMR (100 MHz, DMSO-d_6): δ_C = 14.43 (2×**C**H_3), 22.55, 25.85, 29.16, 29.25, 29.38, 29.46, 29.50, 31.15, 31.75 (28×**C**H_2), 60.94, 61.61 (2×N**C**H_2), 125.26, 125.41, 126.90, 127.72, 144.93, 145.34, 145.65, 146.35, 146.98, 149.14 (Ar-**C**), 160.35, 161.33 (**C**=N, **C**=O). ^{19}F NMR (377 MHz, DMSO-d_6): δ_F = -73.57 (s, 6F, 2×C**F**$_3$). HRMS (ESI) m/z = 902.5720 [M$^+$].

Characterization of 1-octadecyl-4-((2-(1-octadecylpyridin-1-ium-4-carbonyl) hydrazono)methyl)pyridin-1-ium hexafluorophosphate (34)

It was obtained as yellow crystals in 92% yield; mp: 160-161 ºC. FT-IR (KBr), cm^{-1}: $\bar{\nu}$ = 1545 (C=C), 1637 (C=N), 1693 (C=O), 2846-2918 (Al-H). ^{1}H NMR (400 MHz, DMSO-d_6): δ_H = 0.84 (t, 6H, J = 8 Hz, 2×C**H**$_3$), 1.23-1.29 (m, 60H, 30×C**H**$_2$), 1.94 (bs, 4H, 2×NCH$_2$C**H**$_2$), 4.59-4.69 (m, 4H, 2×NC**H**$_2$), 8.18 (d, 0.5H, J = 8 Hz, Ar-**H**), 8.29 (s, 0.25H, **H**-C=N), 8.43 (d, 0.5H, J = 8 Hz, Ar-**H**), 8.46 (d, 1.5H, J = 8 Hz, Ar-**H**), 8.56 (d,

1.5H, *J* = 8 Hz, Ar-**H**), 8.63 (s, 0.75H, **H**-C=N), 9.02 (d, 0.5H, *J* = 8 Hz, Ar-**H**), 9.14 (d, 1.5H, *J* = 4 Hz, Ar-**H**), 9.28 (d, 0.5H, *J* = 4 Hz, Ar-**H**), 9.36 (d, 1.5H, *J* = 4 Hz, Ar-**H**), 13.18 (s, 1H, CON**H**). ^{13}C NMR (100 MHz, DMSO-d_6): δ_C = 14.44 (2×**C**H_3), 22.57, 25.86, 28.87, 29.18, 29.27, 29.39, 29.48, 31.16, 31.76 (24×**C**H_2), 60.97, 61.65 (2×N**C**H_2), 125.26, 125.41, 126.90, 127.72, 144.93, 145.34, 145.65, 146.35, 146.98, 149.14 (Ar-**C**), 160.33, 160.40 (**C**=N, **C**=O). ^{31}P NMR (162 MHz, DMSO-d_6): δ_P = (-153.29) to (-135.70) (m, 2P, 2×**P**F_6). ^{19}F NMR (377 MHz, DMSO-d_6): δ_F = -69.18 (d, 12F, 2×P**F_6**). HRMS (ESI) m/z = 1022.5929 [M^+].

Characterization of 1-octadecyl-4-((2-(1-octadecylpyridin-1-ium-4-carbonyl) hydrazono)methyl)pyridin-1-ium tetrafluoroborate (35)

It was obtained as yellow crystals in 90% yield; mp: 189-190 ºC. FT-IR (KBr), cm^{-1}: $\bar{\nu}$ = 1549 (C=C), 1642 (C=N), 1693 (C=O), 2846-2914 (Al-H). ^{1}H NMR (400 MHz, DMSO-d_6): δ_H = 0.84 (t, 6H, *J* = 8 Hz, 2×C**H_3**), 1.23-1.29 (m, 60H, 30×C**H_2**), 1.94 (bs, 4H, 2×NCH_2C**H_2**), 4.59-4.69 (m, 4H, 2×NC**H_2**), 8.18 (d, 0.5H, *J* = 8 Hz, Ar-**H**), 8.29 (s, 0.25H, **H**-C=N), 8.43 (d, 0.5H, *J* = 8 Hz, Ar-**H**), 8.46 (d, 1.5H, *J* = 8 Hz, Ar-**H**), 8.56 (d, 1.5H, *J* = 8 Hz, Ar-**H**), 8.63 (s, 0.75H, **H**-C=N), 9.02 (d, 0.5H, *J* = 8 Hz, Ar-**H**), 9.14 (d, 1.5H, *J* = 4 Hz, Ar-**H**), 9.28 (d, 0.5H, *J* = 4 Hz, Ar-**H**), 9.36 (d, 1.5H, *J* = 4 Hz, Ar-**H**), 13.18 (s, 1H, CON**H**). ^{13}C NMR (100 MHz, DMSO-d_6): δ_C = 14.44 (2×**C**H_3), 22.57, 25.86, 28.87, 29.18, 29.27, 29.39, 29.48, 31.16, 31.76 (24×**C**H_2), 60.97, 61.65 (2×N**C**H_2), 125.26, 125.41, 126.90, 127.72, 144.93, 145.34, 145.65, 146.35, 146.98, 149.14 (Ar-**C**), 160.33, 160.40 (**C**=N, **C**=O). ^{11}B NMR (128 MHz, DMSO-d_6): δ_B = (-1.24) to (-1.42) (m, 2B, 2×**B**F_4). ^{19}F NMR (377 MHz, DMSO-d_6): δ_F = -148.13, -148.22 (2d, 8F, 2×B**F_4**). HRMS (ESI) *m/z* = 906.6703 [M^+].

Characterization of 1-octadecyl-4-((2-(1-octadecylpyridin-1-ium-4-carbonyl) hydrazono)methyl)pyridin-1-ium trifluoroacetate (36)

It was obtained as yellow crystals in 94% yield; mp: 165-166 ºC. FT-IR (KBr), cm^{-1}: $\bar{\nu}$ = 1548 (C=C), 1637 (C=N), 1689 (C=O), 2847-2919 (Al-H). ^{1}H NMR (400 MHz, DMSO-d_6): δ_H = 0.84 (t, 6H, *J* = 8 Hz, 2×C**H_3**), 1.23-1.29 (m, 60H, 30×C**H_2**), 1.94 (bs, 4H, 2×NCH_2C**H_2**), 4.59-4.69 (m, 4H, 2×NC**H_2**), 8.18 (d, 0.5H, *J* = 8 Hz, Ar-**H**), 8.29 (s,

0.25H, **H**-C=N), 8.43 (d, 0.5H, *J* = 8 Hz, Ar-**H**), 8.46 (d, 1.5H, *J* = 8 Hz, Ar-**H**), 8.56 (d, 1.5H, *J* = 8 Hz, Ar-**H**), 8.63 (s, 0.75H, **H**-C=N), 9.02 (d, 0.5H, *J* = 8 Hz, Ar-**H**), 9.14 (d, 1.5H, *J* = 4 Hz, Ar-**H**), 9.28 (d, 0.5H, *J* = 4 Hz, Ar-**H**), 9.36 (d, 1.5H, *J* = 4 Hz, Ar-**H**), 13.18 (s, 1H, CON**H**). ^{13}C NMR (100 MHz, DMSO-d_6): δ_C = 14.44 (2×**C**H_3), 22.57, 25.86, 28.87, 29.18, 29.27, 29.39, 29.48, 31.16, 31.76 (24×**C**H_2), 60.97, 61.65 (2×N**C**H_2), 125.26, 125.41, 126.90, 127.72, 144.93, 145.34, 145.65, 146.35, 146.98, 149.14 (Ar-**C**), 160.33, 160.40 (**C**=N, **C**=O). ^{19}F NMR (377 MHz, DMSO-d_6): δ_F = -73.50 (s, 6F, 2×C**F**$_3$). HRMS (ESI) m/z = 958.6346 [M^+].

3. Spectroscopic Data

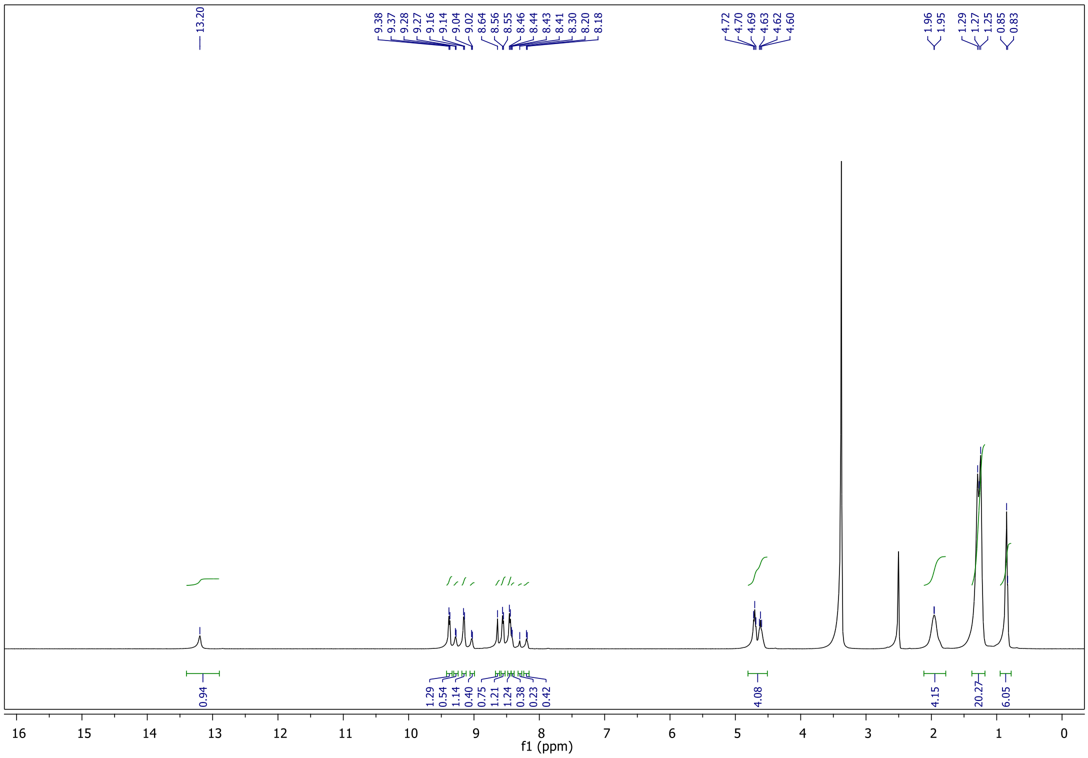

Figure S1: ^{1}H NMR of Compound **9**

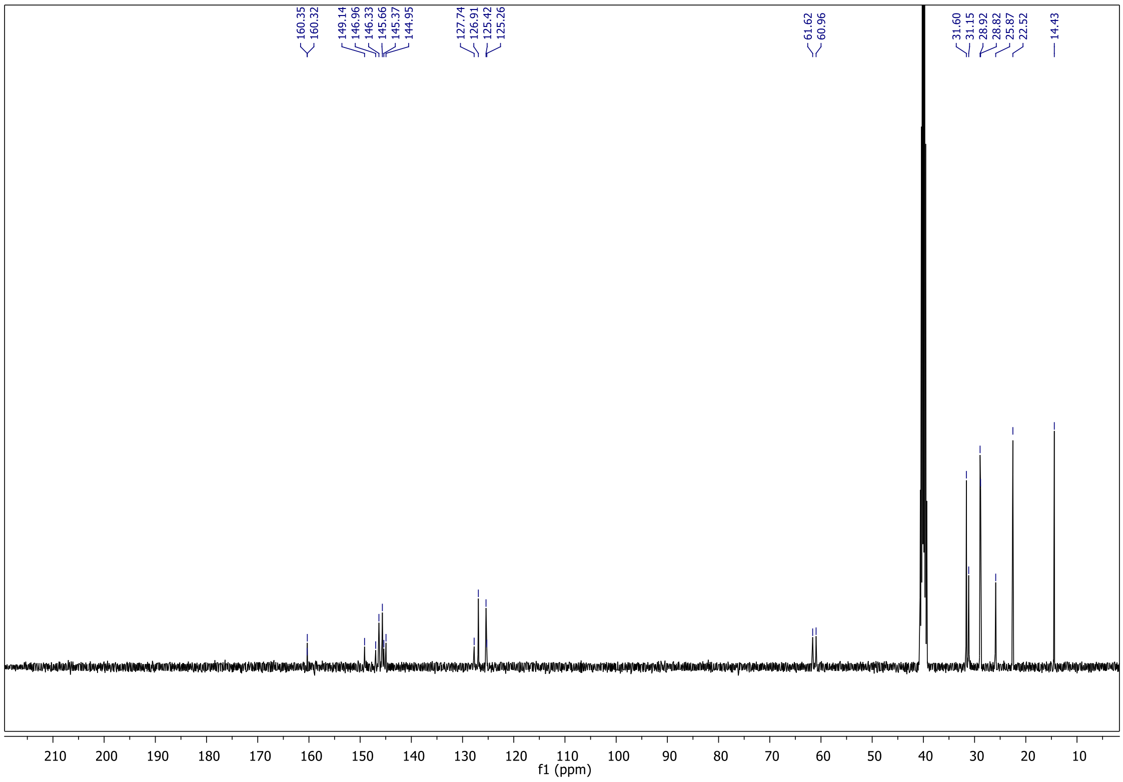

Figure S2: ^{13}C NMR of Compound **9**

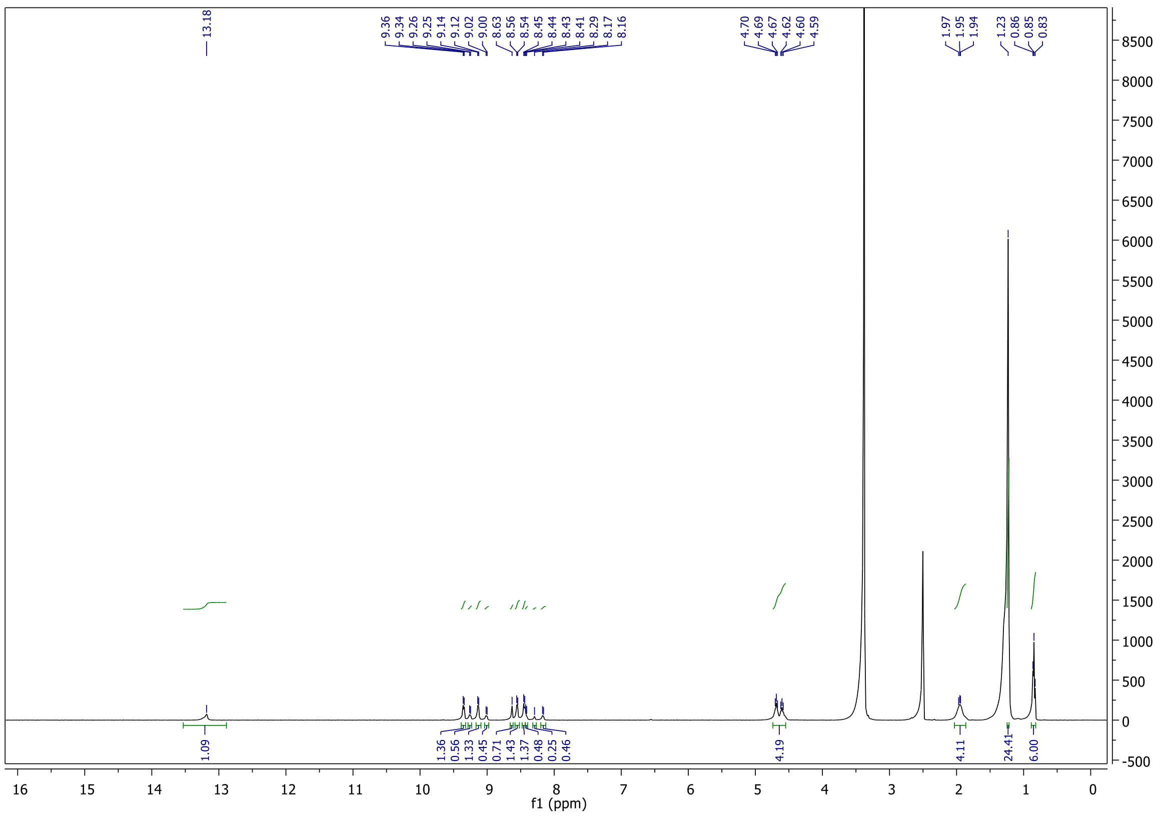

Figure S3: ^{1}H NMR of Compound **10**

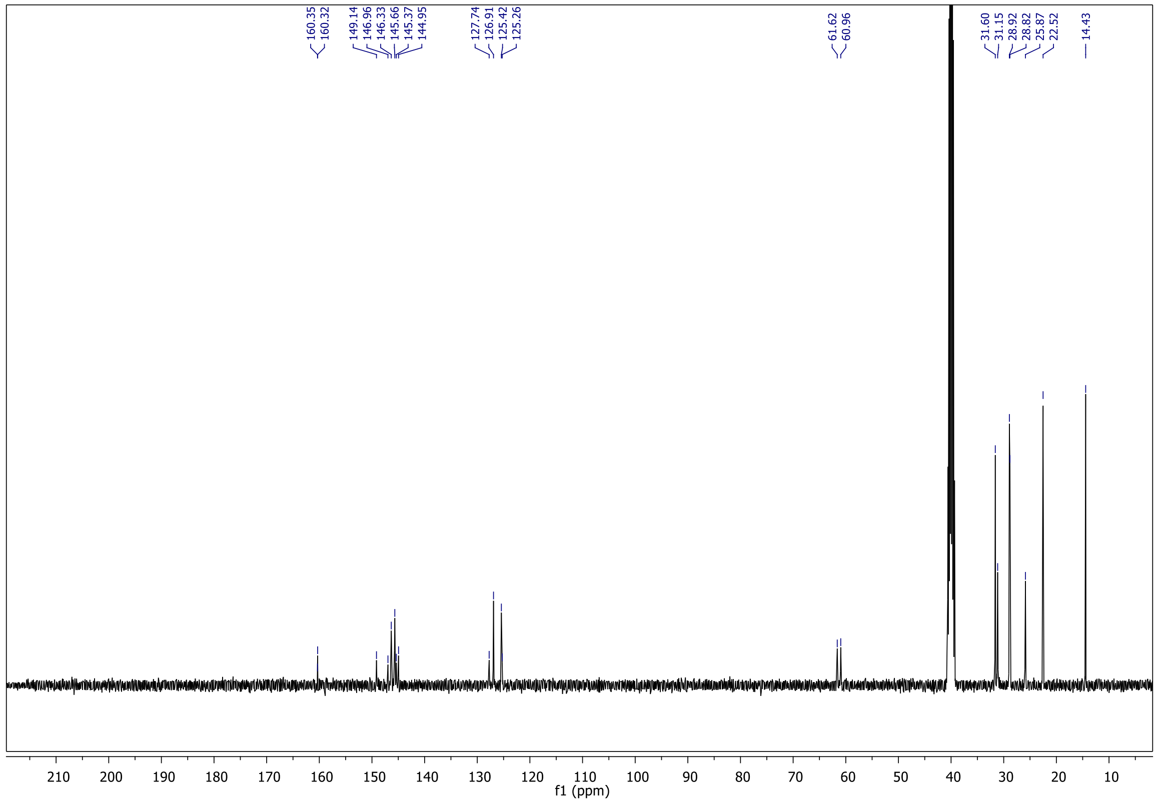

Figure S4: ^{13}C NMR of Compound **10**

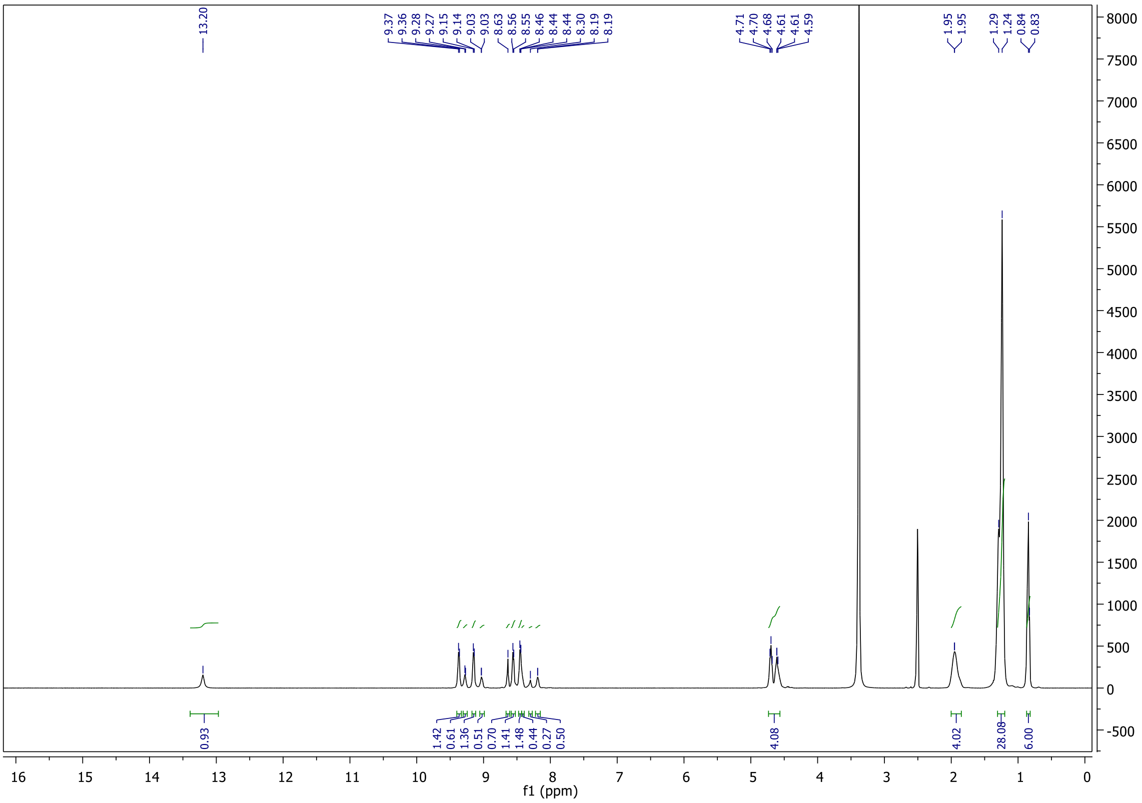

Figure S5: ^{1}H NMR of Compound **11**

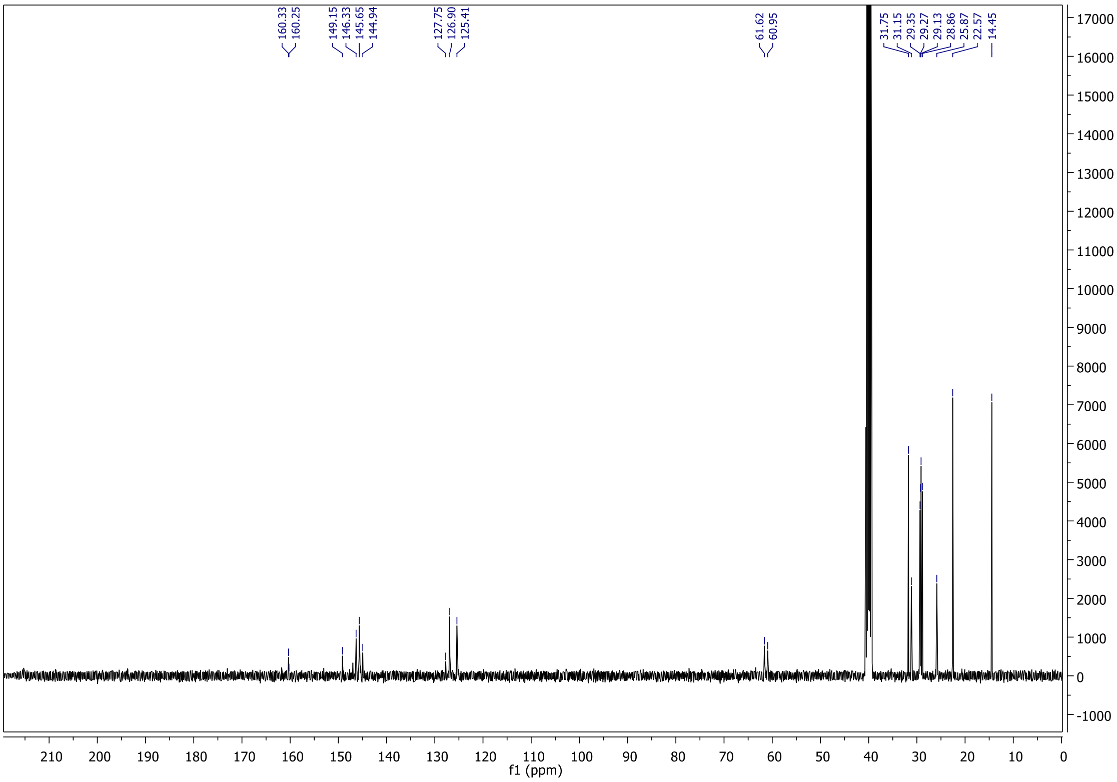

Figure S6: ^{13}C NMR of Compound **11**

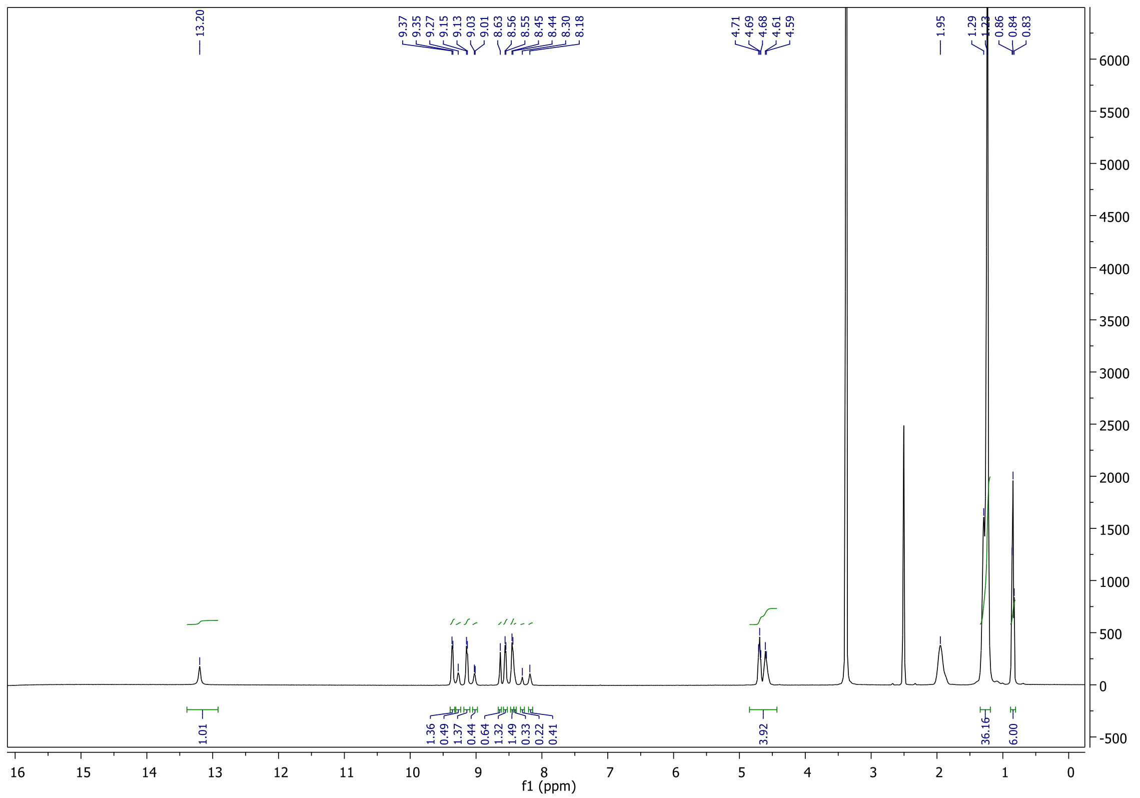

Figure S7: ^{1}H NMR of Compound **12**

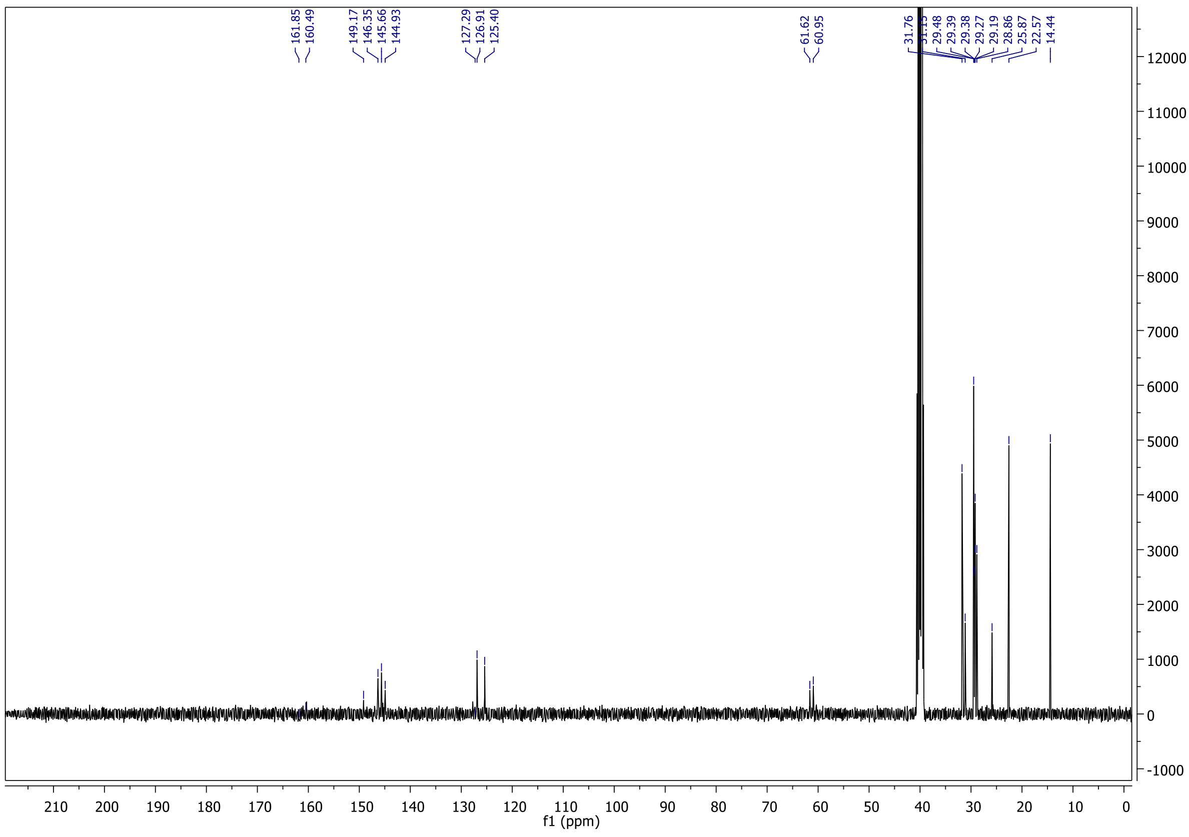

Figure S8: ^{13}C NMR of Compound **12**

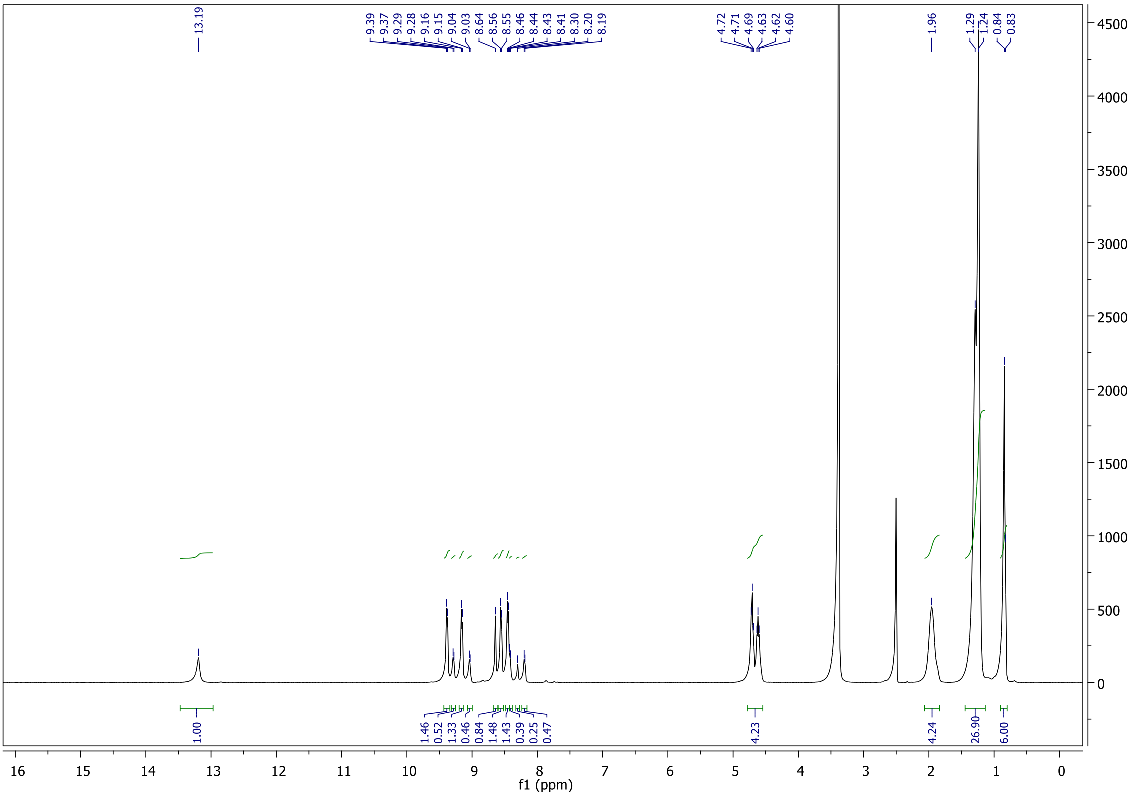

Figure S9: ^{1}H NMR of Compound **13**

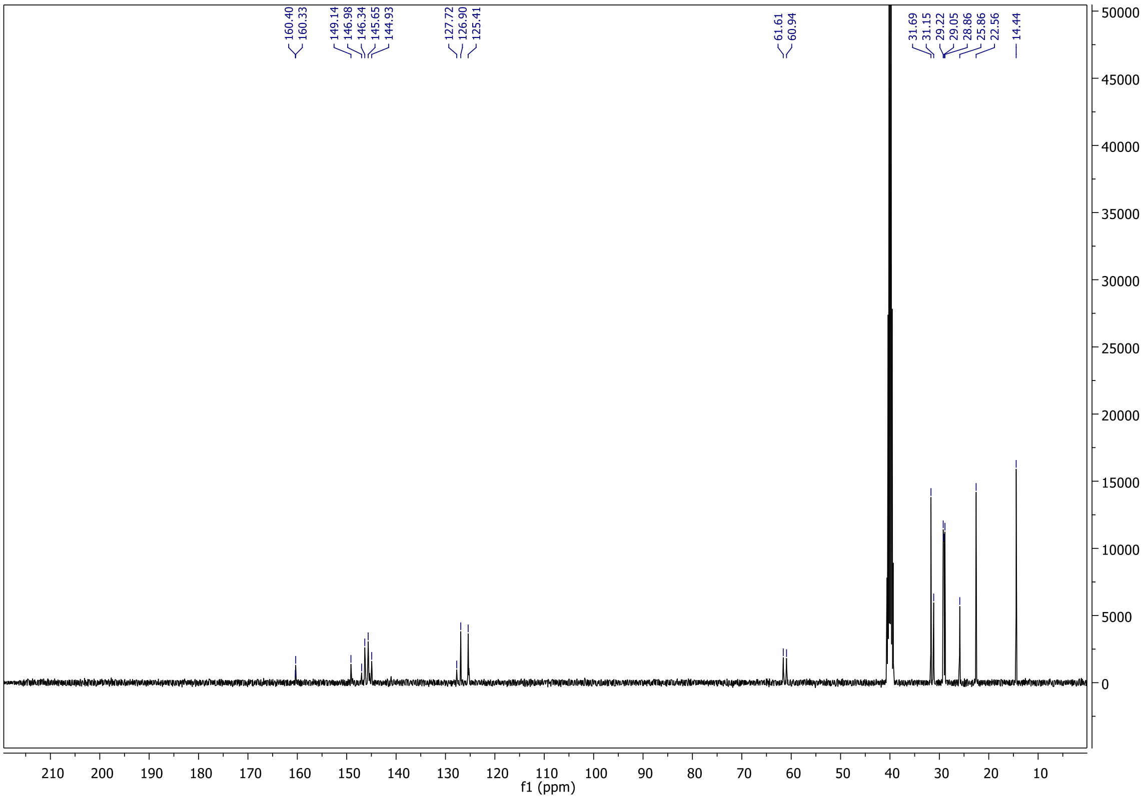

Figure S10: ^{13}C NMR of Compound **13**

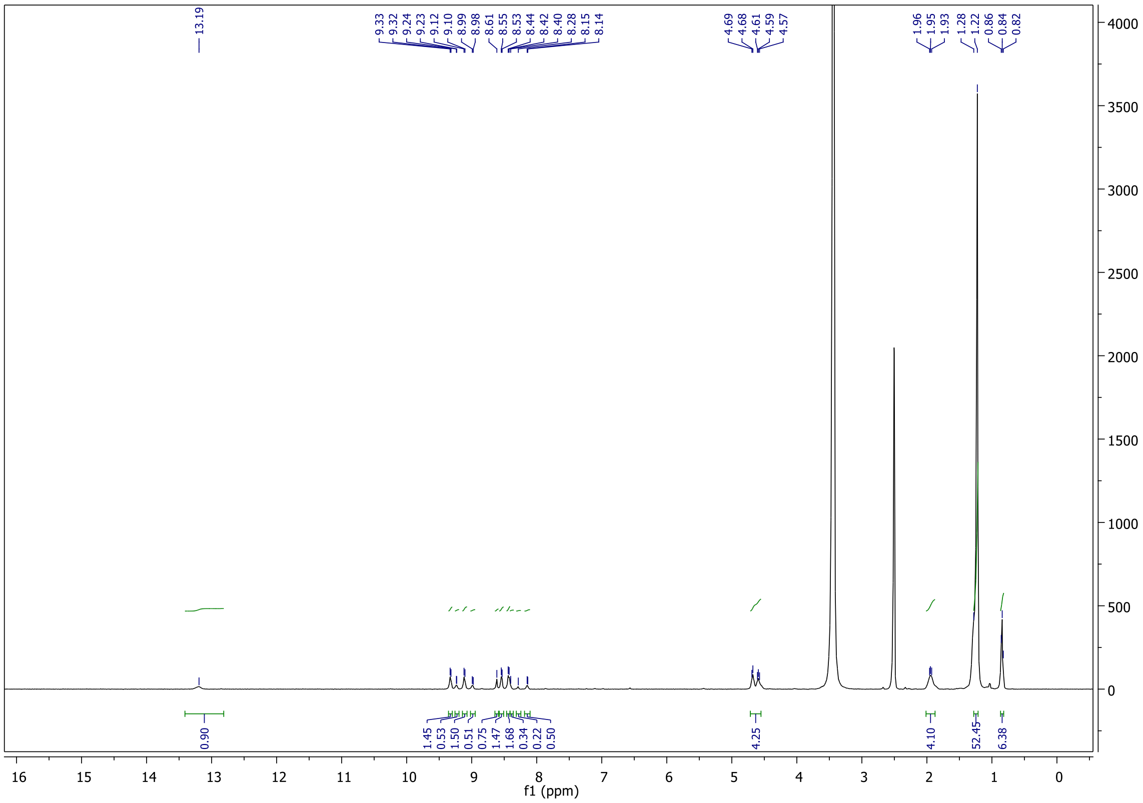

Figure S11: ^{1}H NMR of Compound **14**

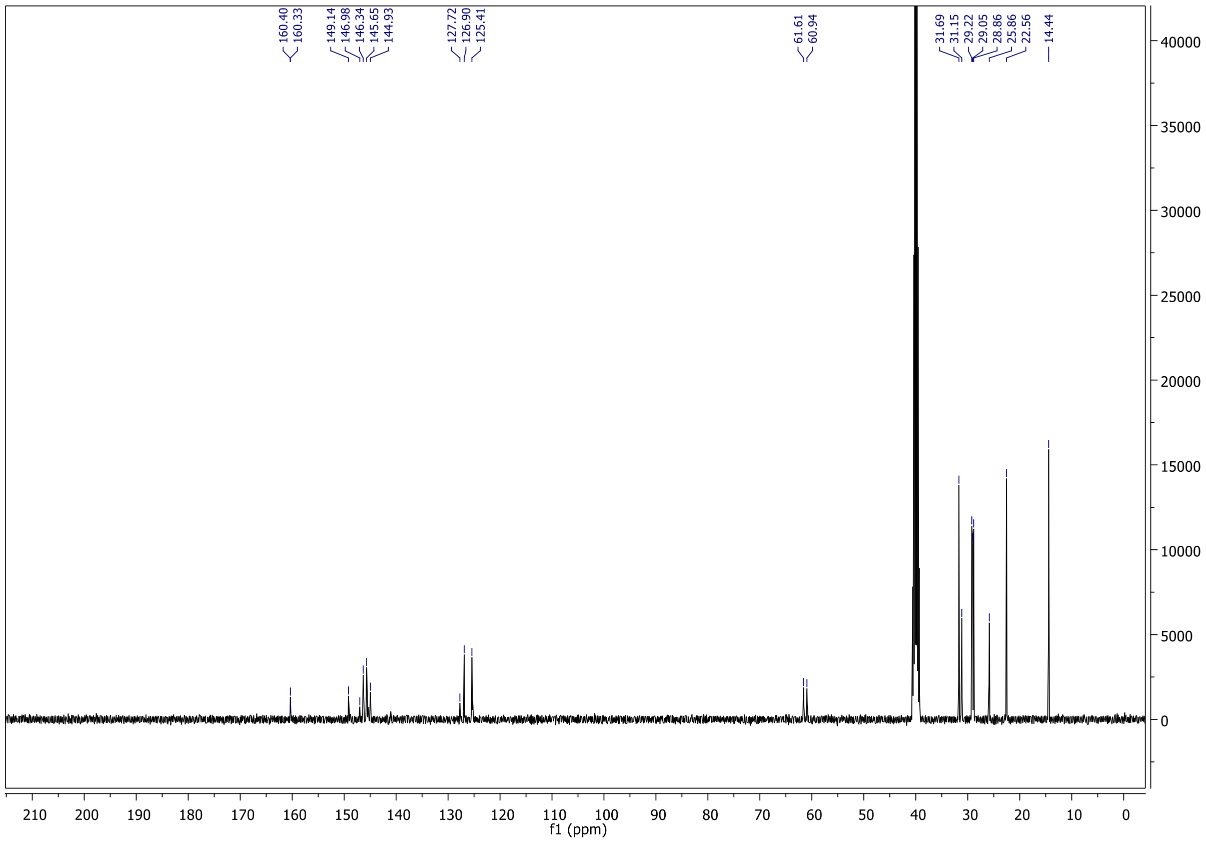

Figure S12: ^{13}C NMR of Compound **14**

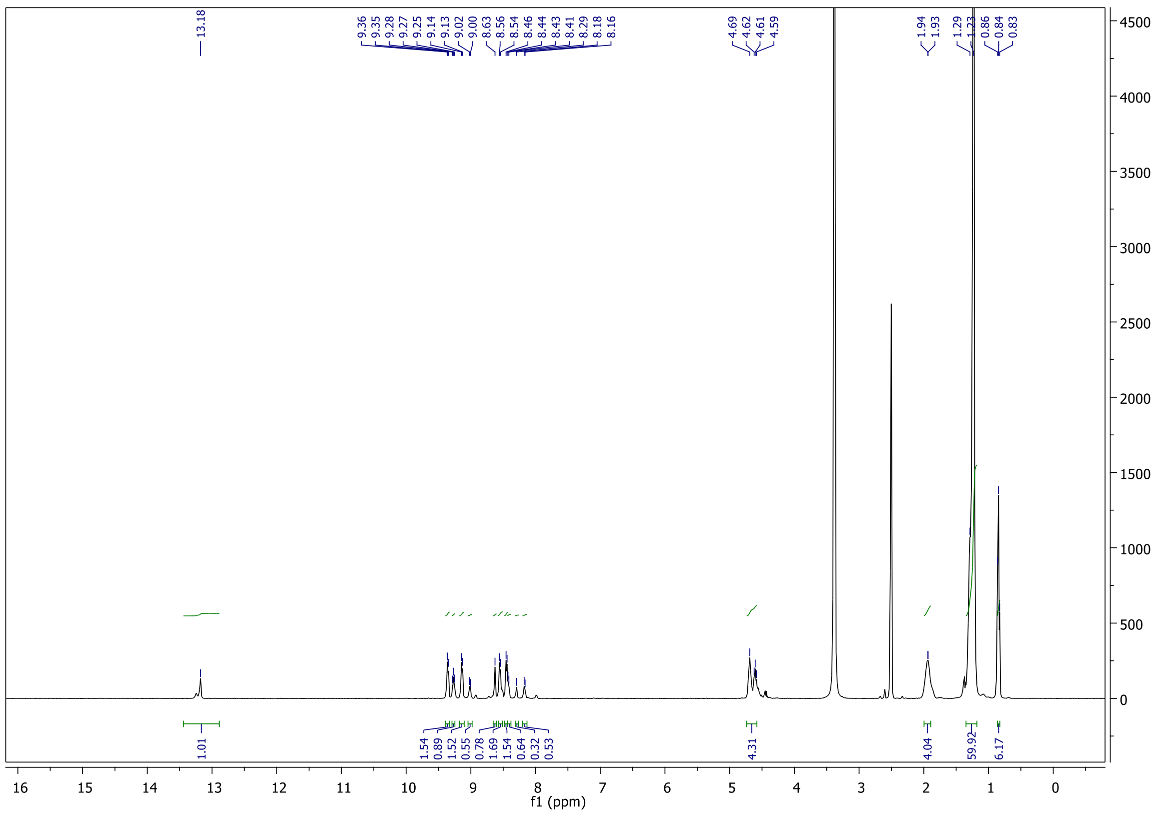

Figure S13: ^{1}H NMR of Compound **15**

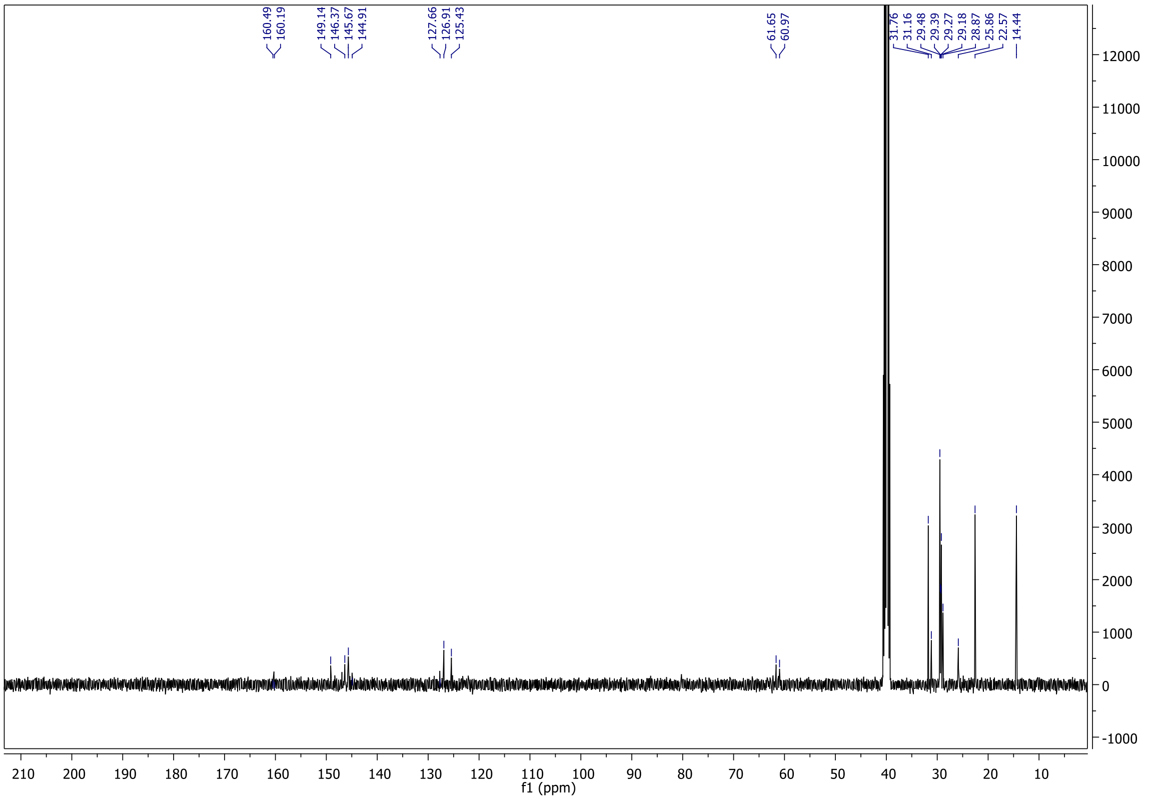

Figure S14: ^{13}C NMR of Compound **15**

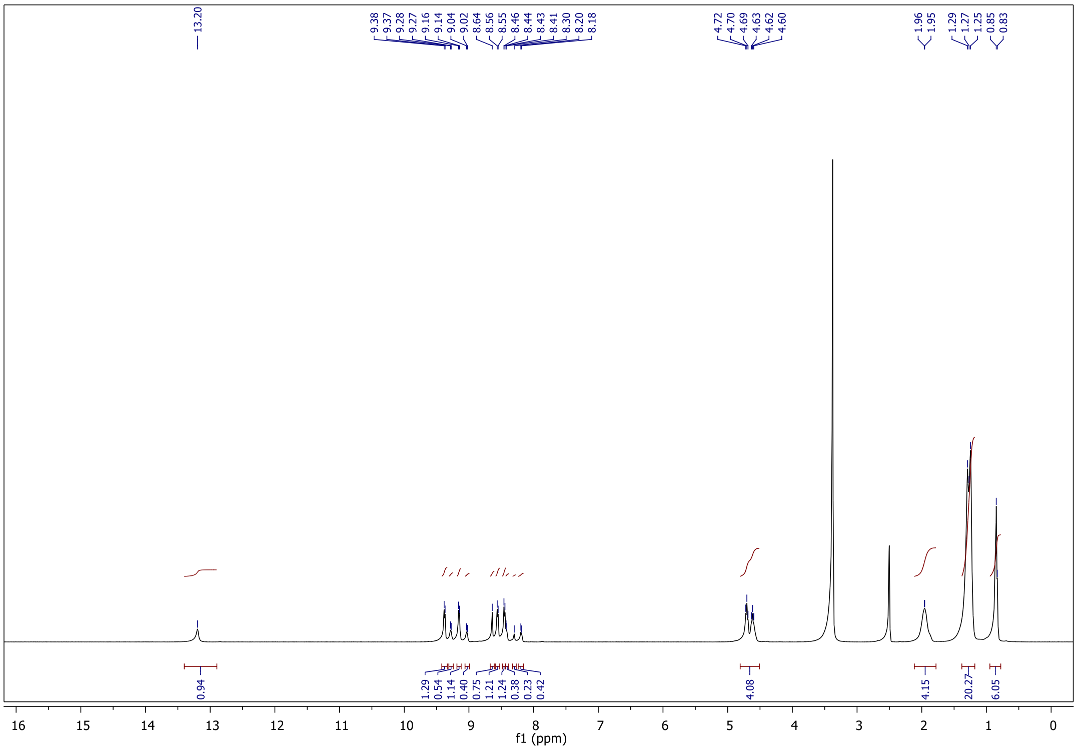

Figure S15: ^{1}H NMR of Compound **16**

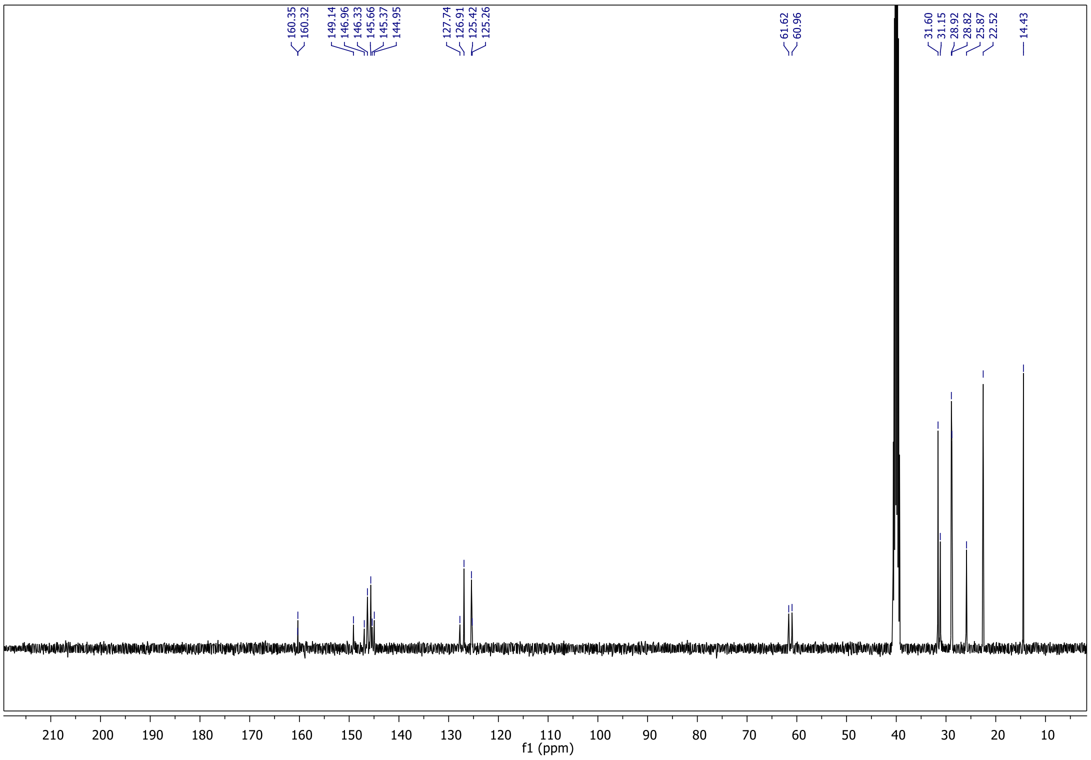

Figure S16: ^{13}C NMR of Compound **16**

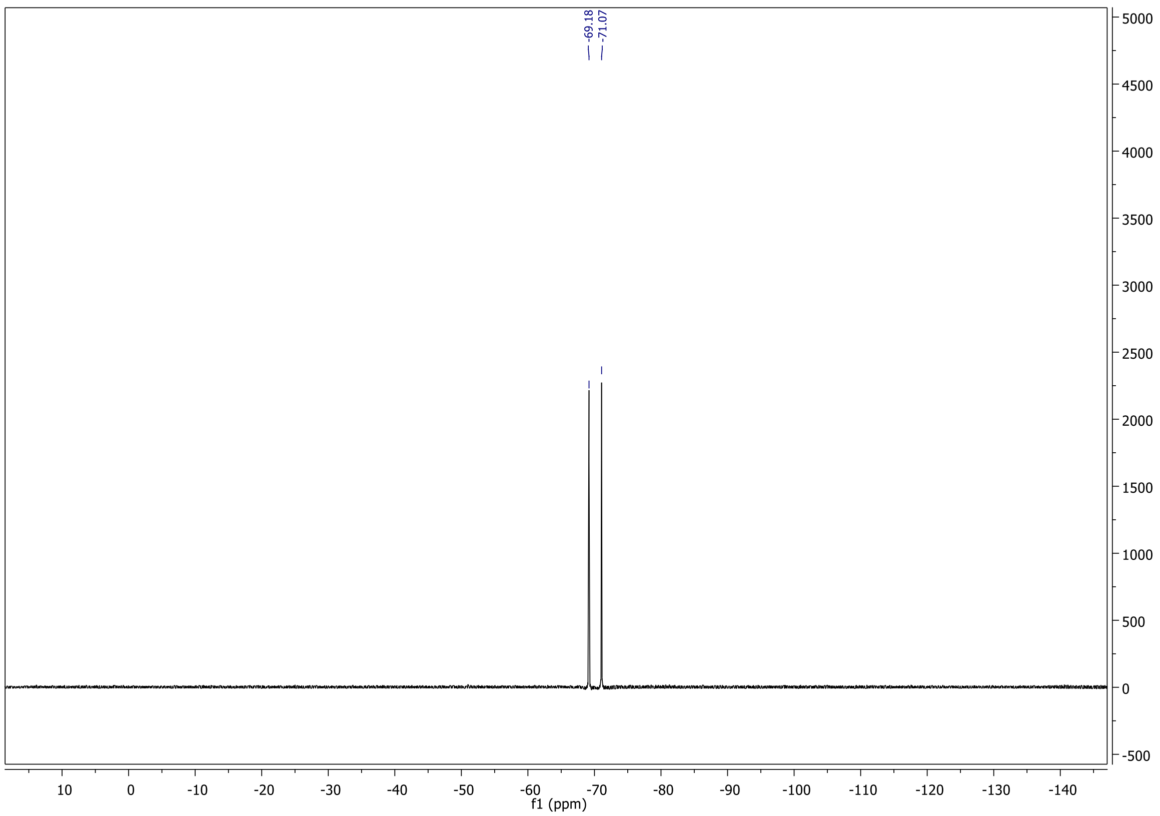

Figure S17: ^{19}F NMR of Compound **16**

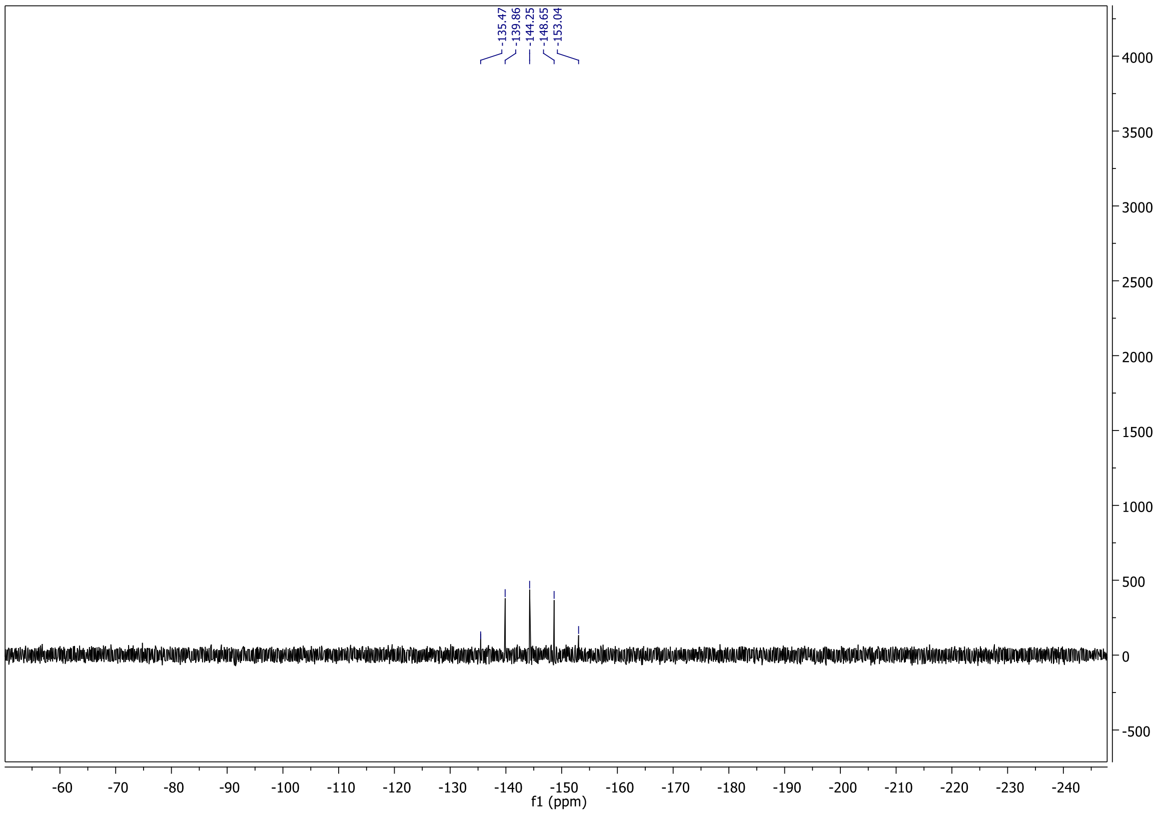

Figure S18: ^{31}P NMR of Compound **16**

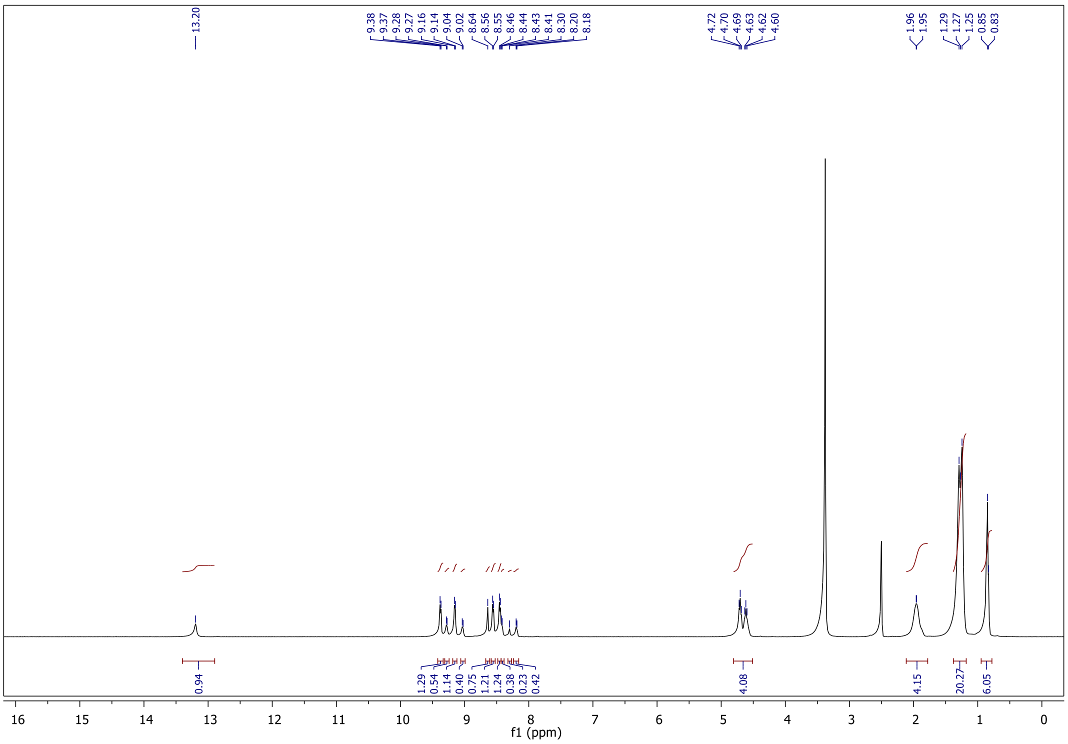

Figure S19: ^{1}H NMR of Compound **17**

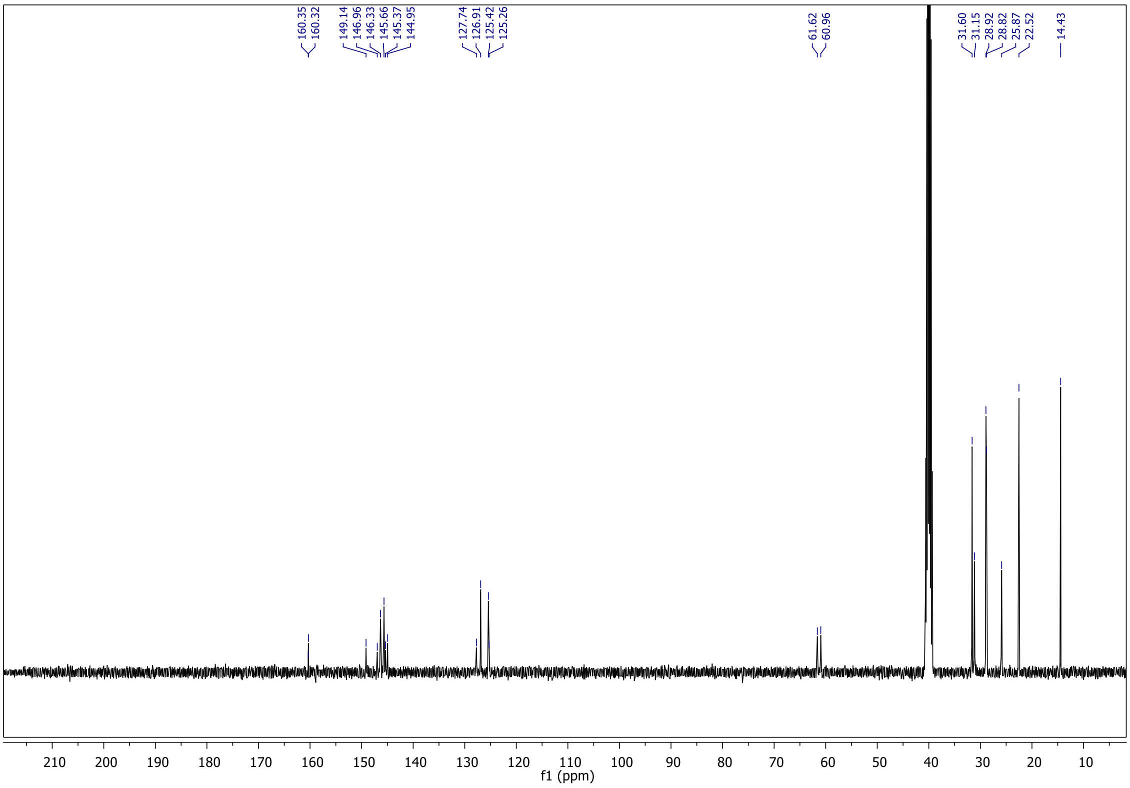

Figure S20: ^{13}C NMR of Compound **17**

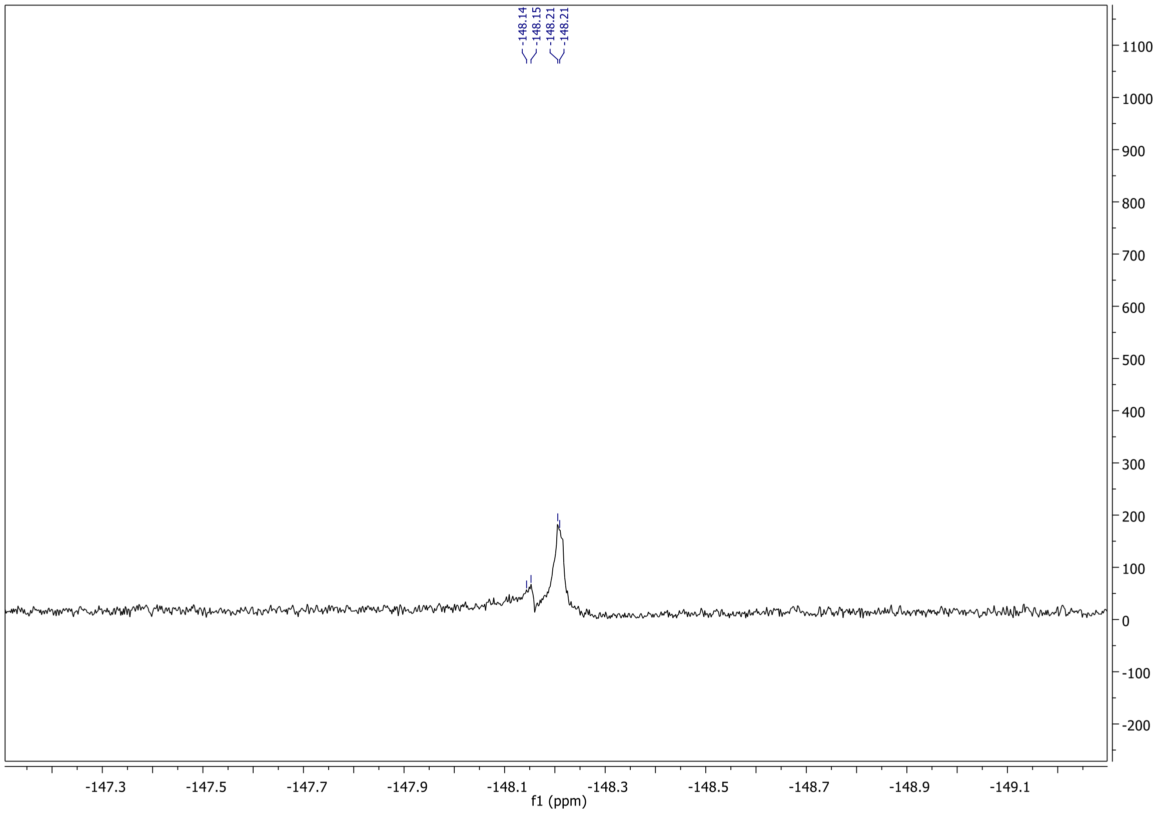

Figure S21: ^{19}F NMR of Compound **17**

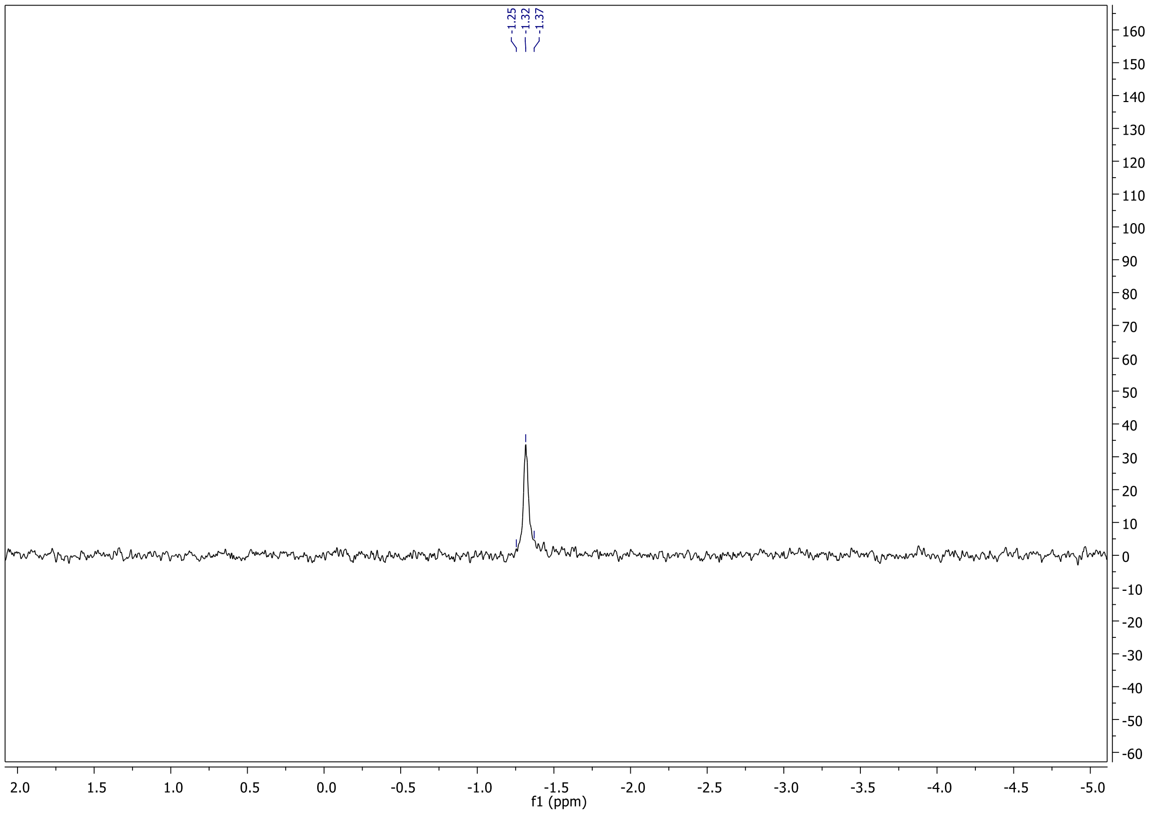

Figure S22: ^{11}B NMR of Compound **17**

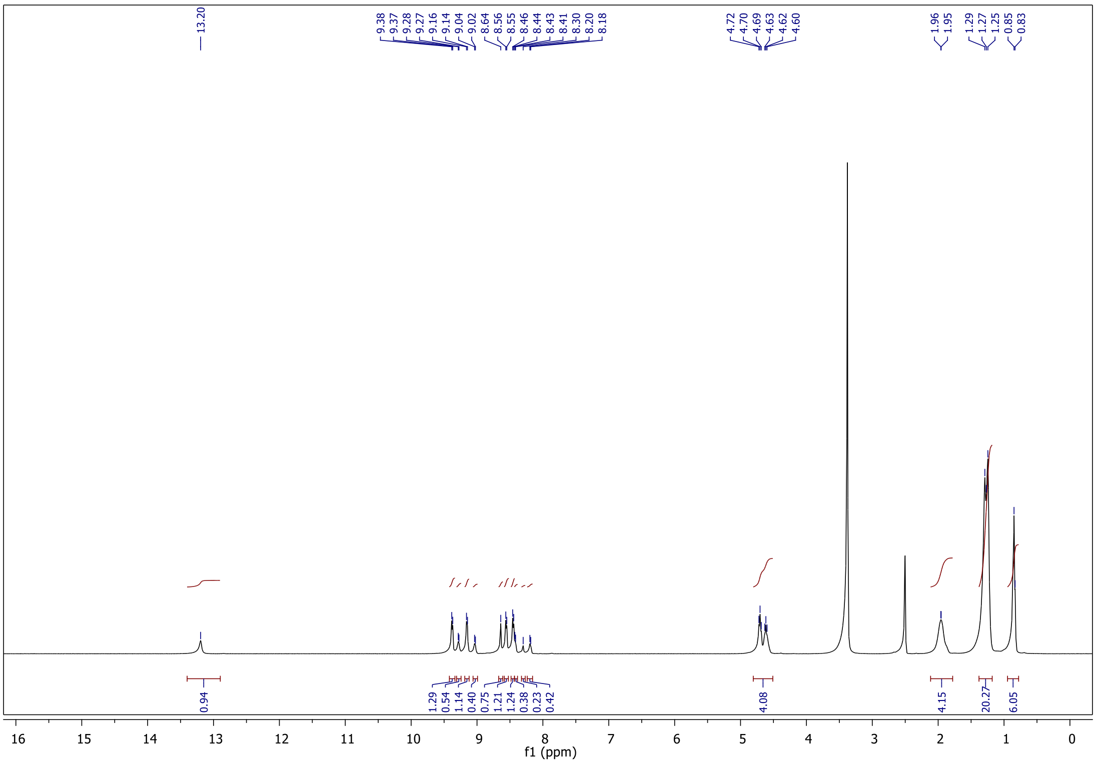

Figure S23: ^{1}H NMR of Compound **18**

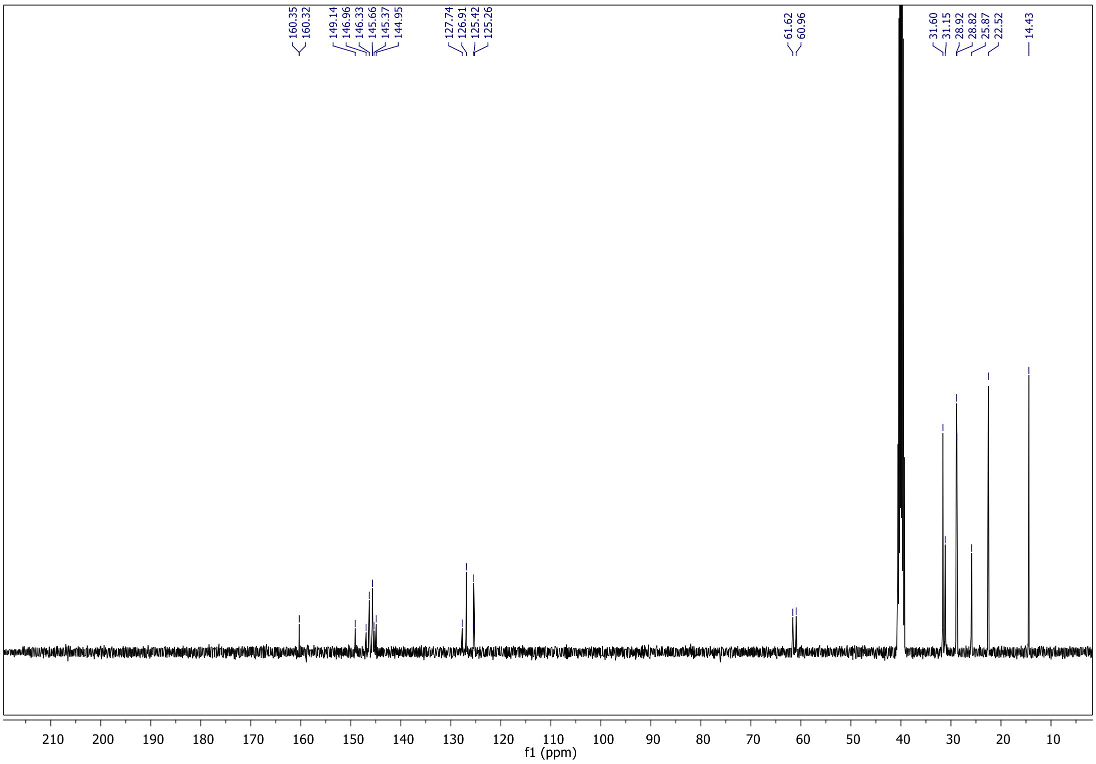

Figure S24: ^{13}C NMR of Compound **18**

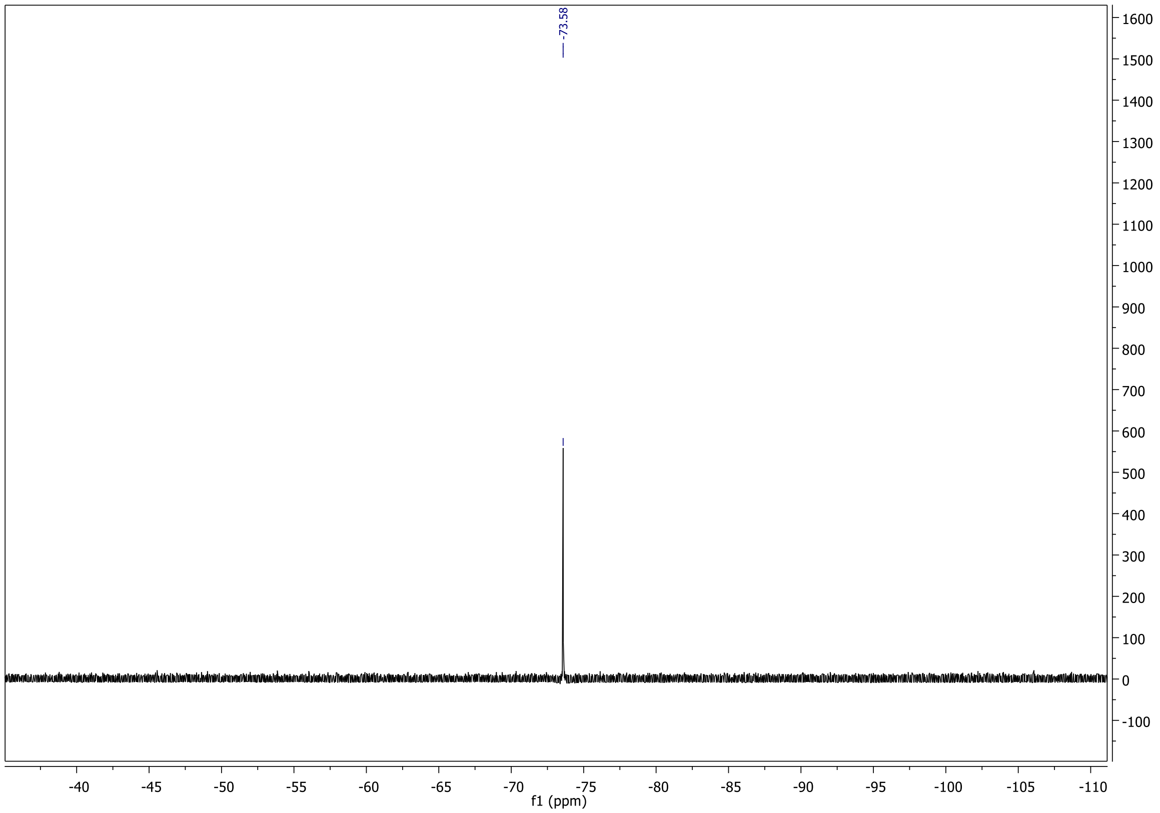

Figure S25: ^{19}F NMR of Compound **18**

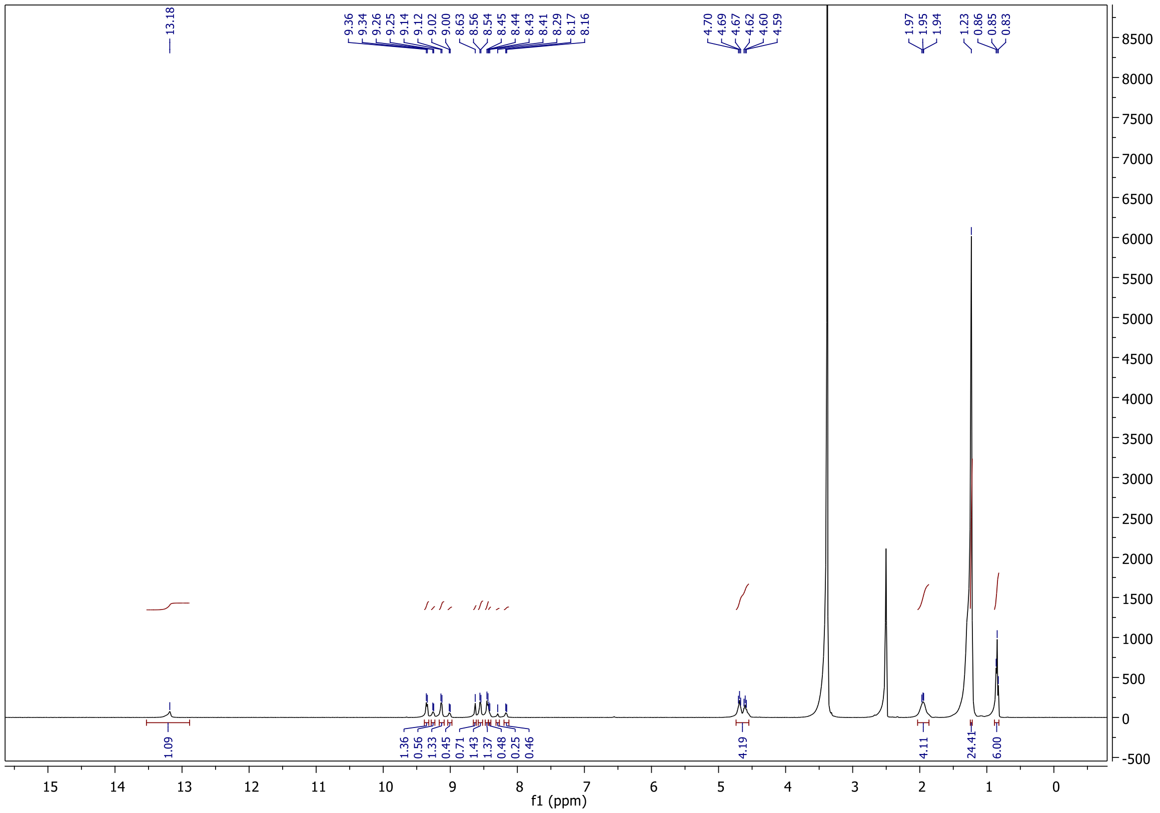

Figure S26: ^{1}H NMR of Compound **19**

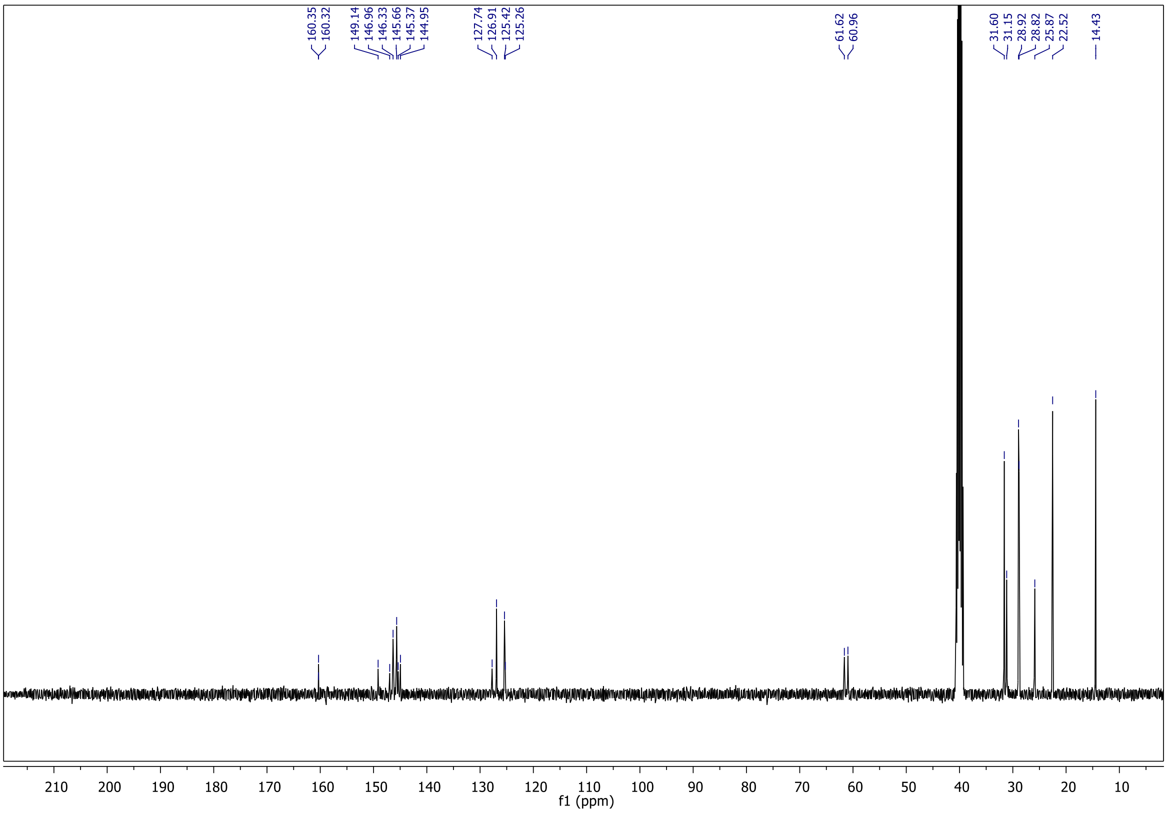

Figure S27: ^{13}C NMR of Compound **19**

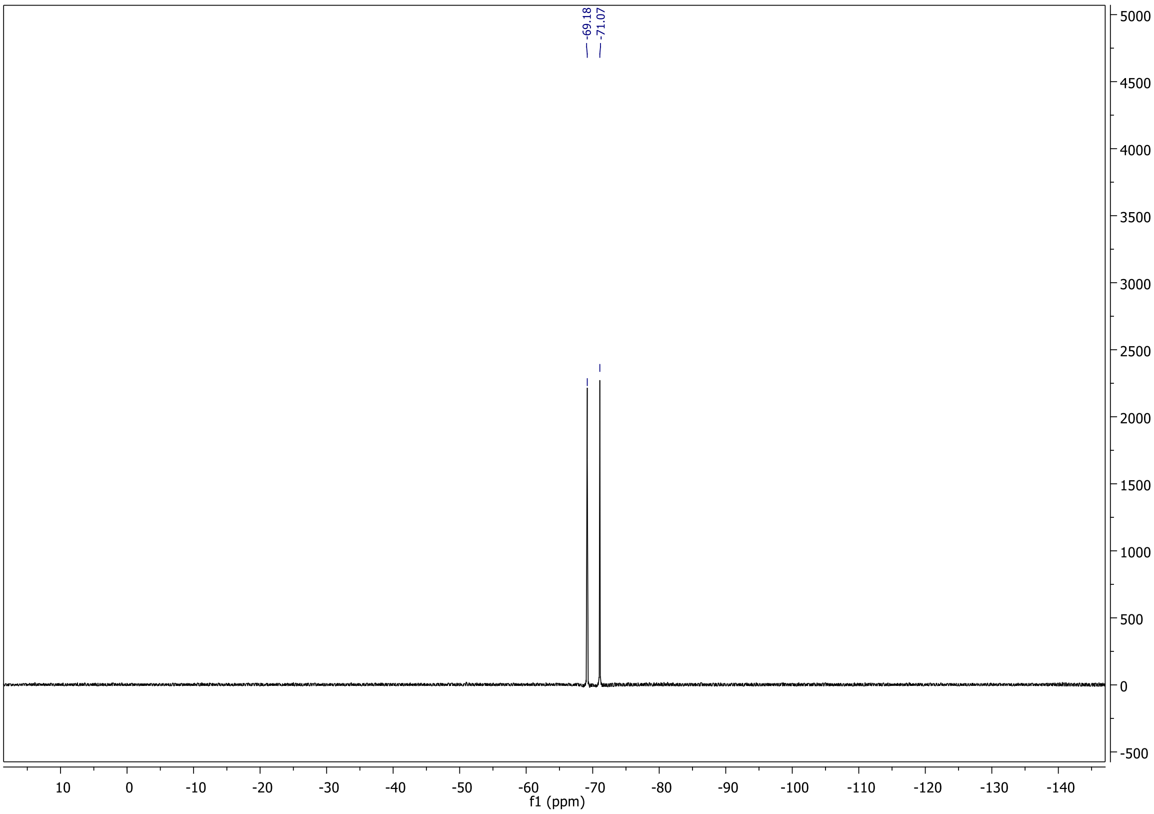

Figure S28: ^{19}F NMR of Compound **19**

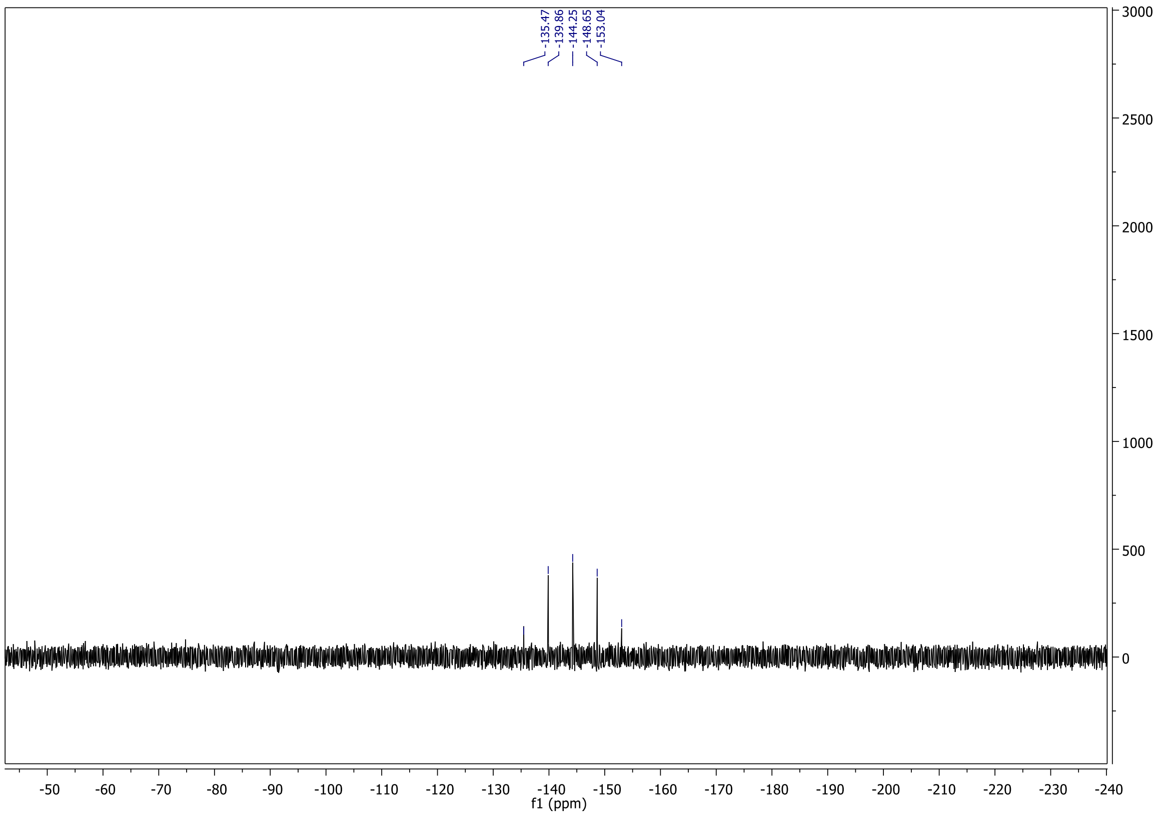

Figure S29: ^{31}P NMR of Compound **19**

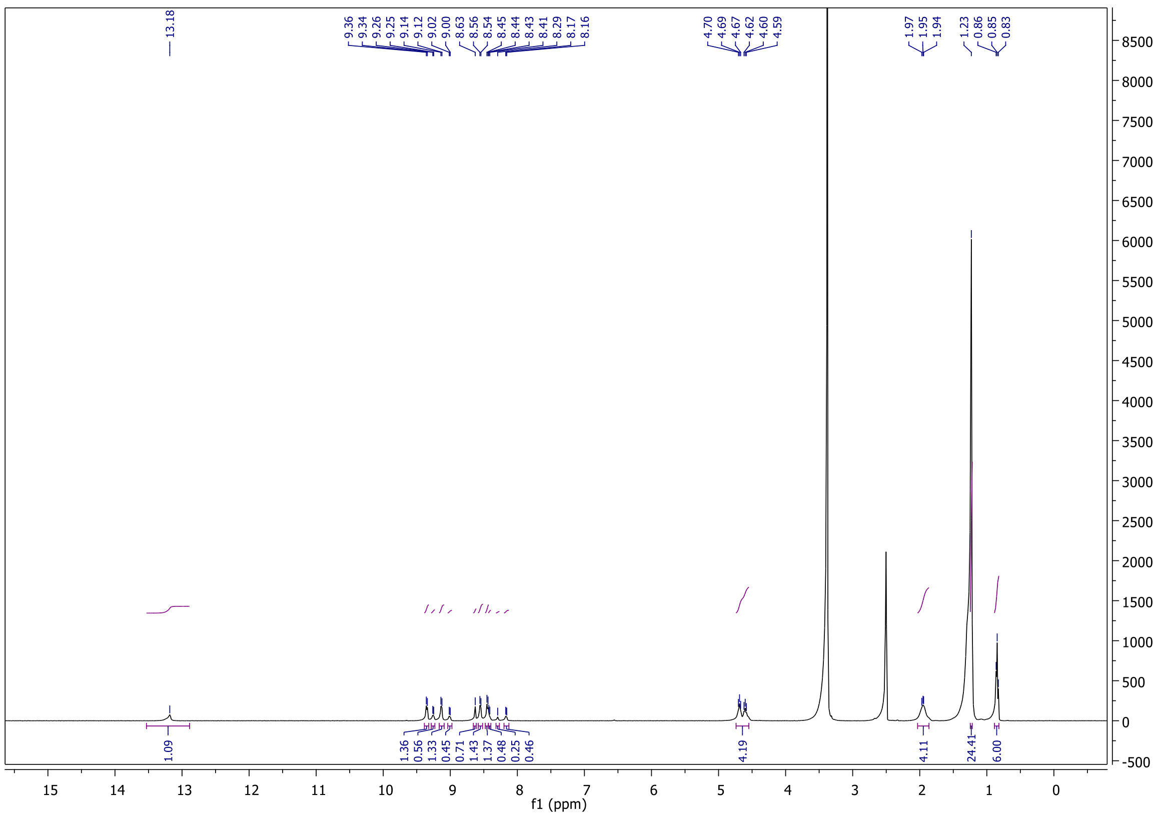

Figure S30: ^{1}H NMR of Compound **20**

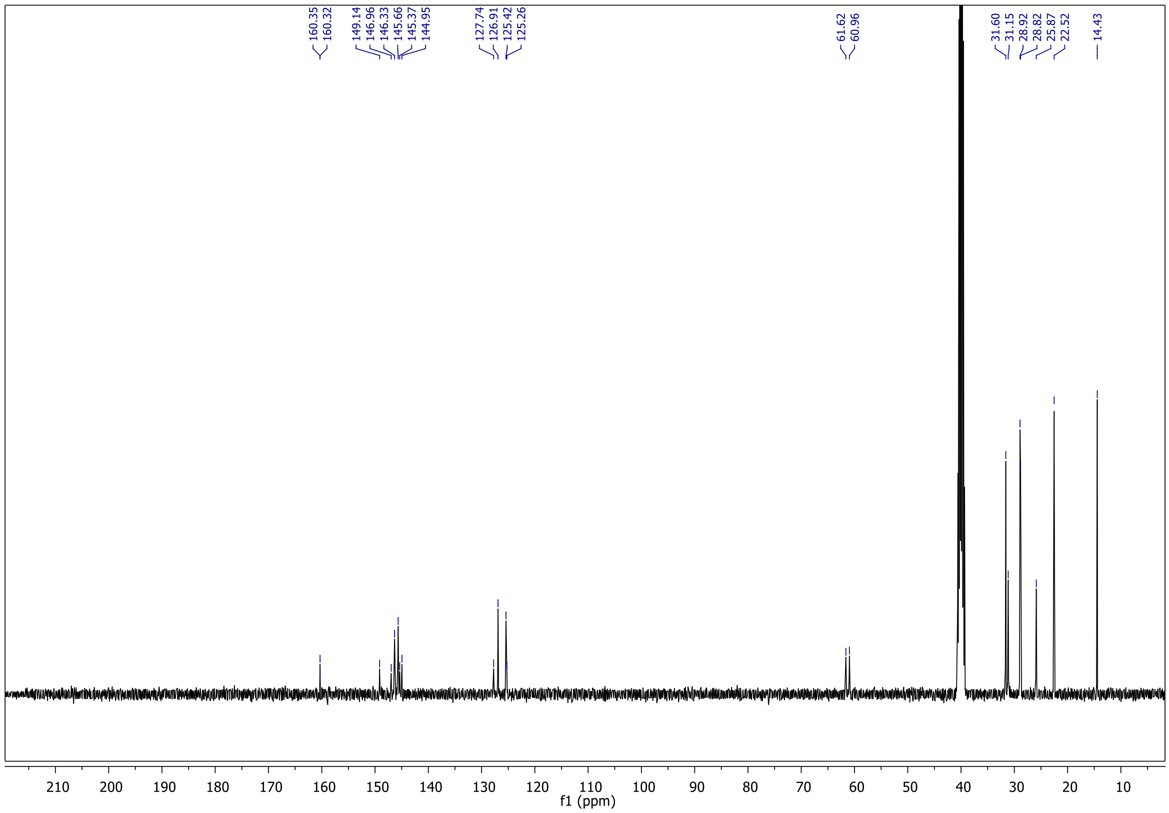

Figure S31: ^{13}C NMR of Compound **20**

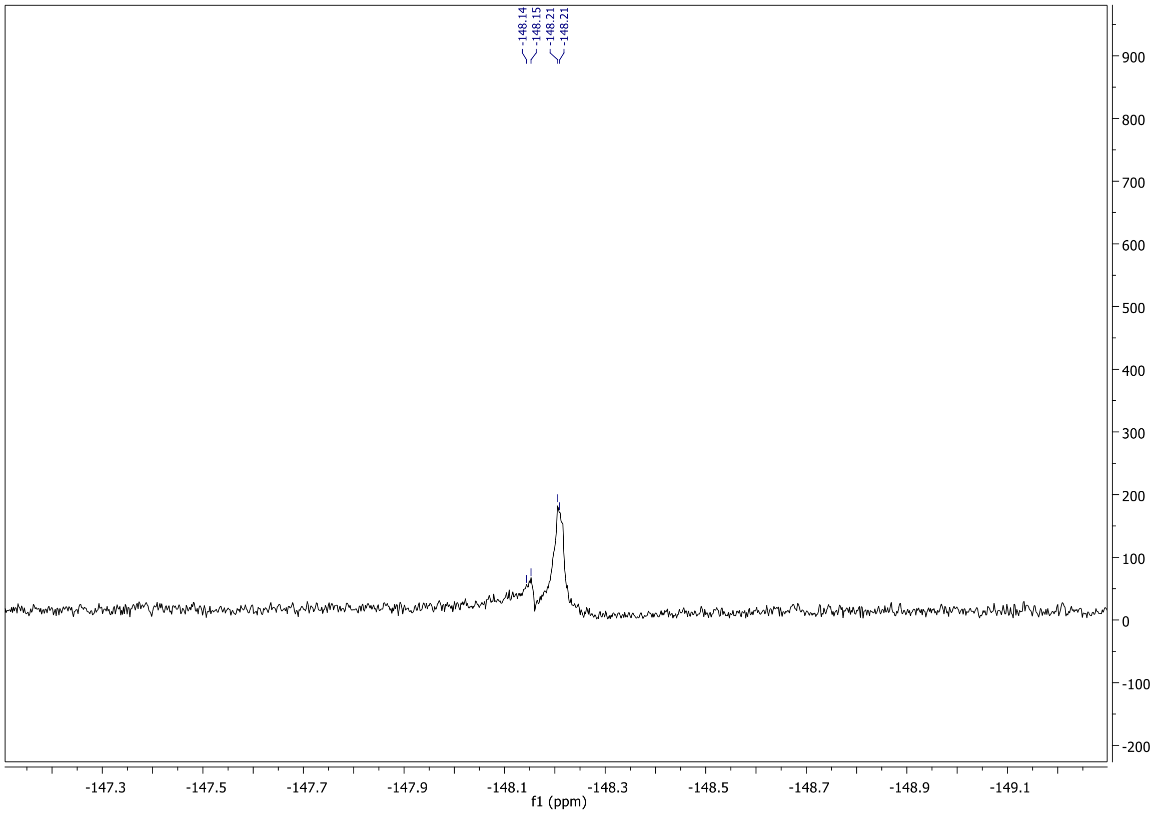

Figure S32: ^{19}F NMR of Compound **20**

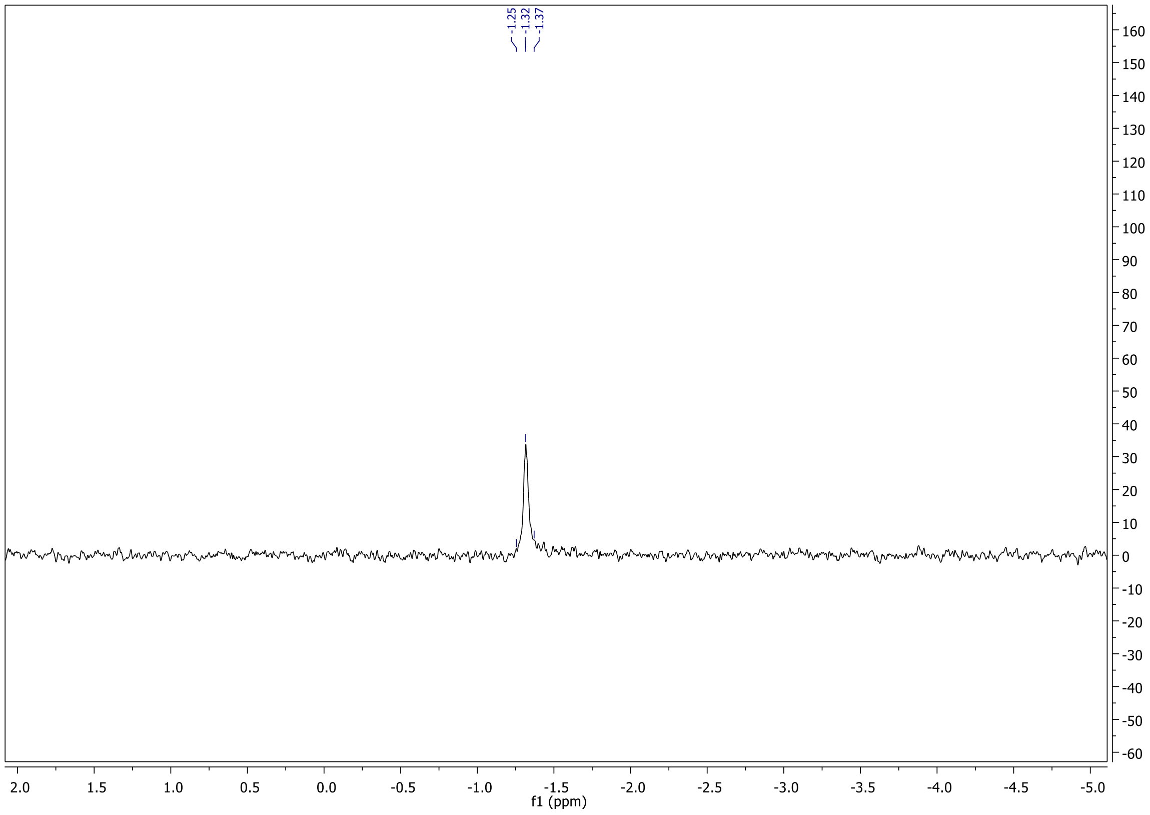

Figure S33: ^{11}B NMR of Compound **20**

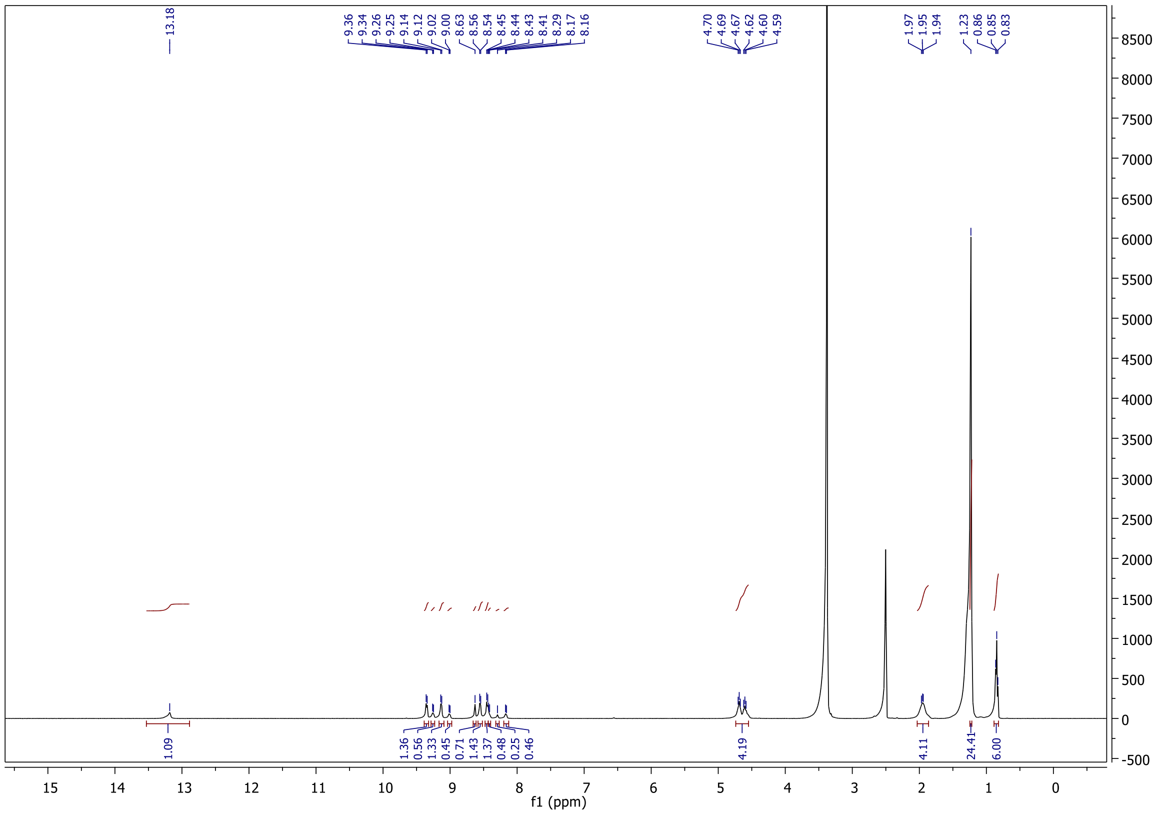

Figure S34: ^{1}H NMR of Compound **21**

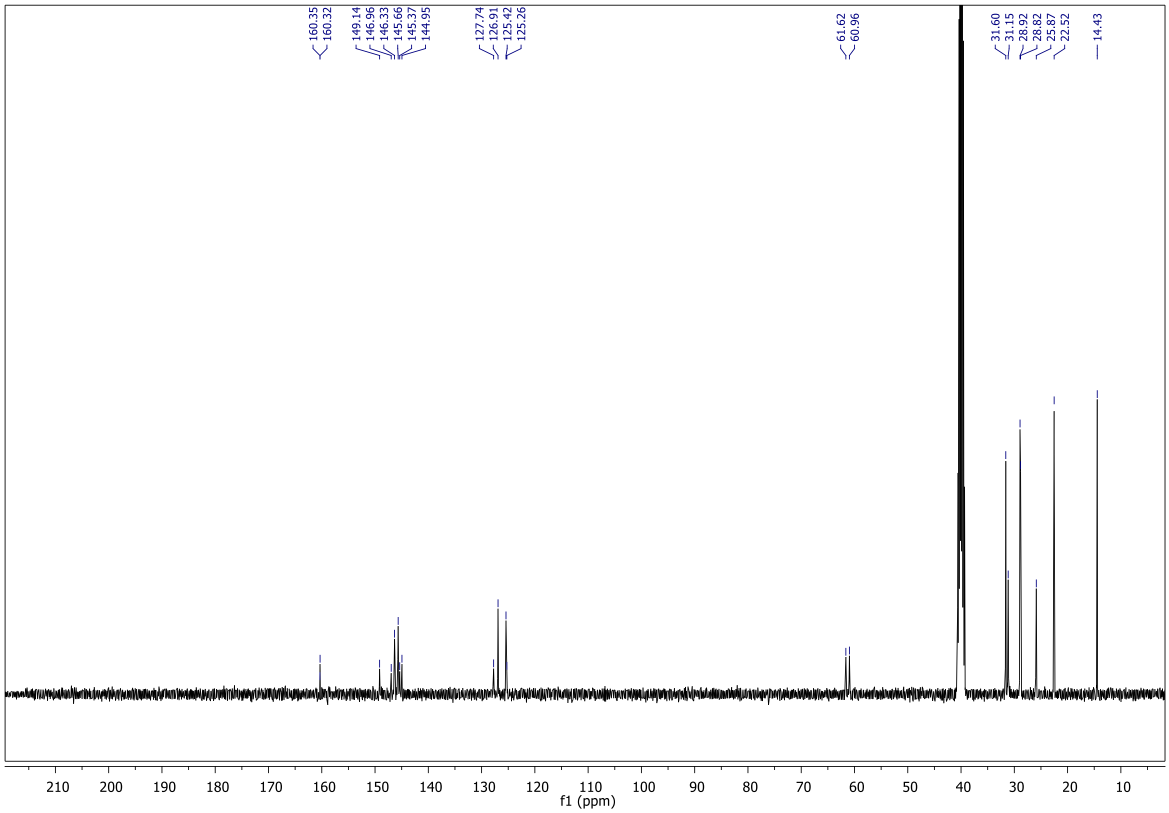

Figure S35: ^{13}C NMR of Compound **21**

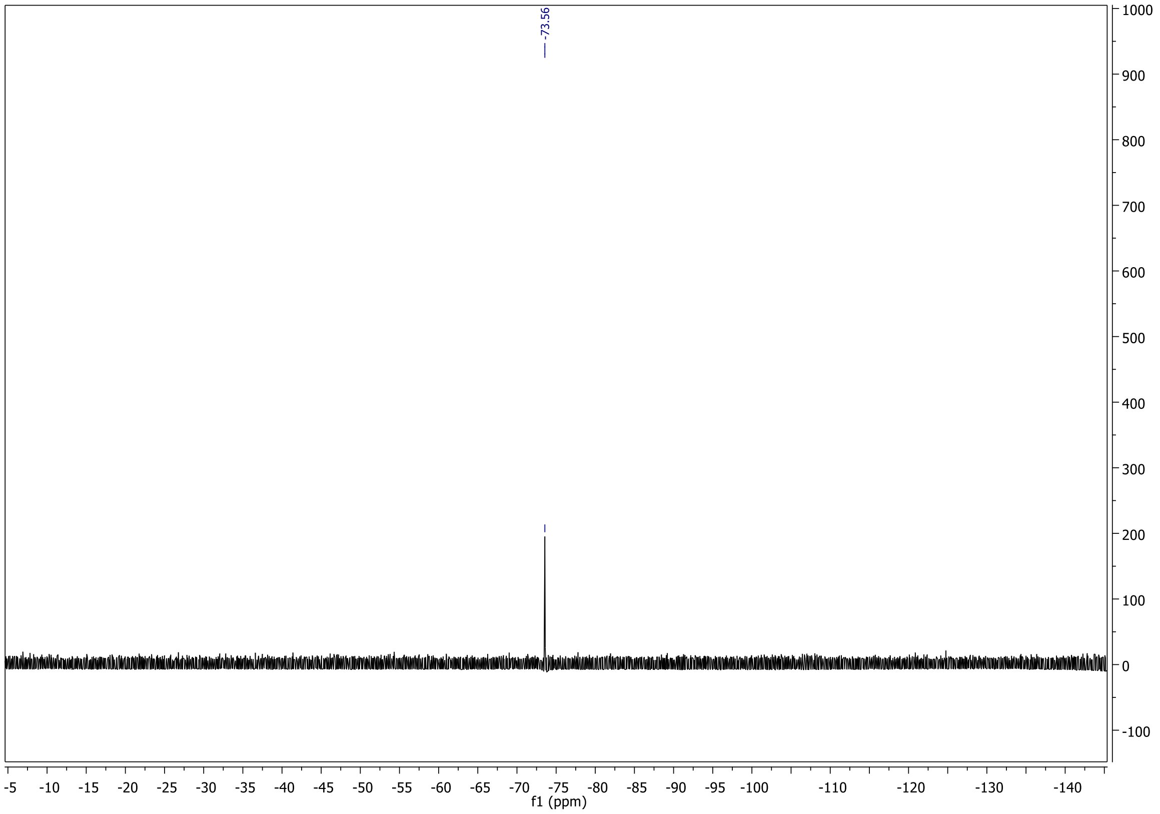

Figure S36: ^{19}F NMR of Compound **21**

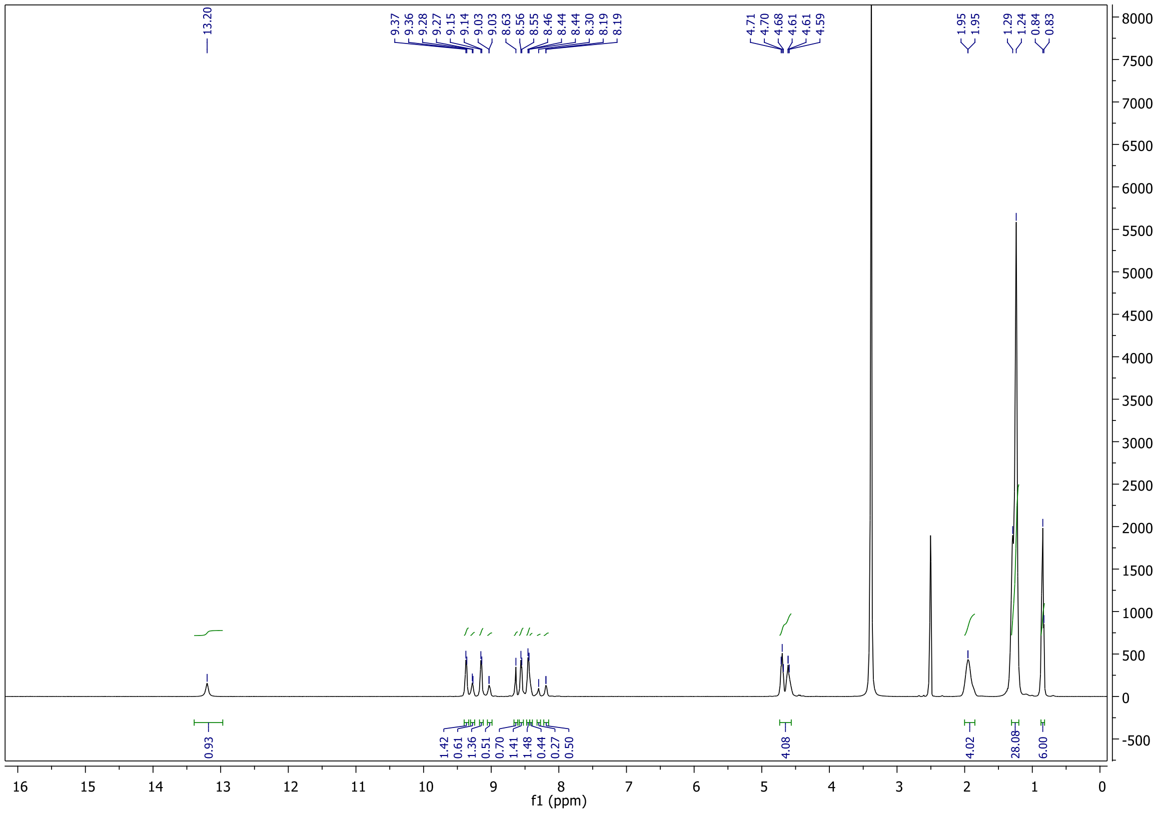

Figure S37: ^{1}H NMR of Compound **22**

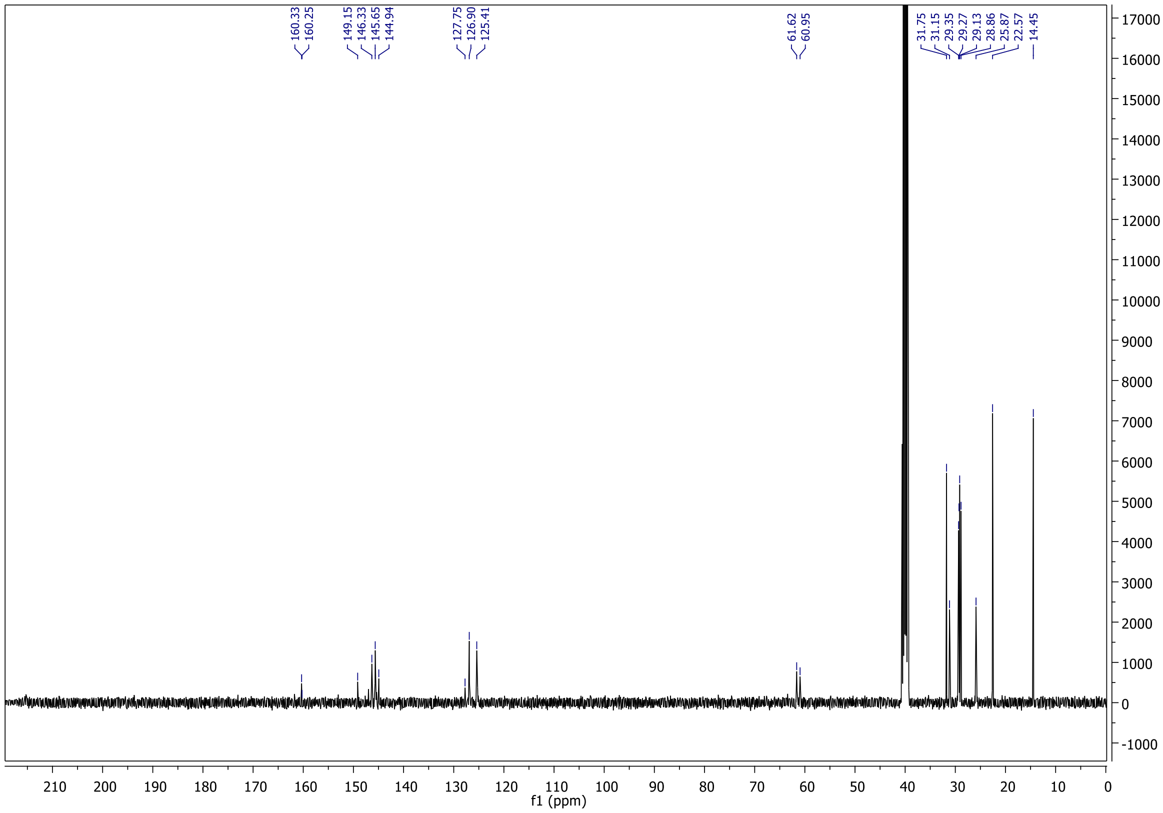

Figure S38: ^{13}C NMR of Compound **22**

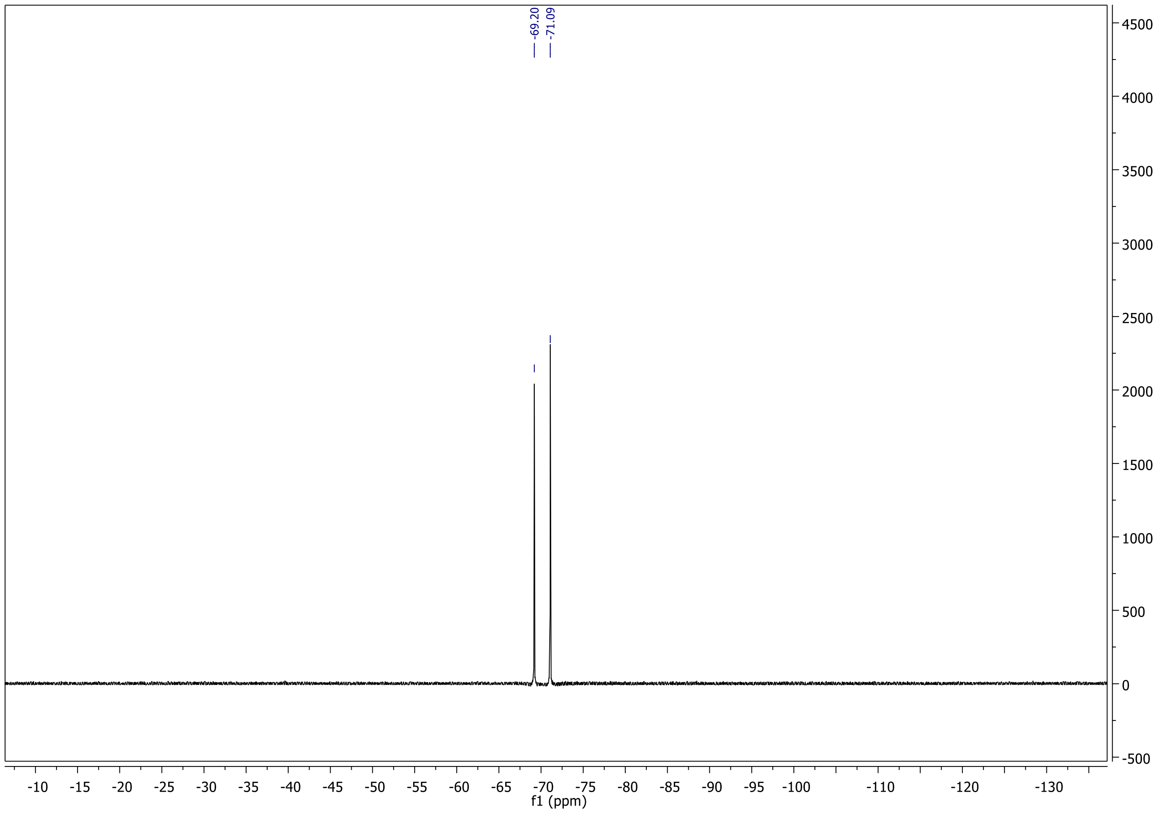

Figure S39: ^{19}F NMR of Compound **22**

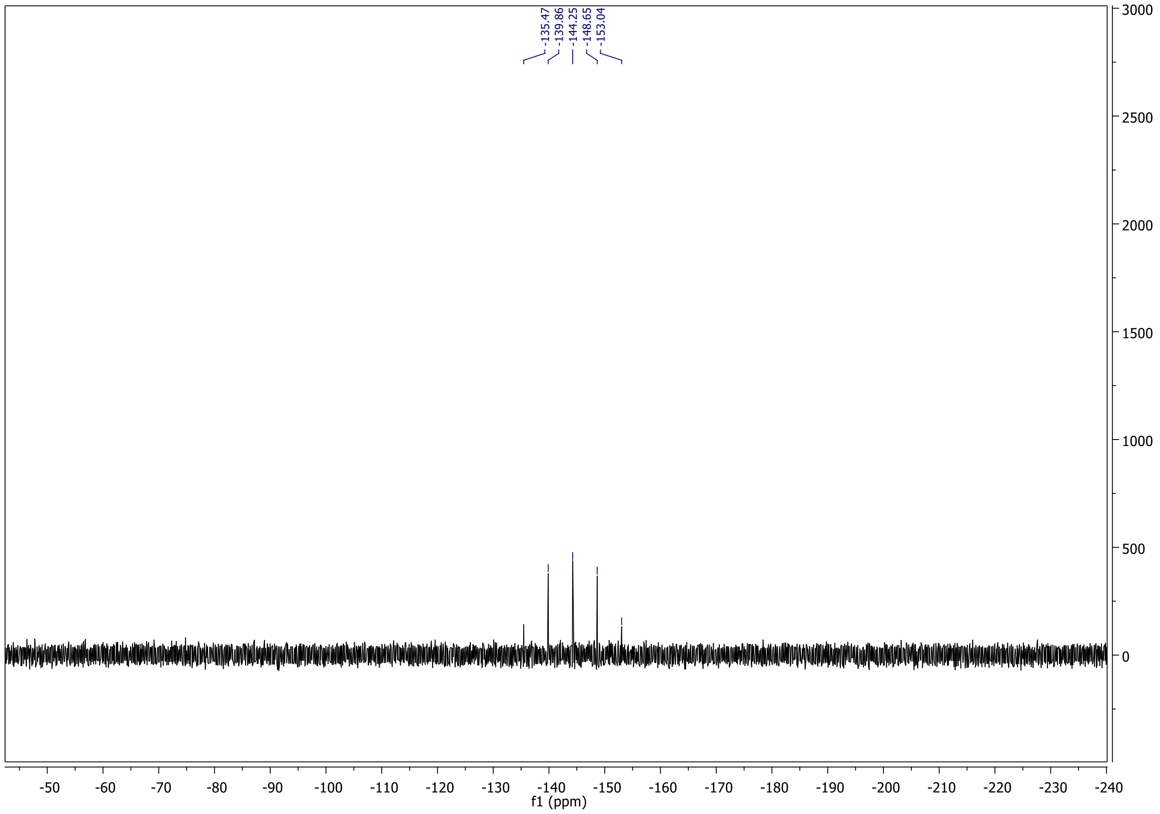

Figure S40: ^{31}P NMR of Compound **22**

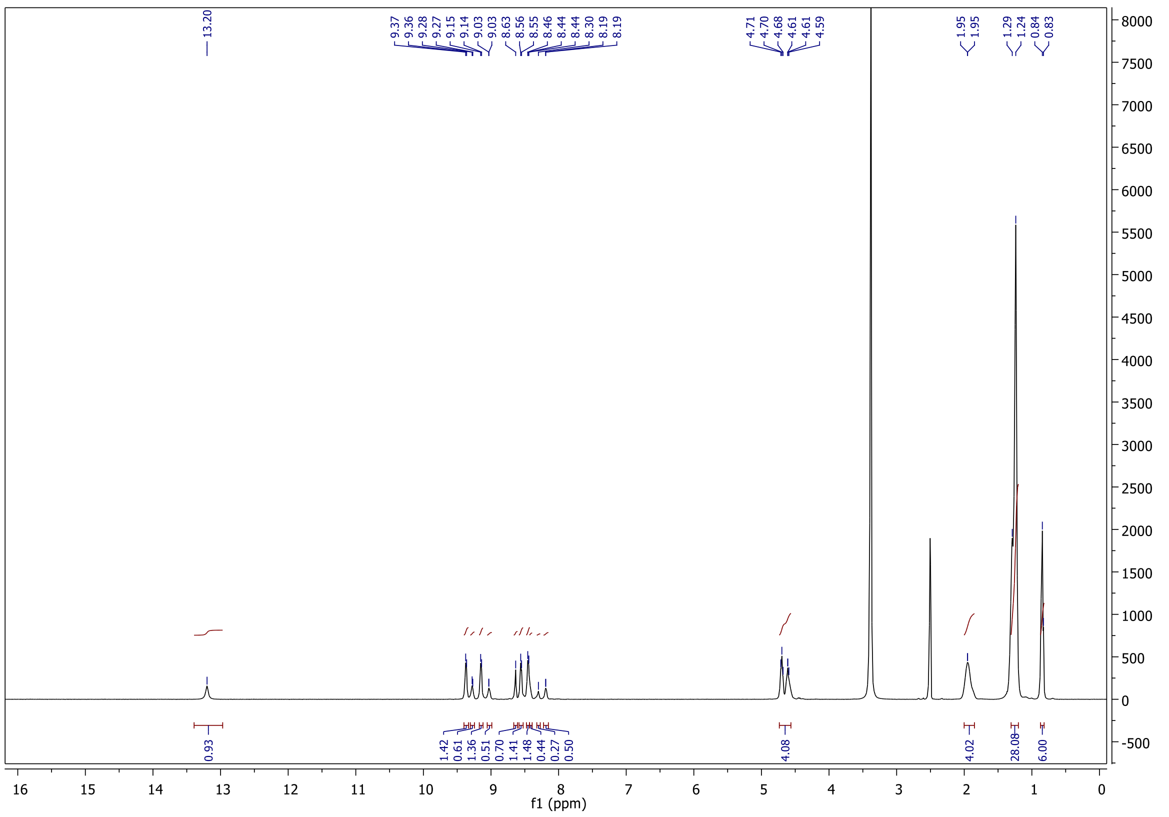

Figure S41: ^{1}H NMR of Compound **23**

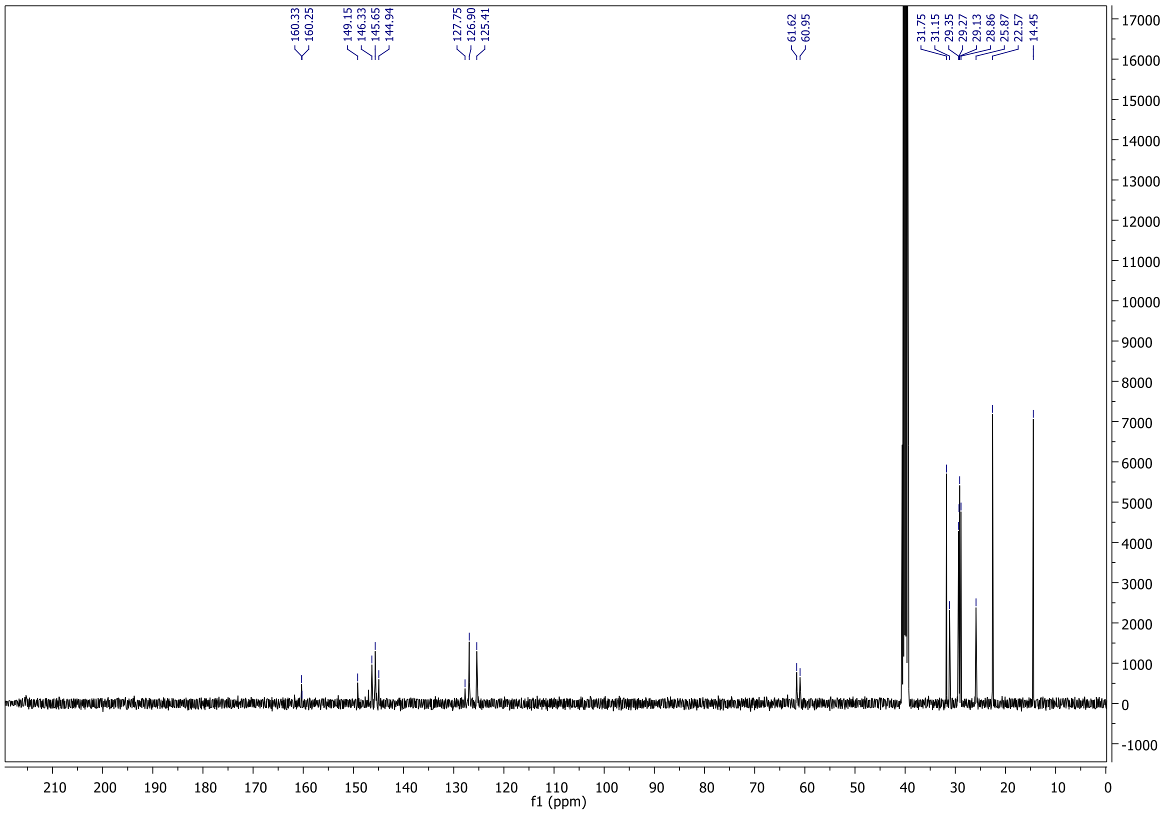

Figure S42: ^{13}C NMR of Compound **23**

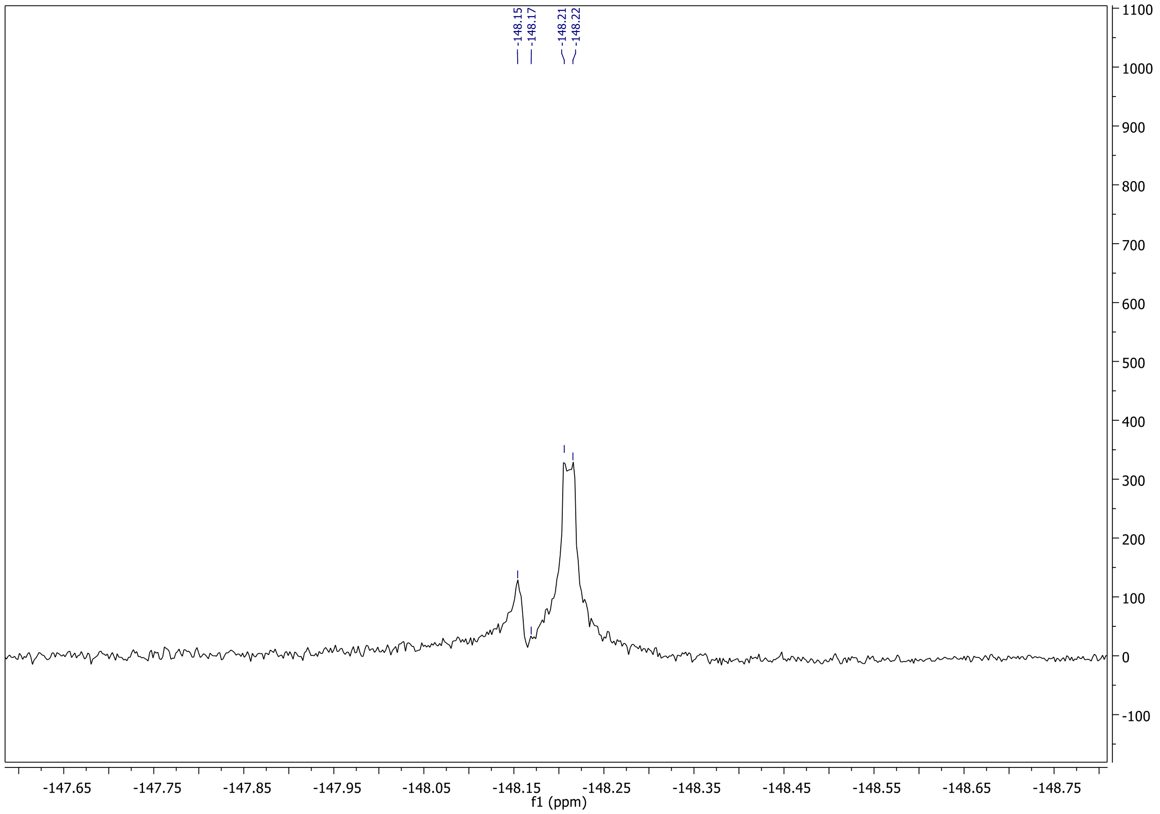

Figure S43: ^{19}F NMR of Compound **23**

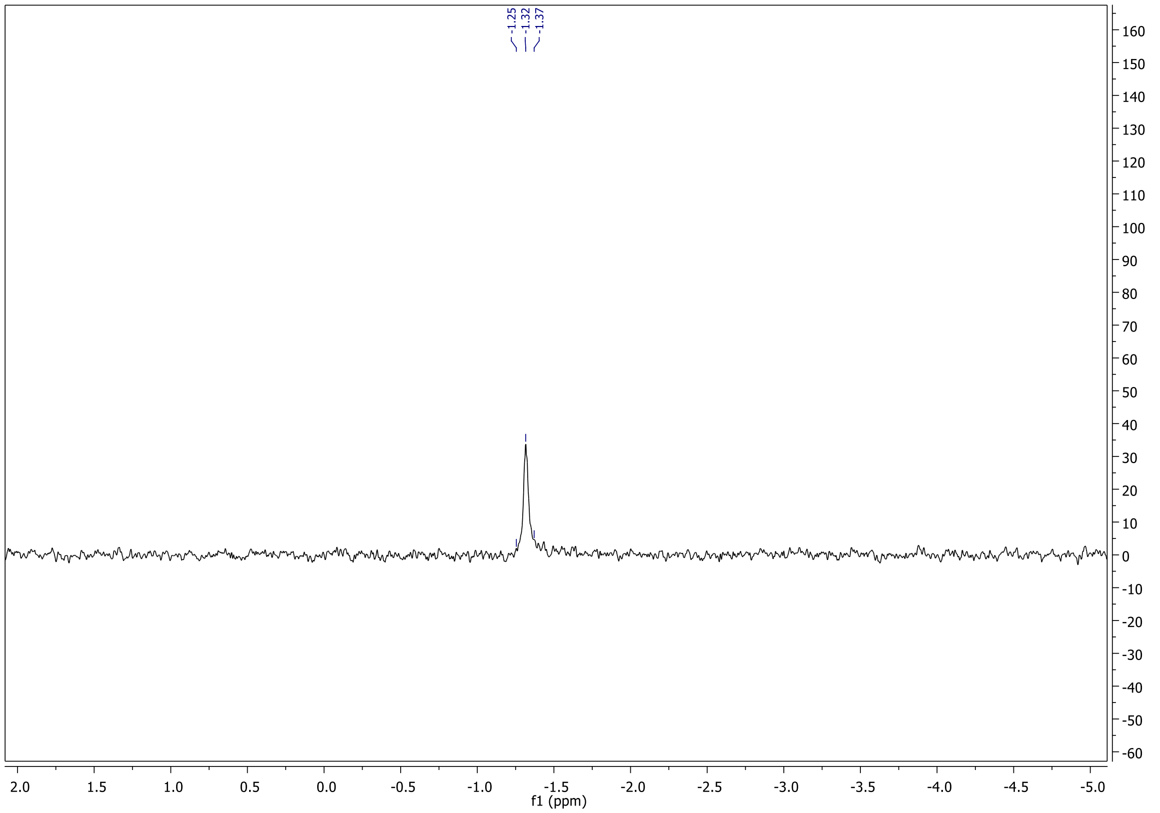

Figure S44: ^{11}B NMR of Compound **23**

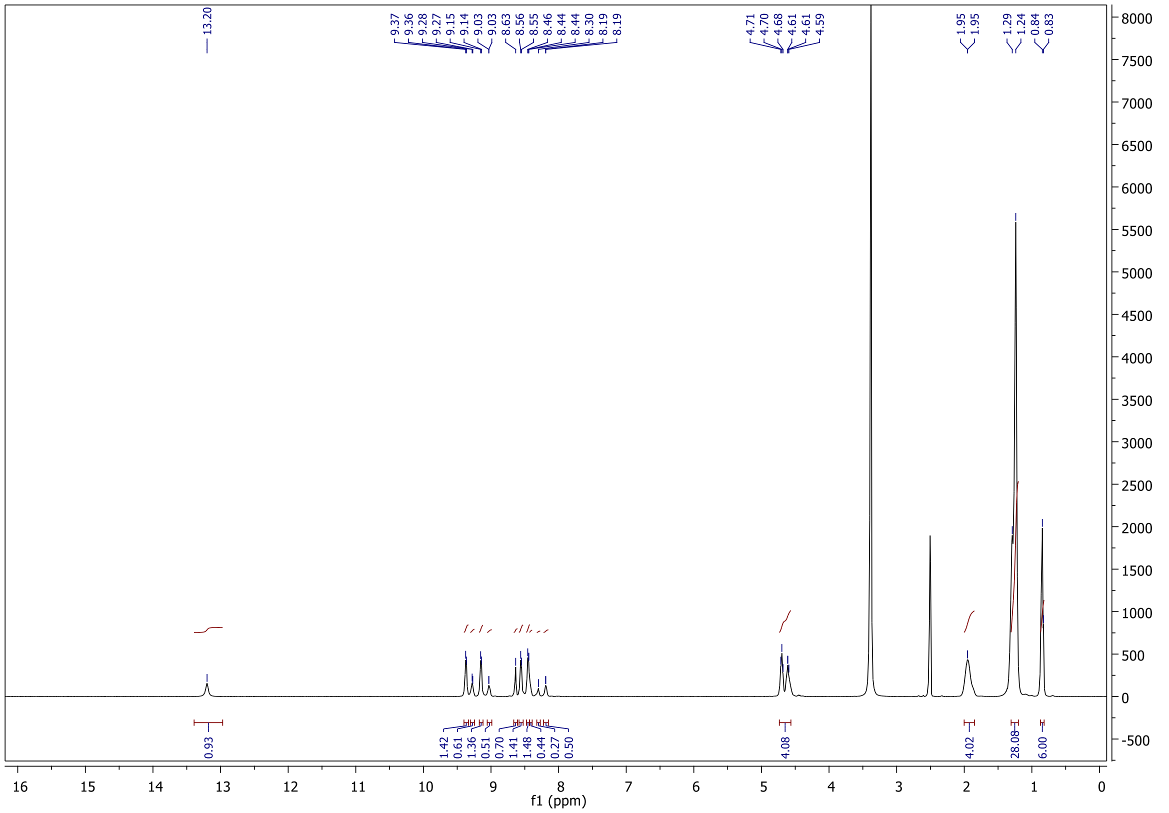

Figure S45: ^{1}H NMR of Compound **24**

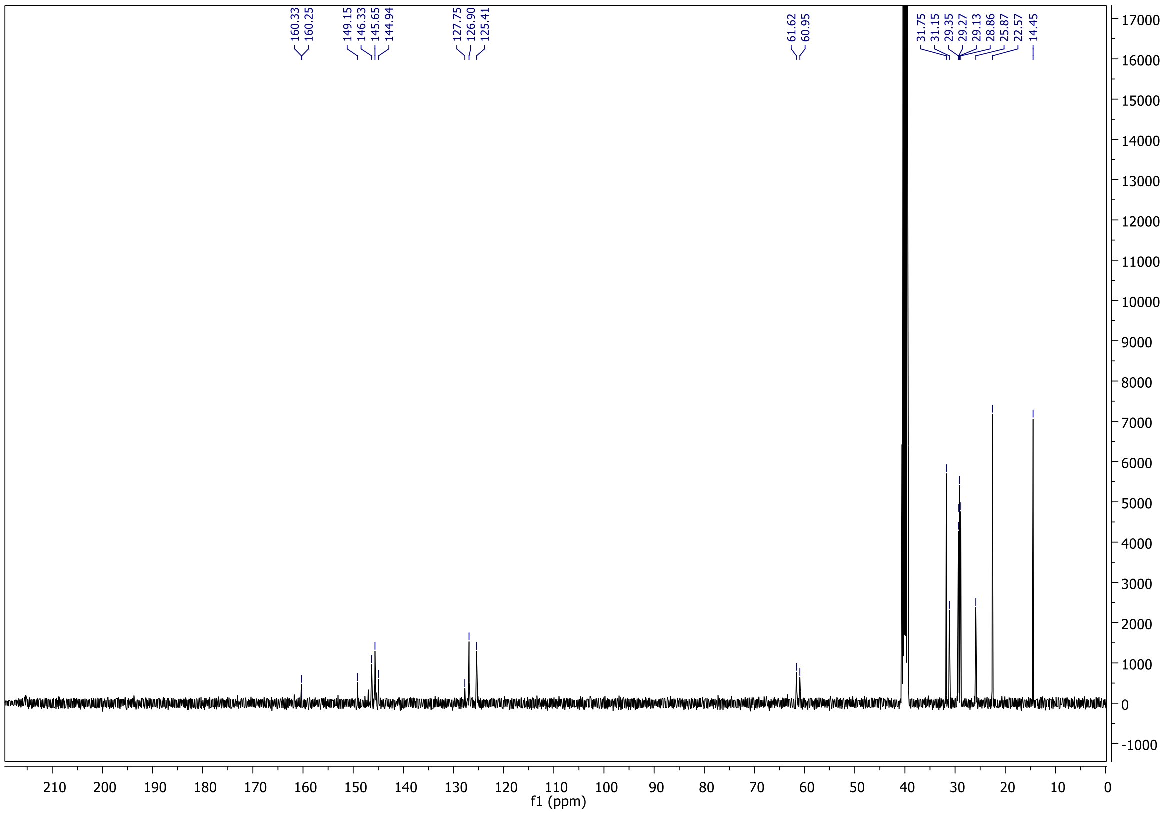

Figure S46: ^{13}C NMR of Compound **24**

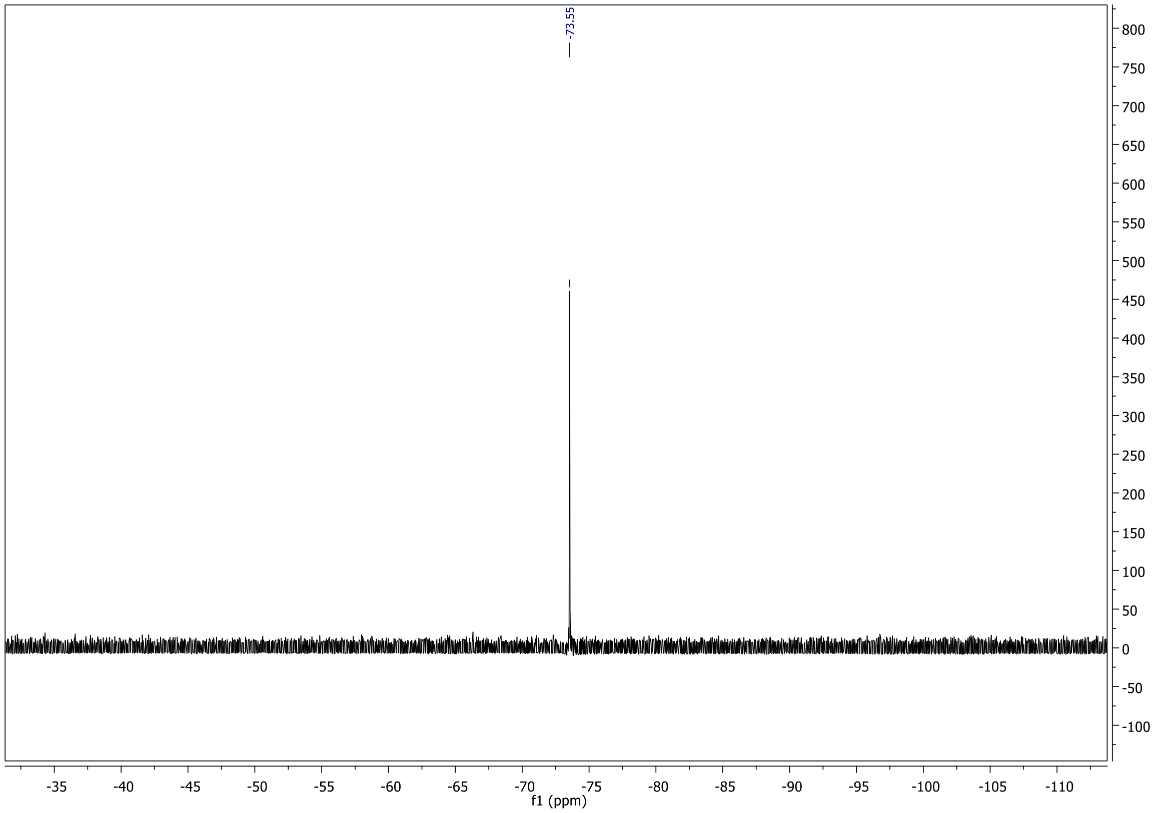

Figure S47: ^{19}F NMR of Compound **24**

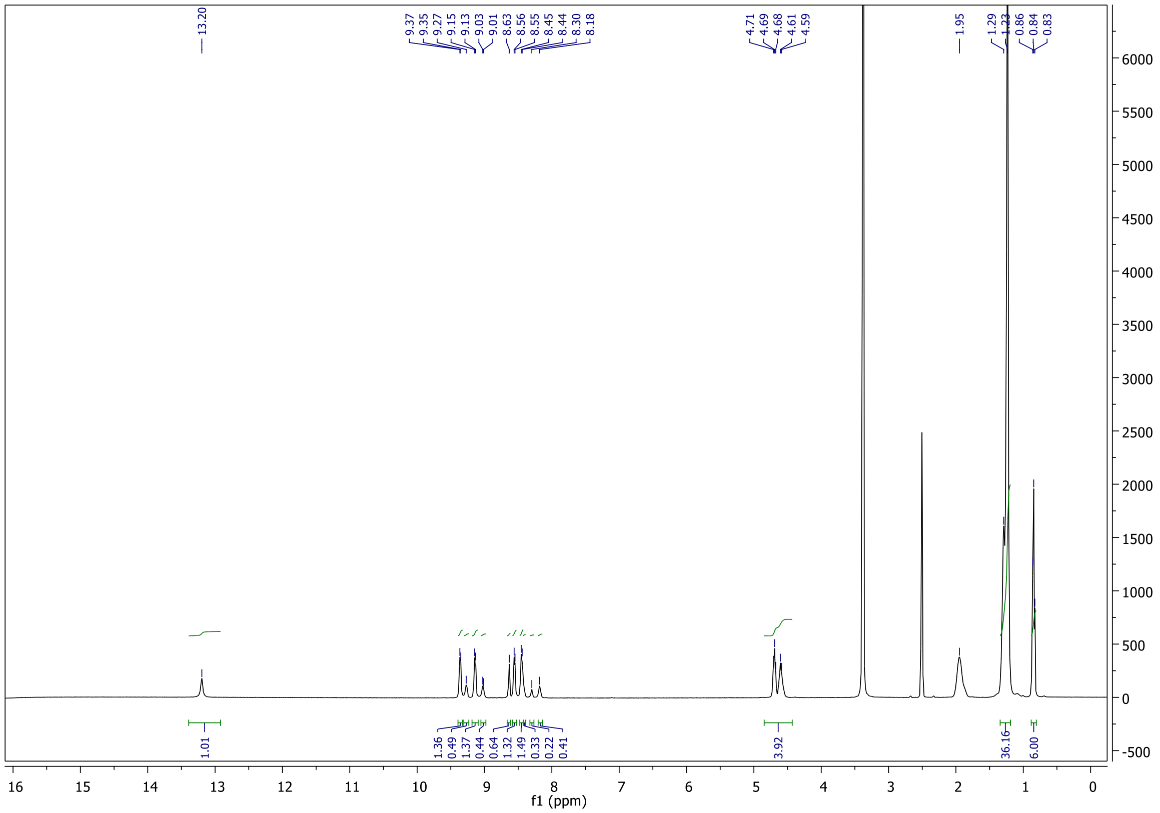

Figure S48: ^{1}H NMR of Compound **25**

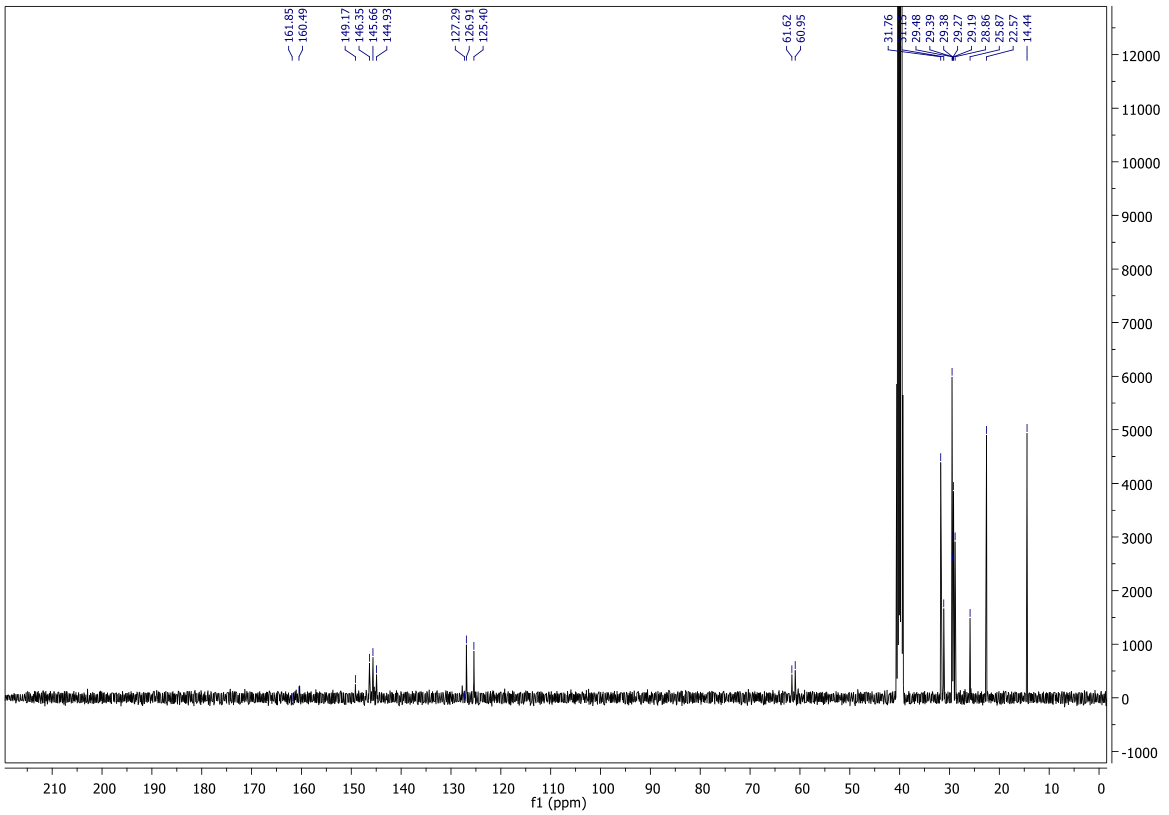

Figure S49: ^{13}C NMR of Compound **25**

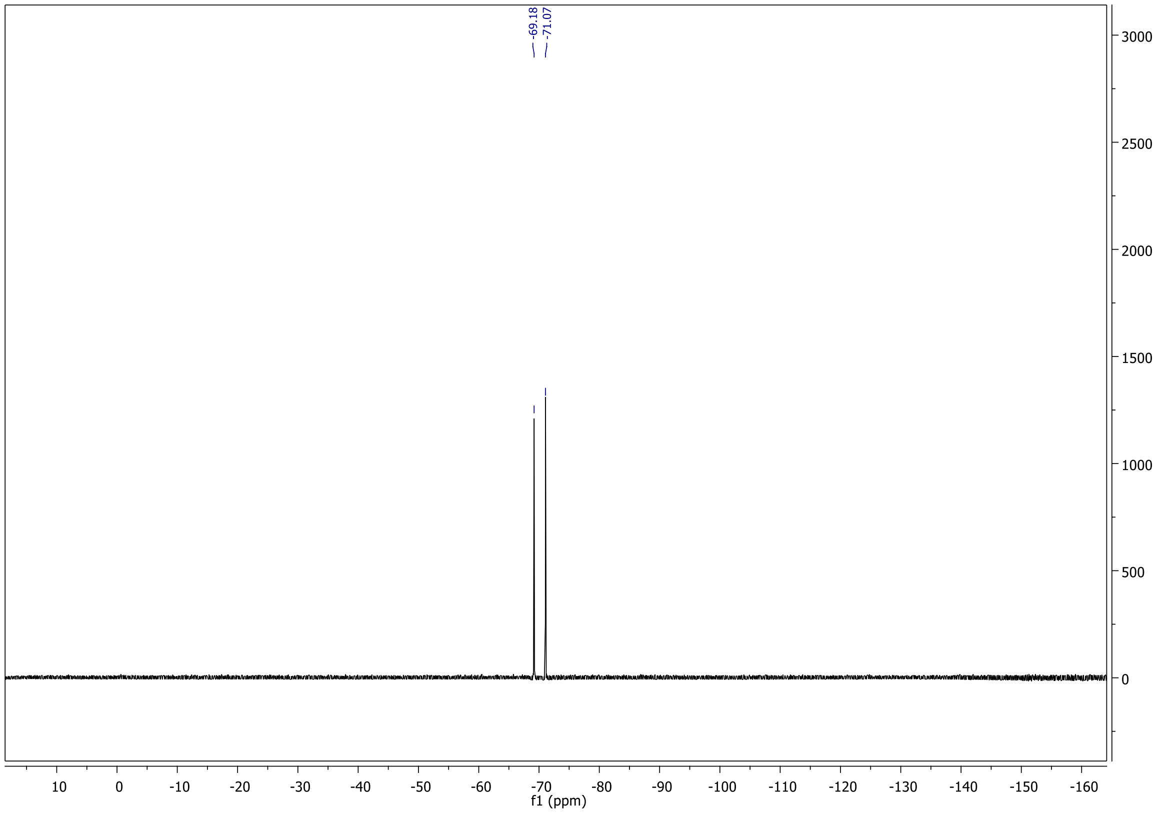

Figure S50: ^{19}F NMR of Compound **25**

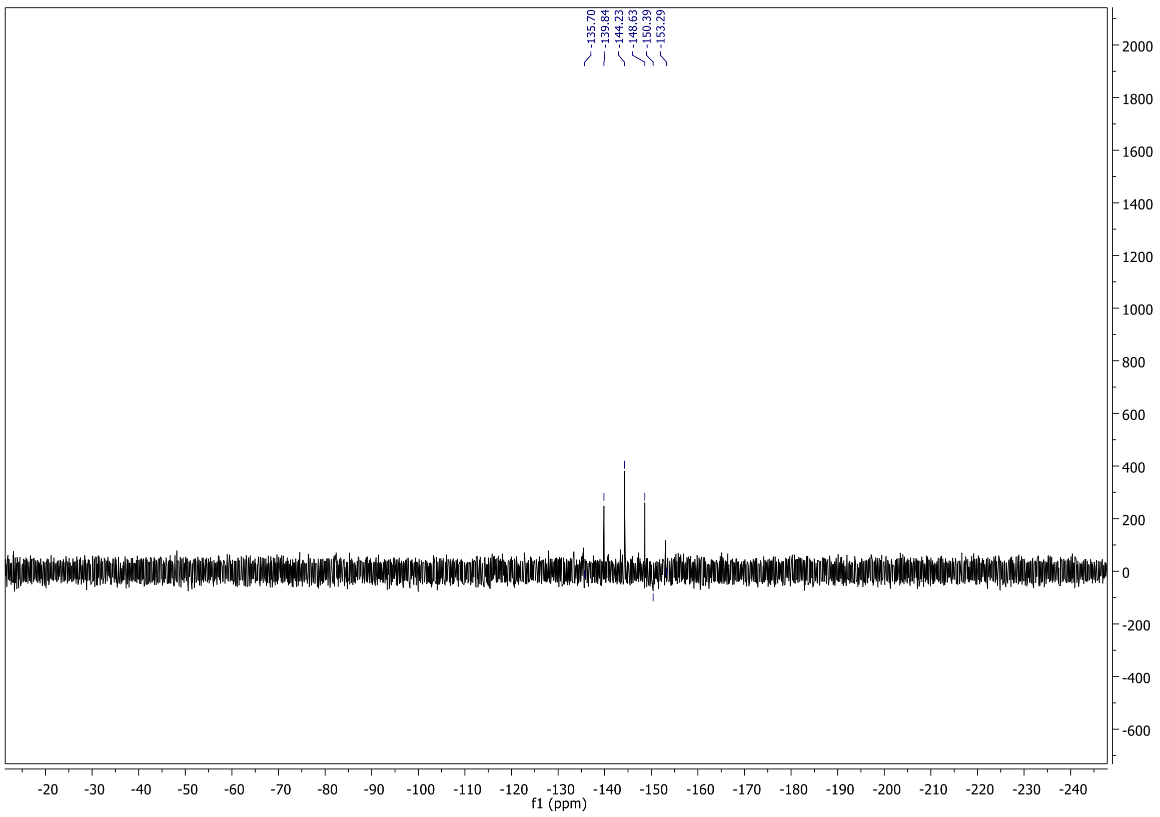

Figure S51: ^{31}P NMR of Compound **25**

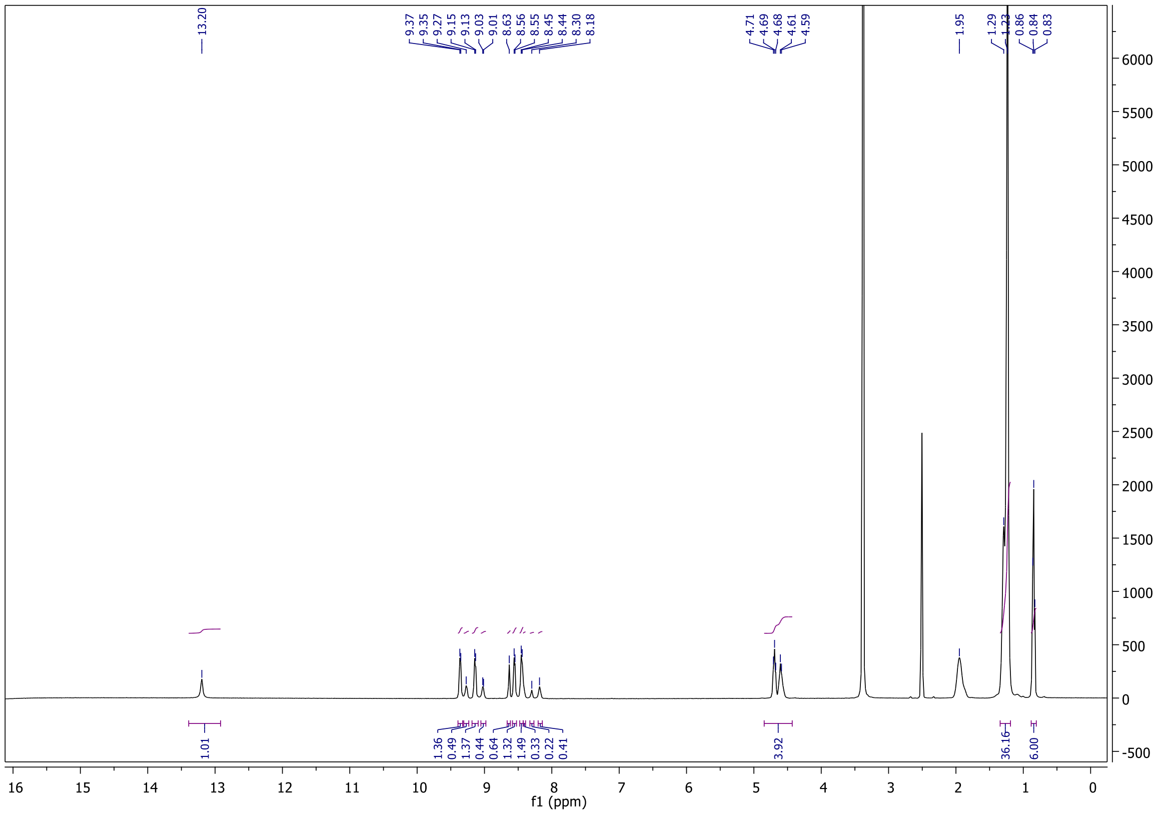

Figure S52: ^{1}H NMR of Compound **26**

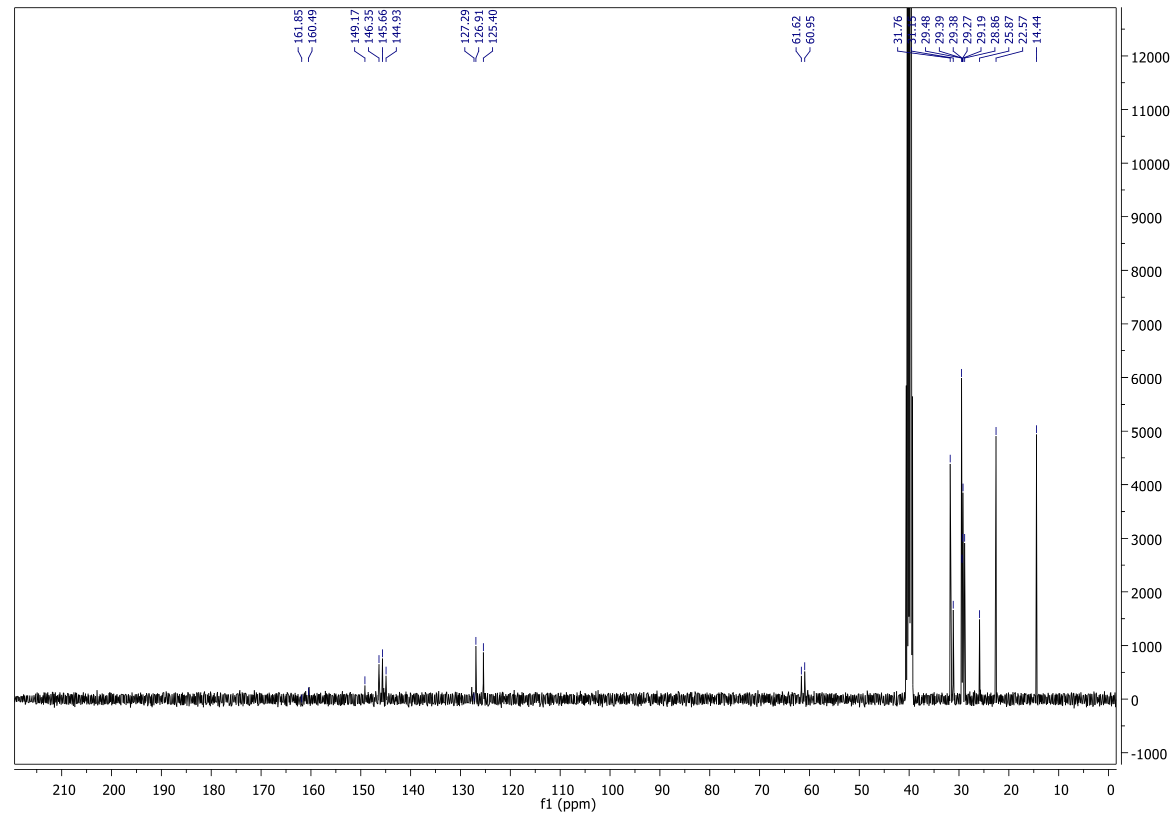

Figure S53: ^{13}C NMR of Compound **26**

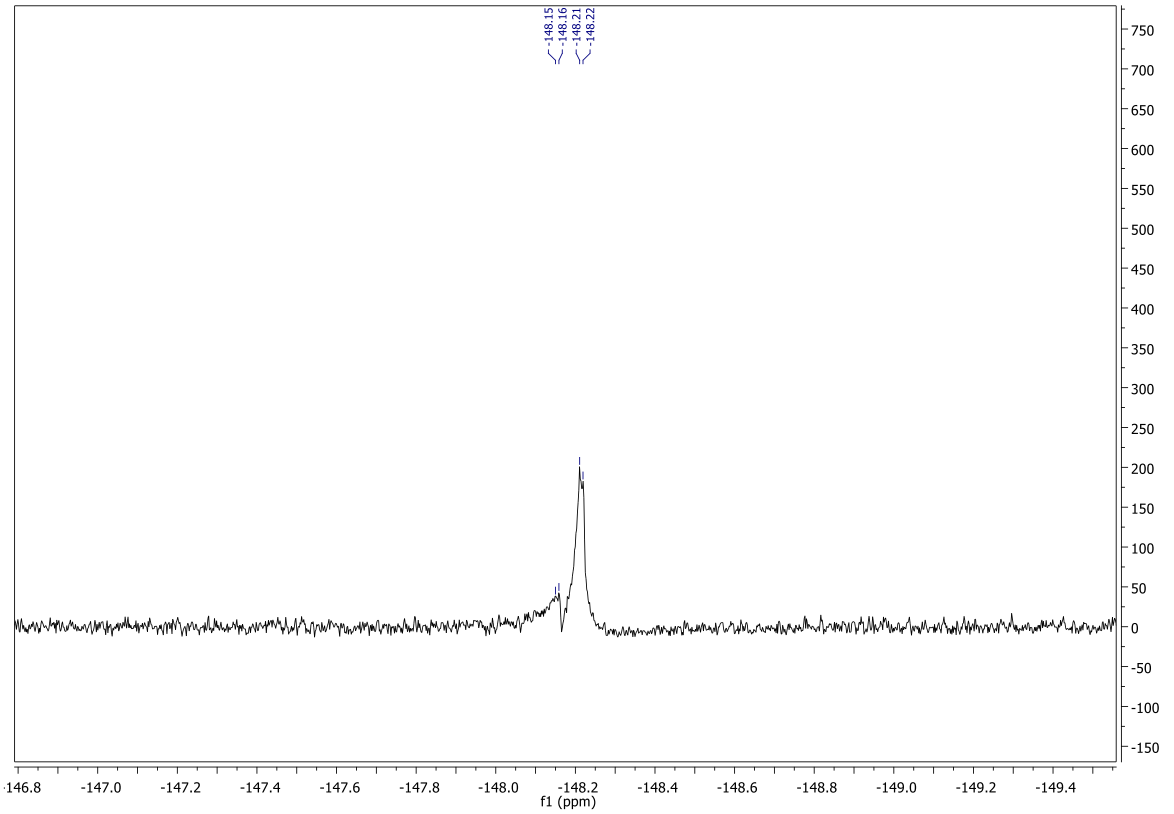

Figure S54: ^{19}F NMR of Compound **26**

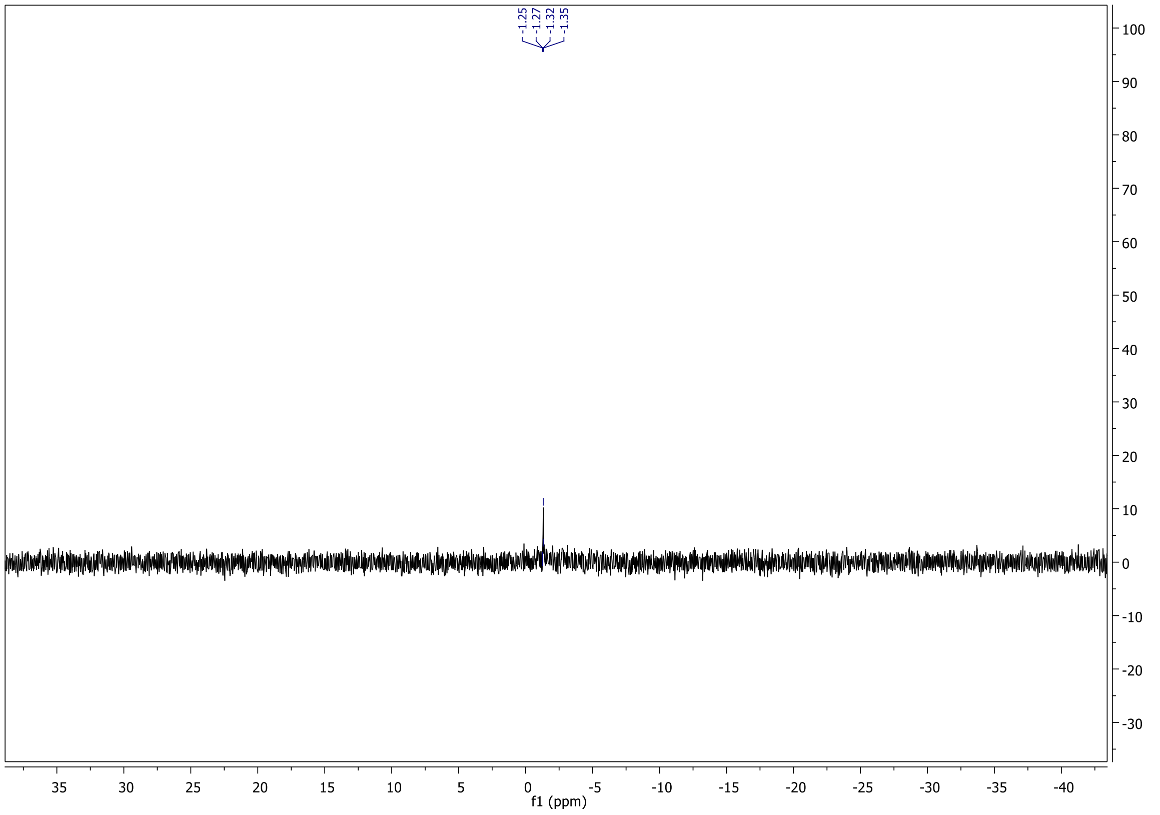

Figure S55: ^{11}B NMR of Compound **26**

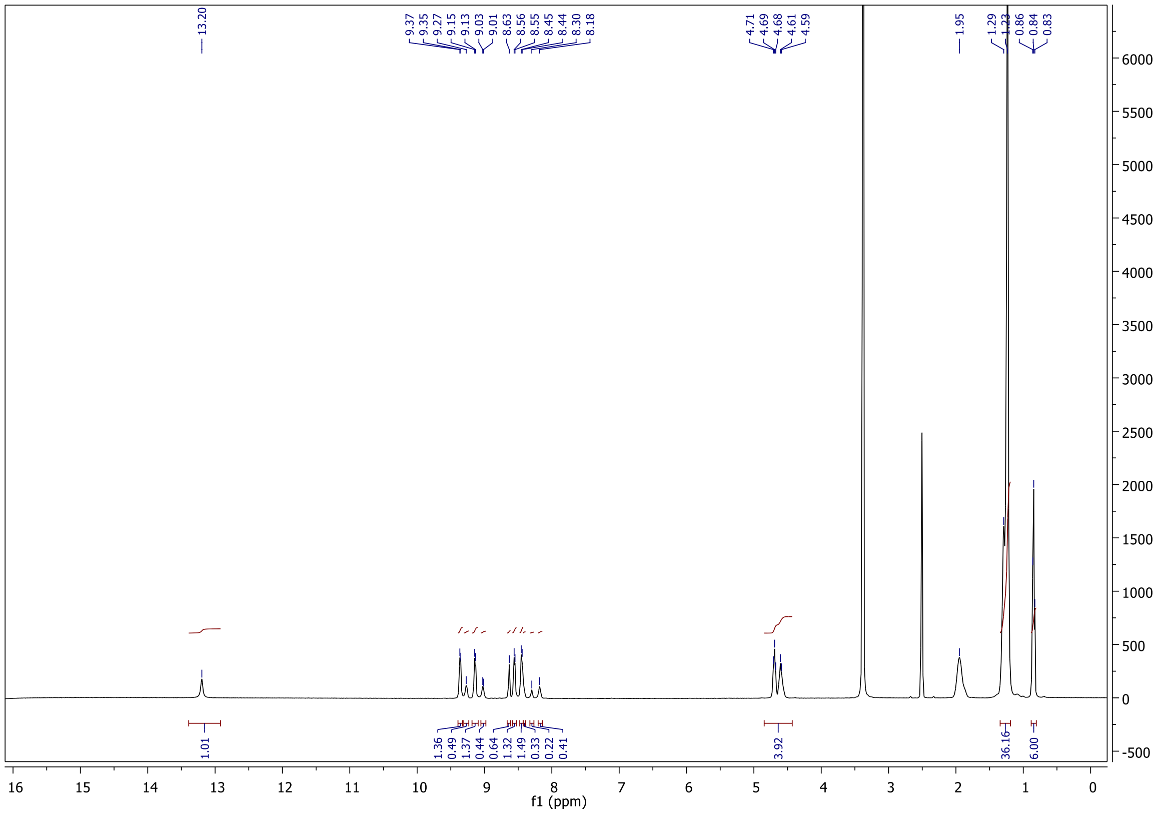

Figure S56: ^{1}H NMR of Compound **27**

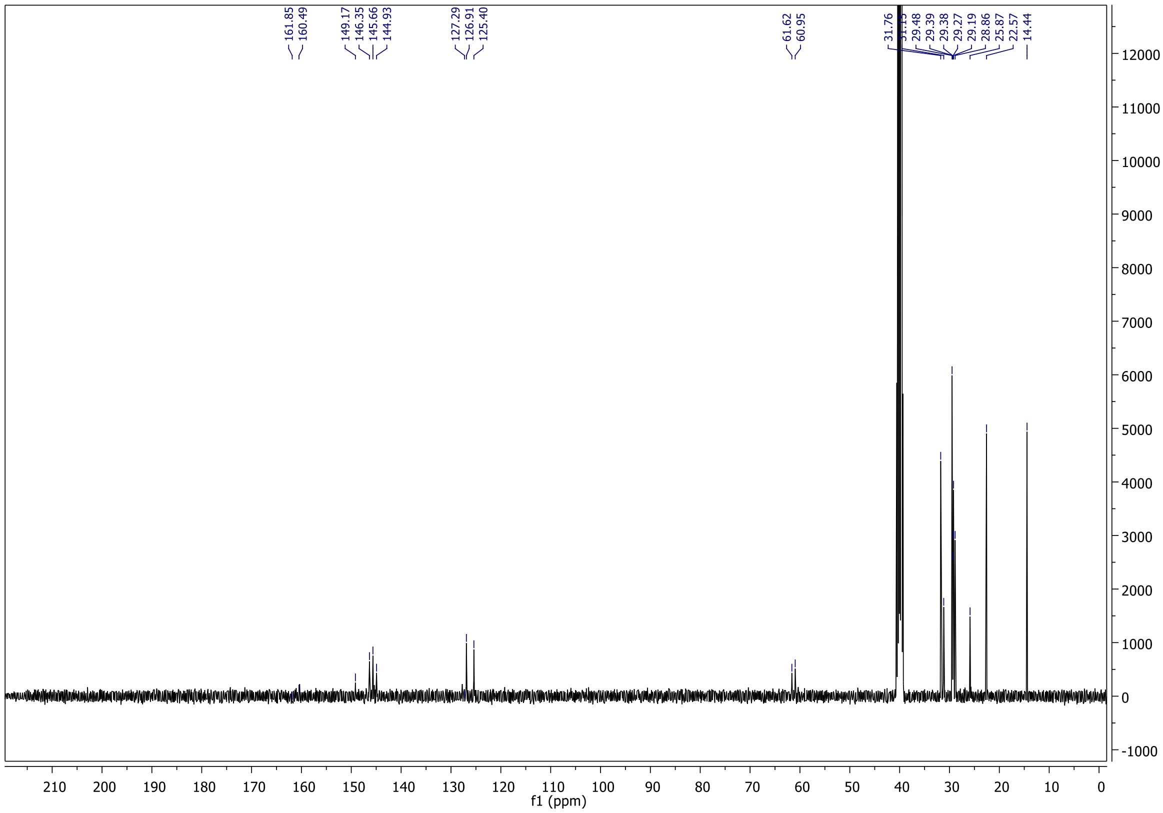

Figure S57: ^{13}C NMR of Compound **27**

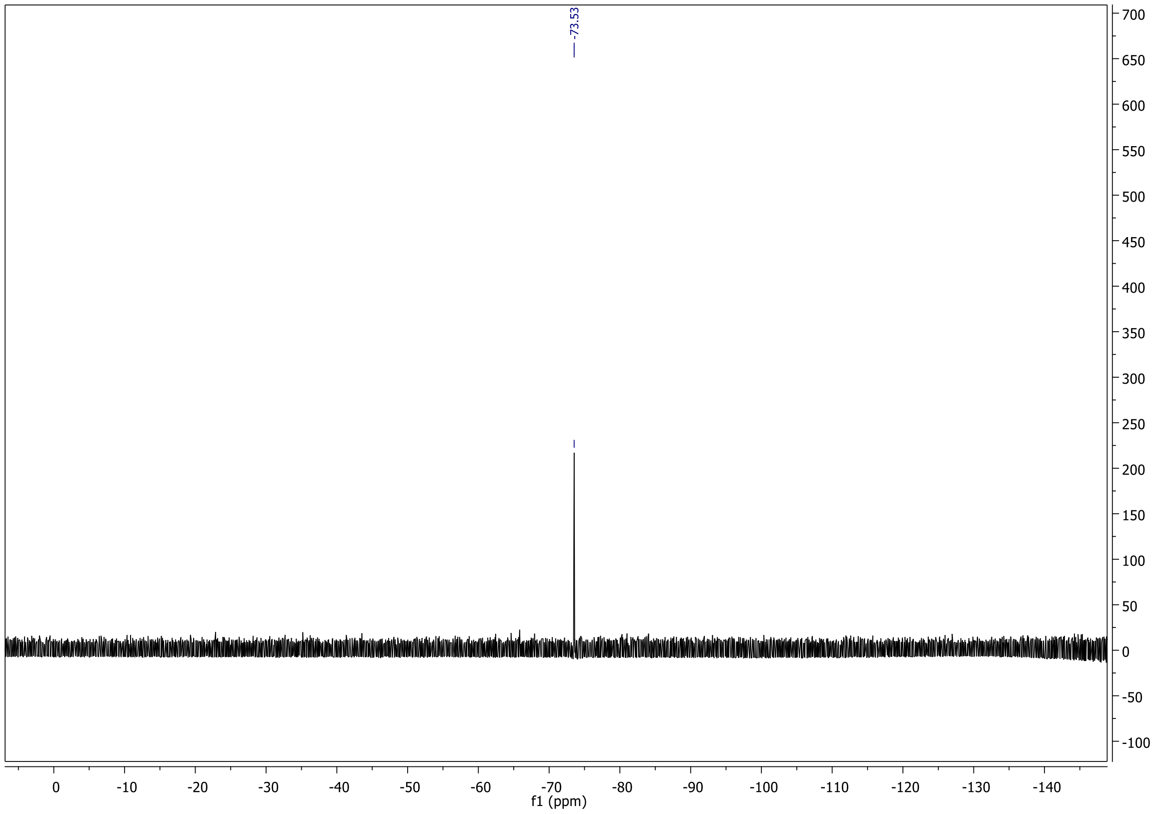

Figure S58: ^{19}F NMR of Compound **27**

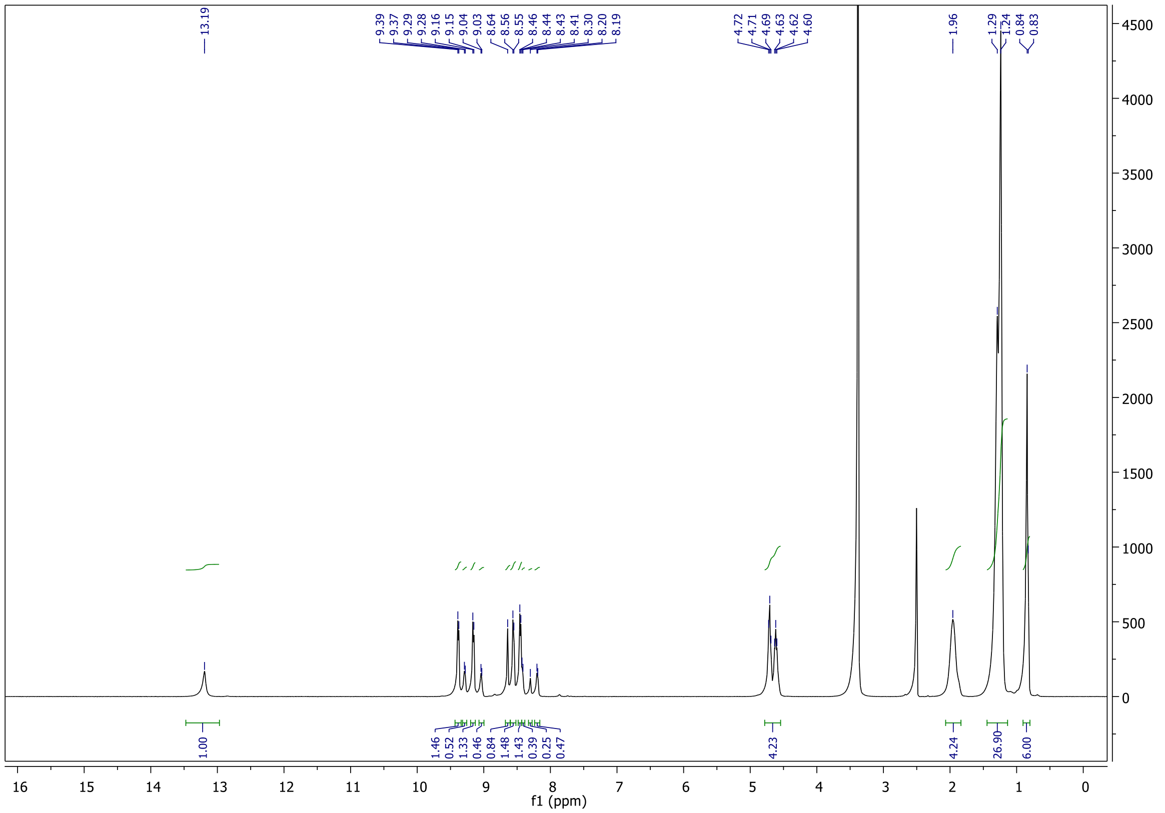

Figure S59: ^{1}H NMR of Compound **28**

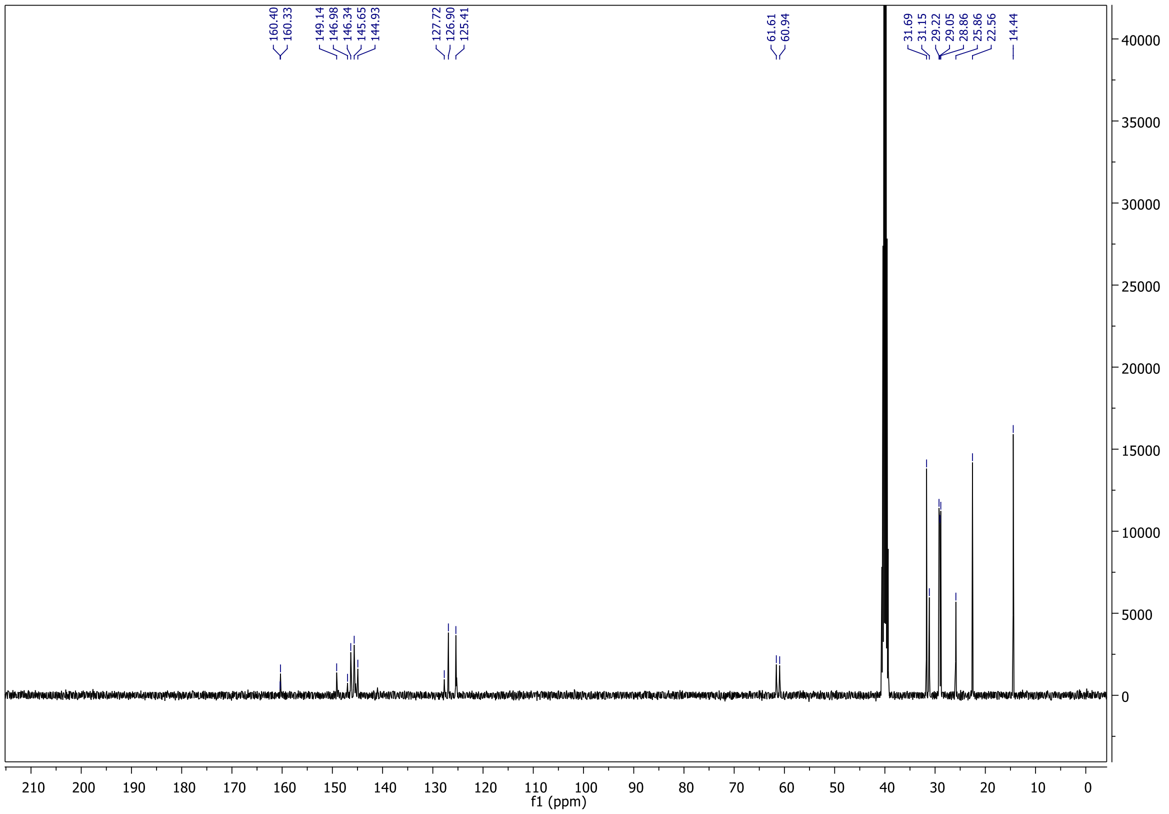

Figure S60: ^{13}C NMR of Compound **28**

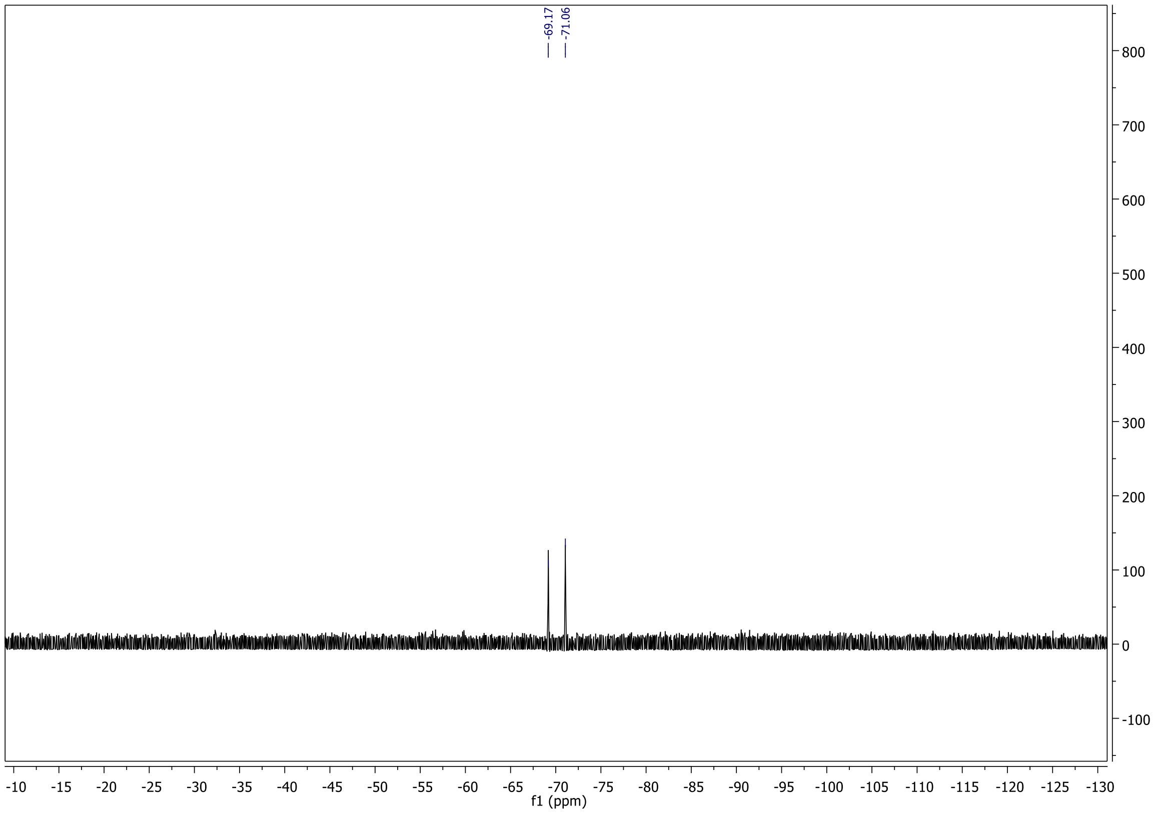

Figure S61: ^{19}F NMR of Compound **28**

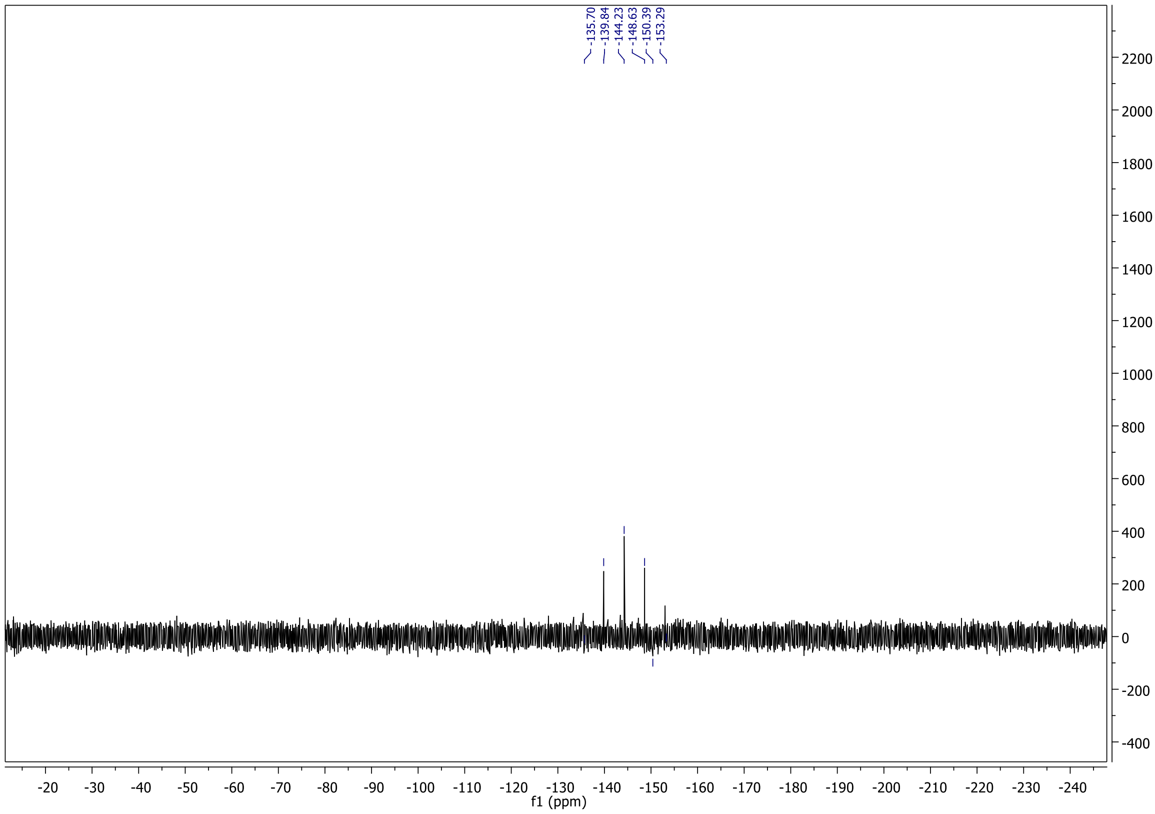

Figure S62: ^{31}P NMR of Compound **28**

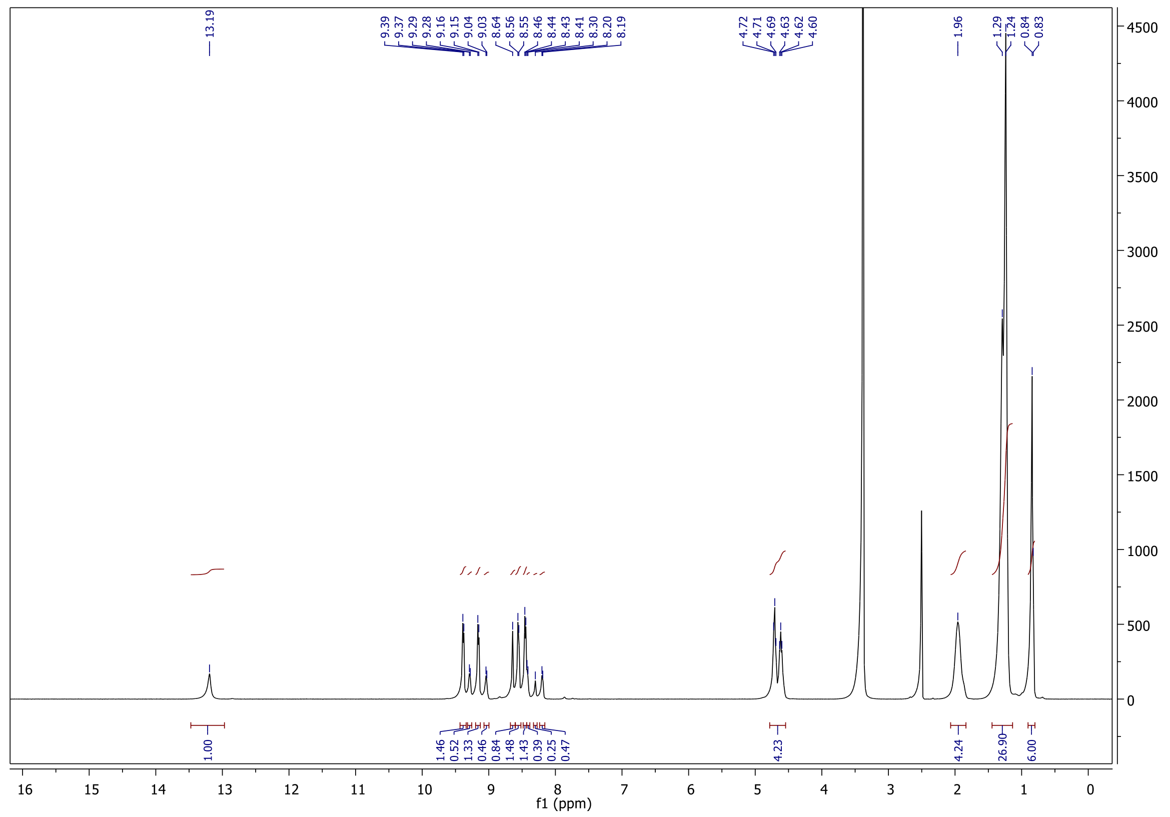

Figure S63: ^{1}H NMR of Compound **29**

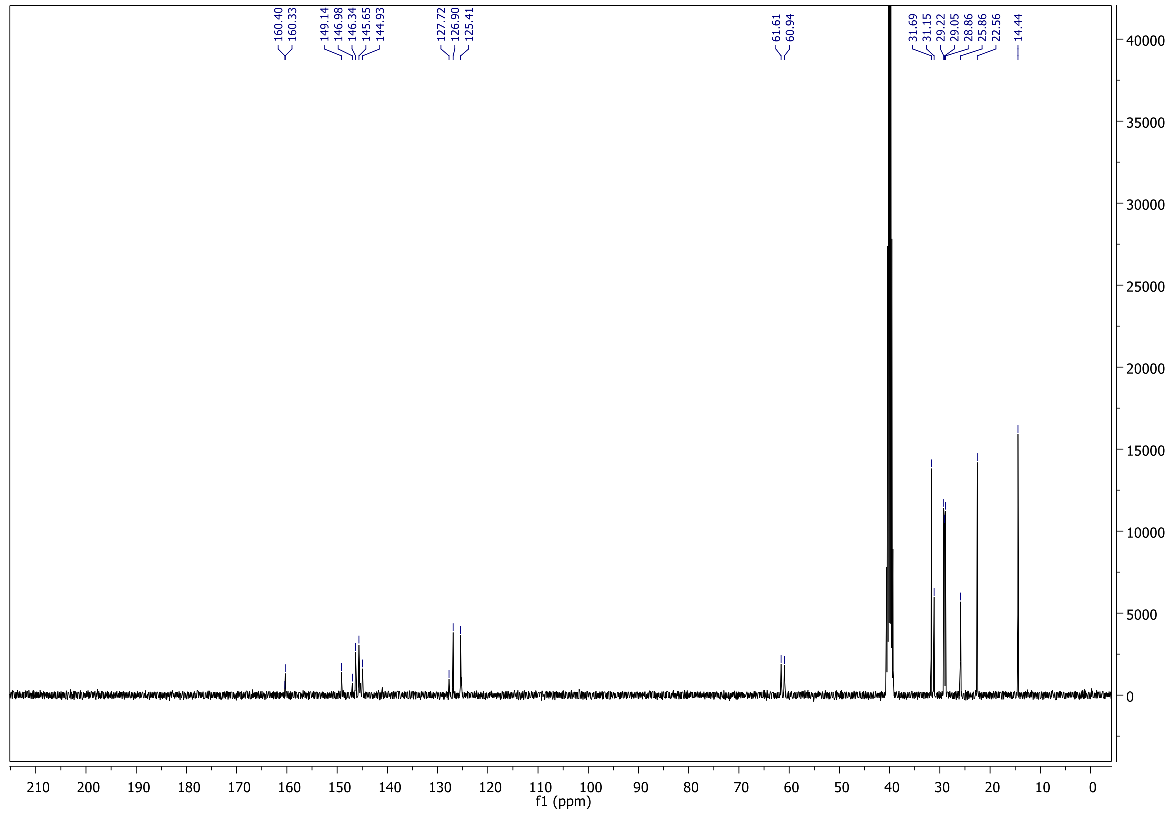

Figure S64: ^{13}C NMR of Compound **29**

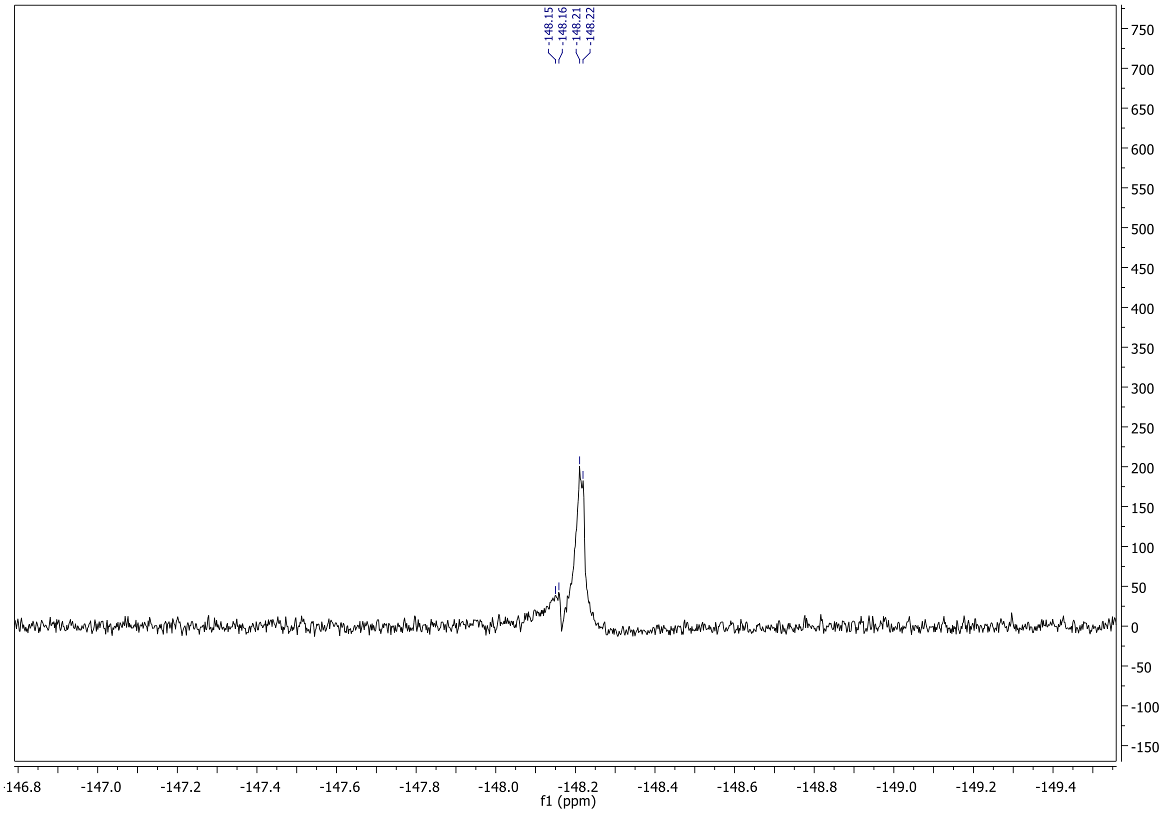

Figure S65: ^{19}F NMR of Compound **29**

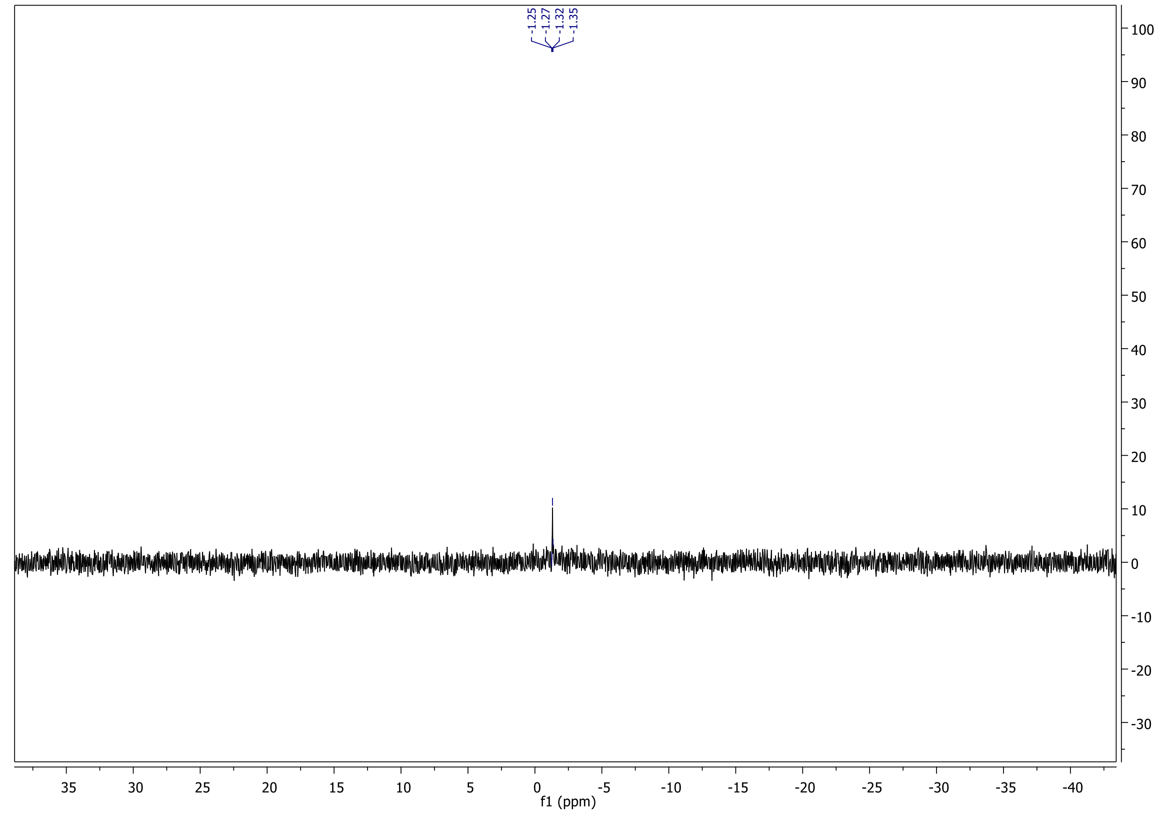

Figure S66: ^{11}B NMR of Compound **29**

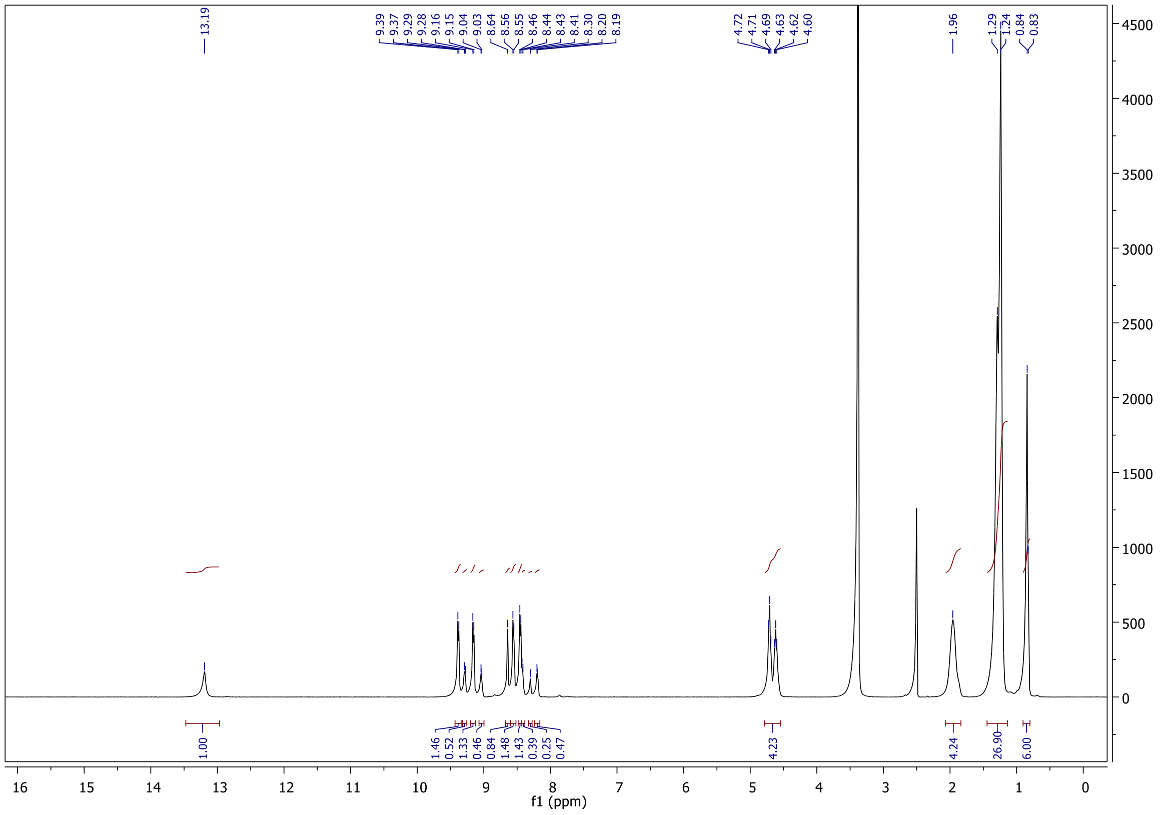

Figure S67: ^{1}H NMR of Compound **30**

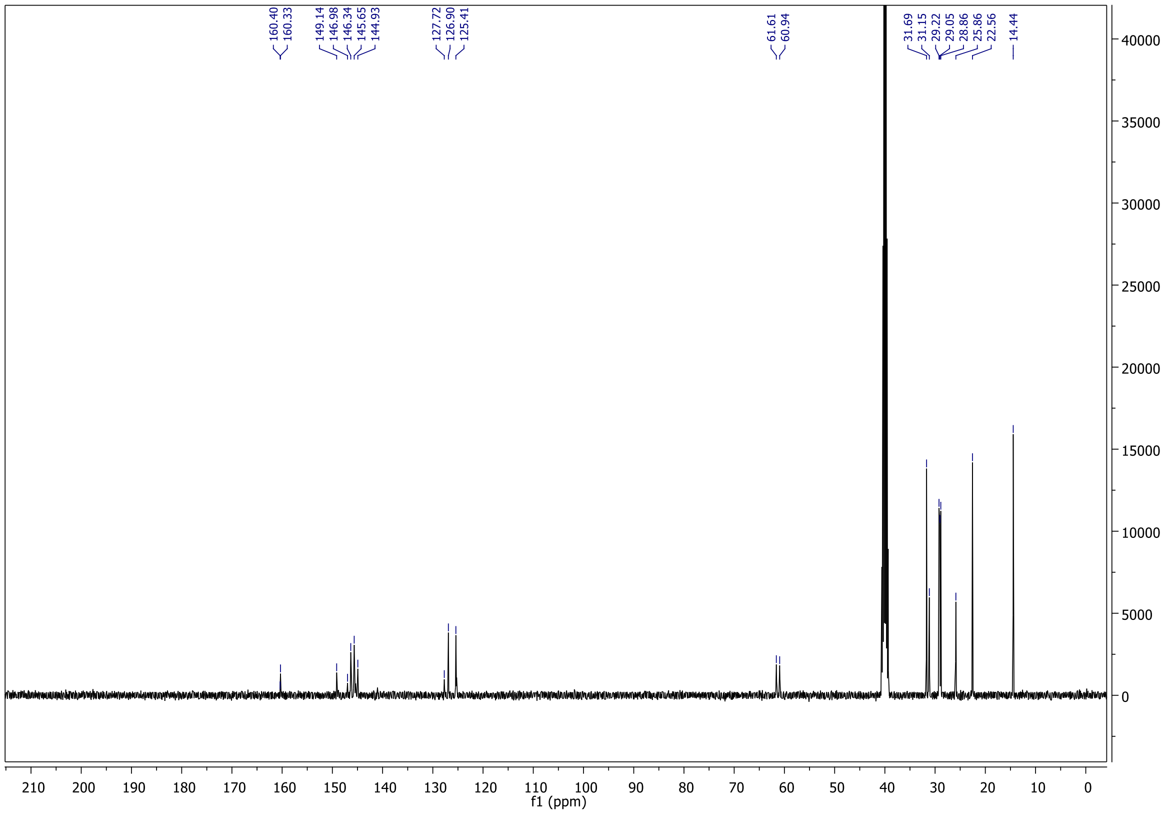

Figure S68: ^{13}C NMR of Compound **30**

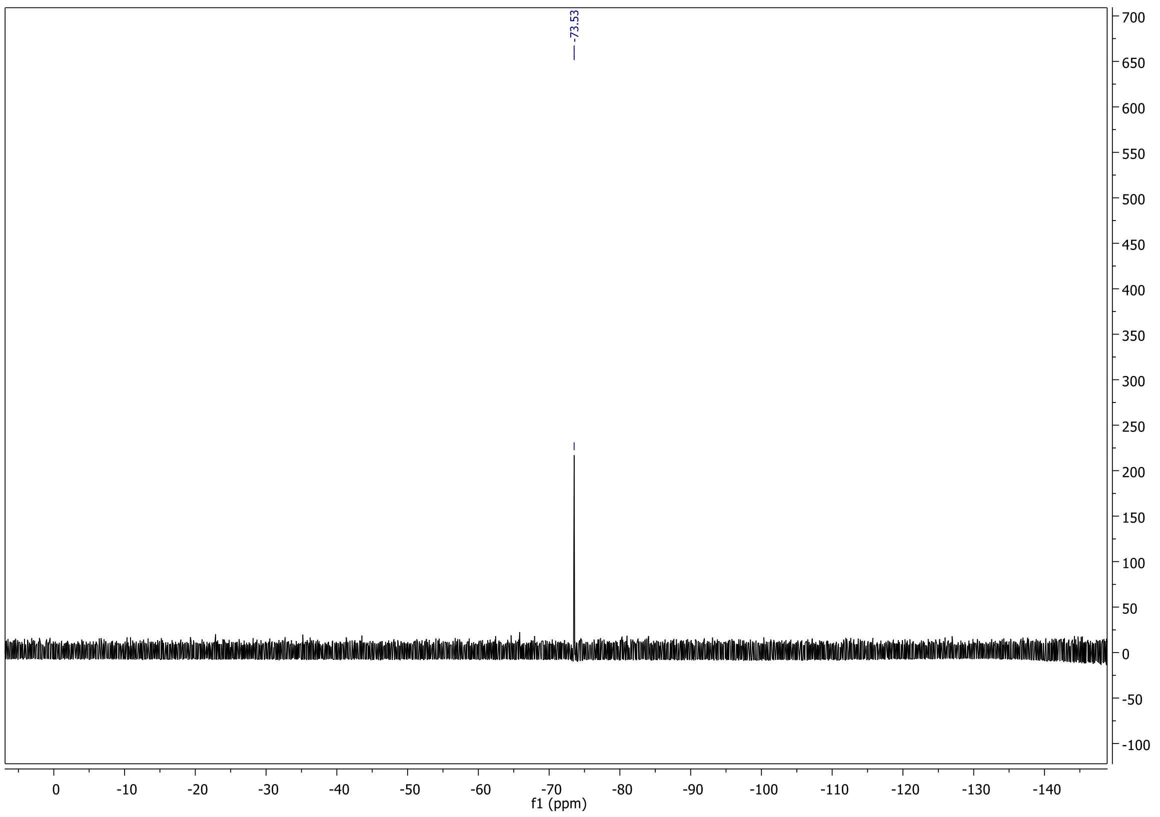

Figure S69: ^{19}F NMR of Compound **30**

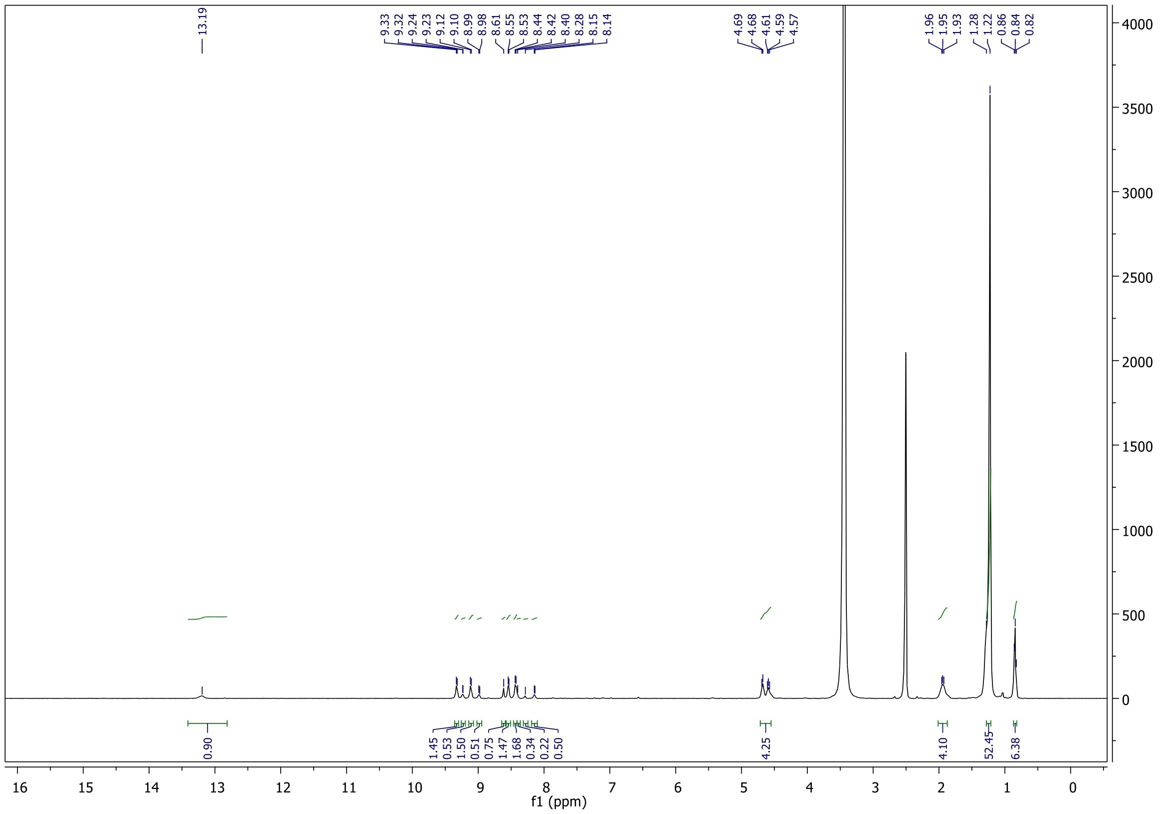

Figure S70: ^{1}H NMR of Compound **31**

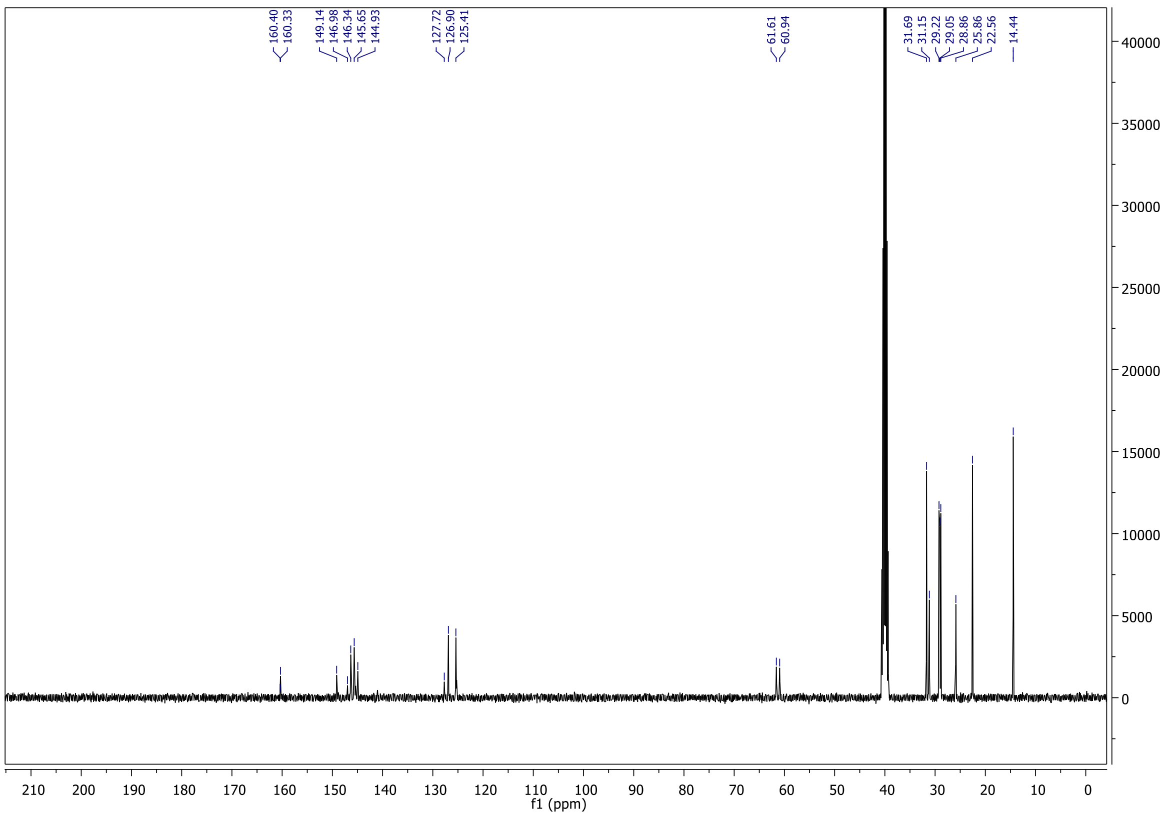

Figure S71: ^{13}C NMR of Compound **31**

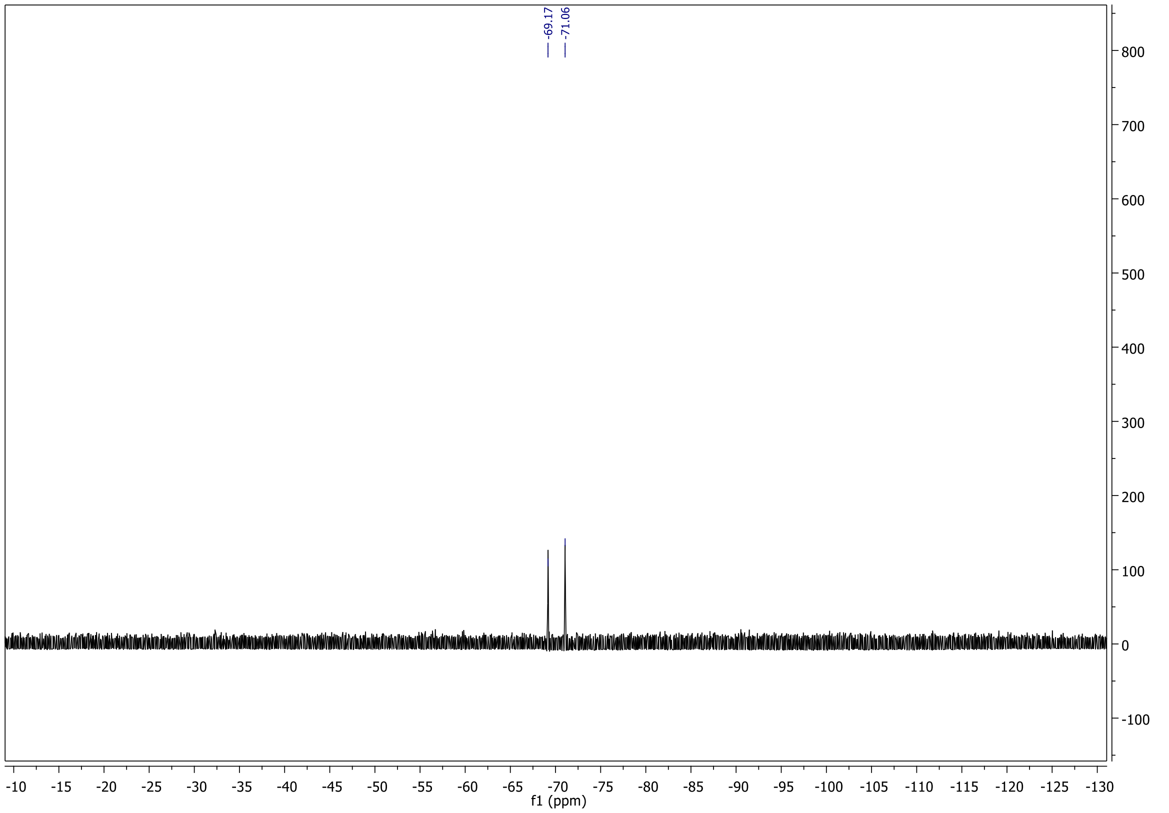

Figure S72: ^{19}F NMR of Compound **31**

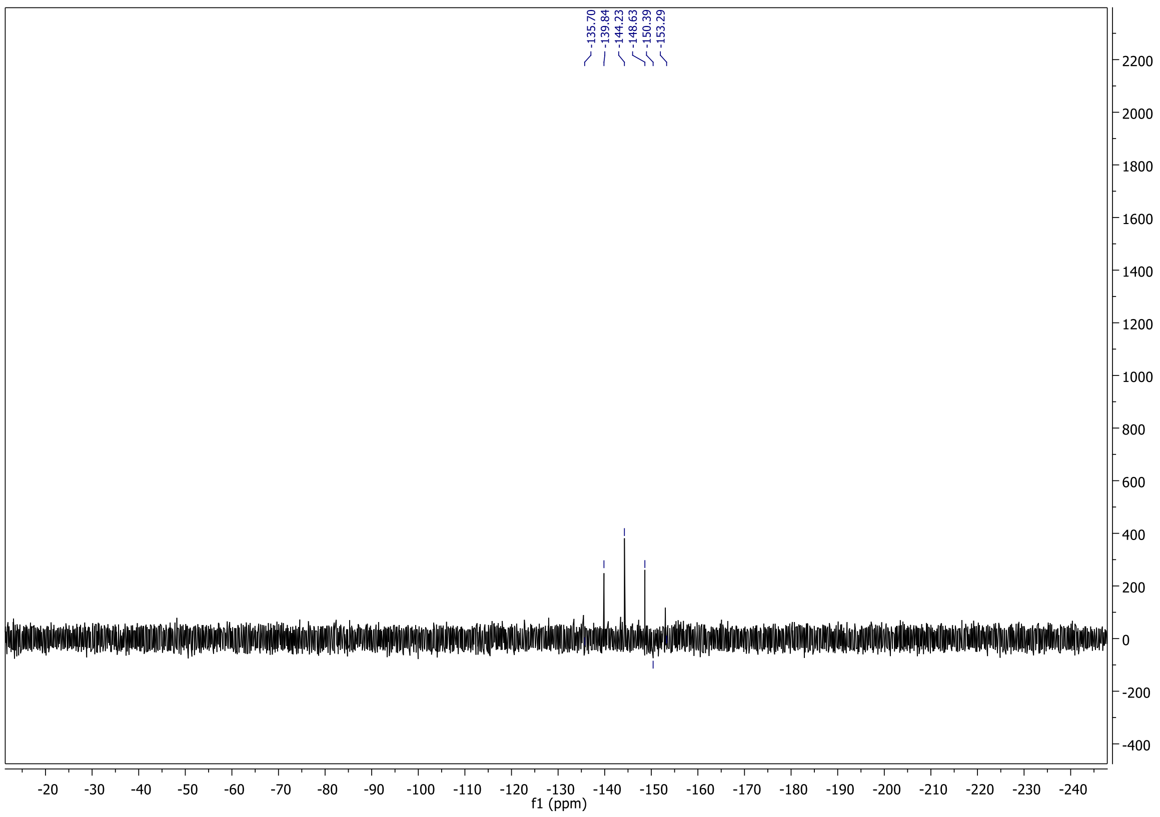

Figure S73: ^{31}P NMR of Compound **31**

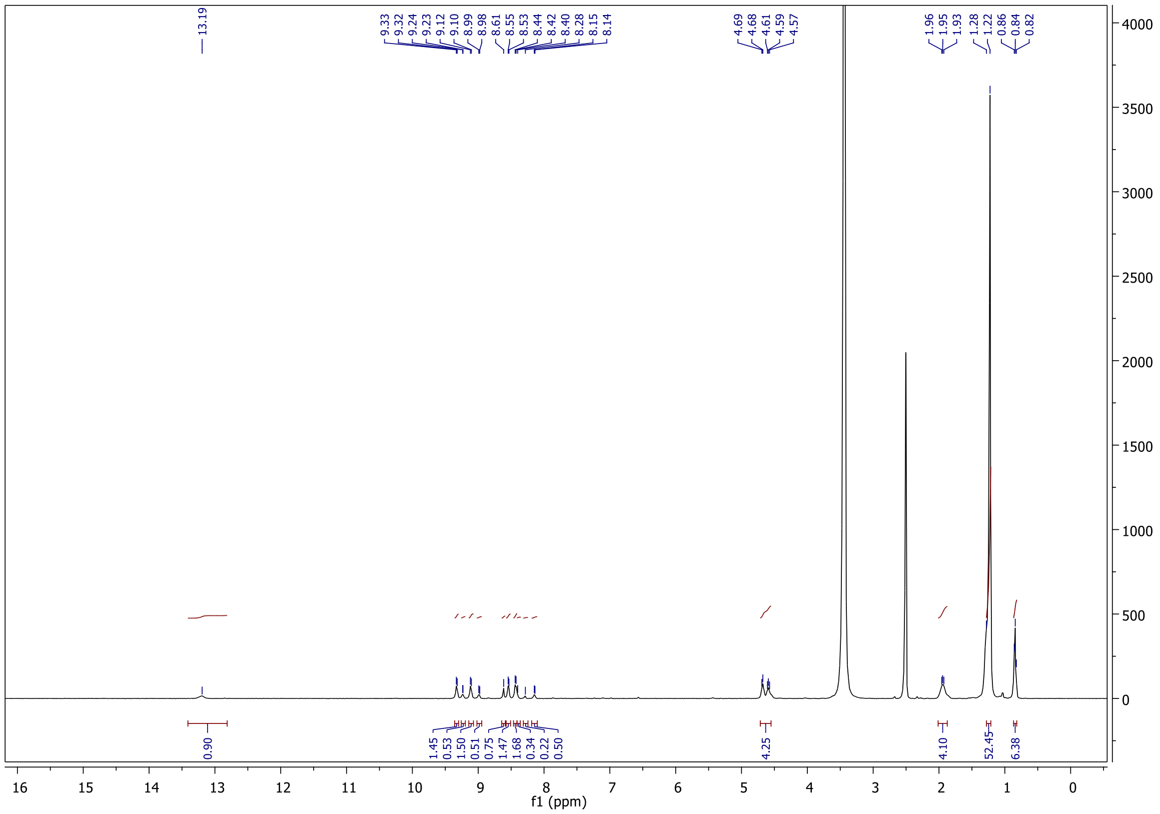

Figure S74: ^{1}H NMR of Compound **32**

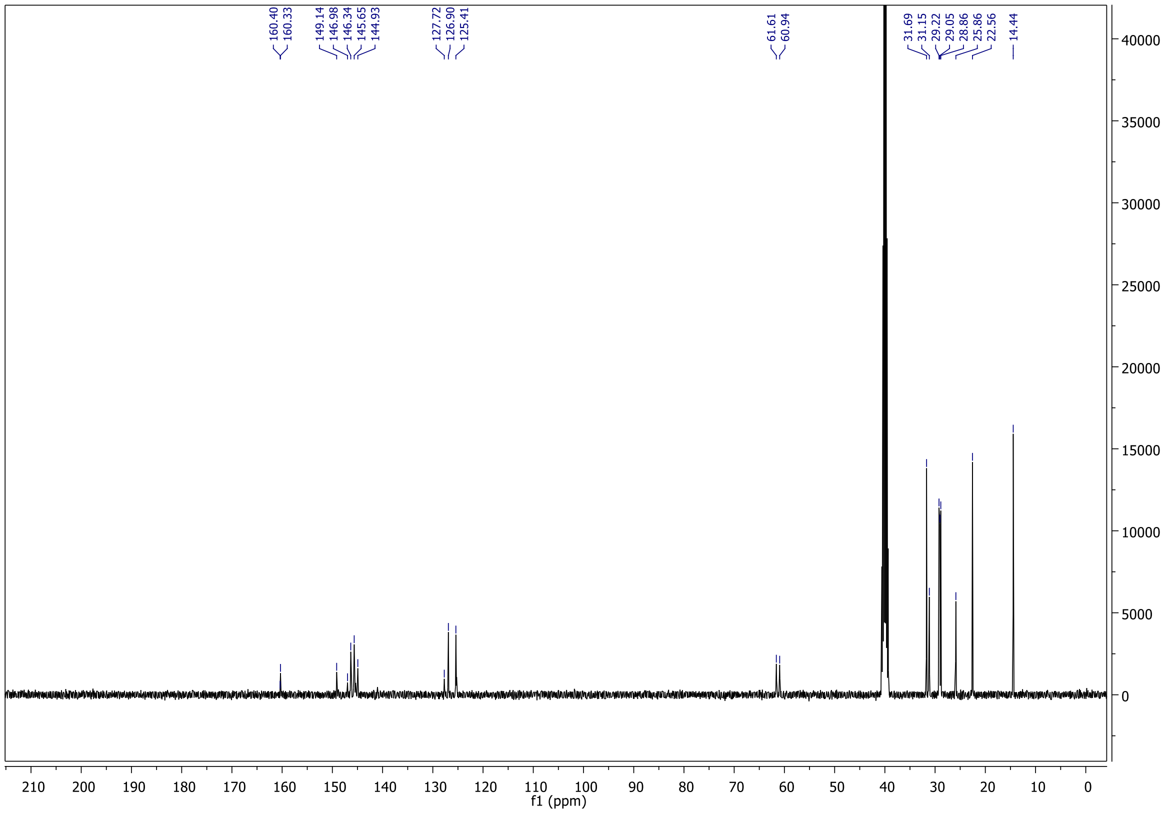

Figure S75: ^{13}C NMR of Compound **32**

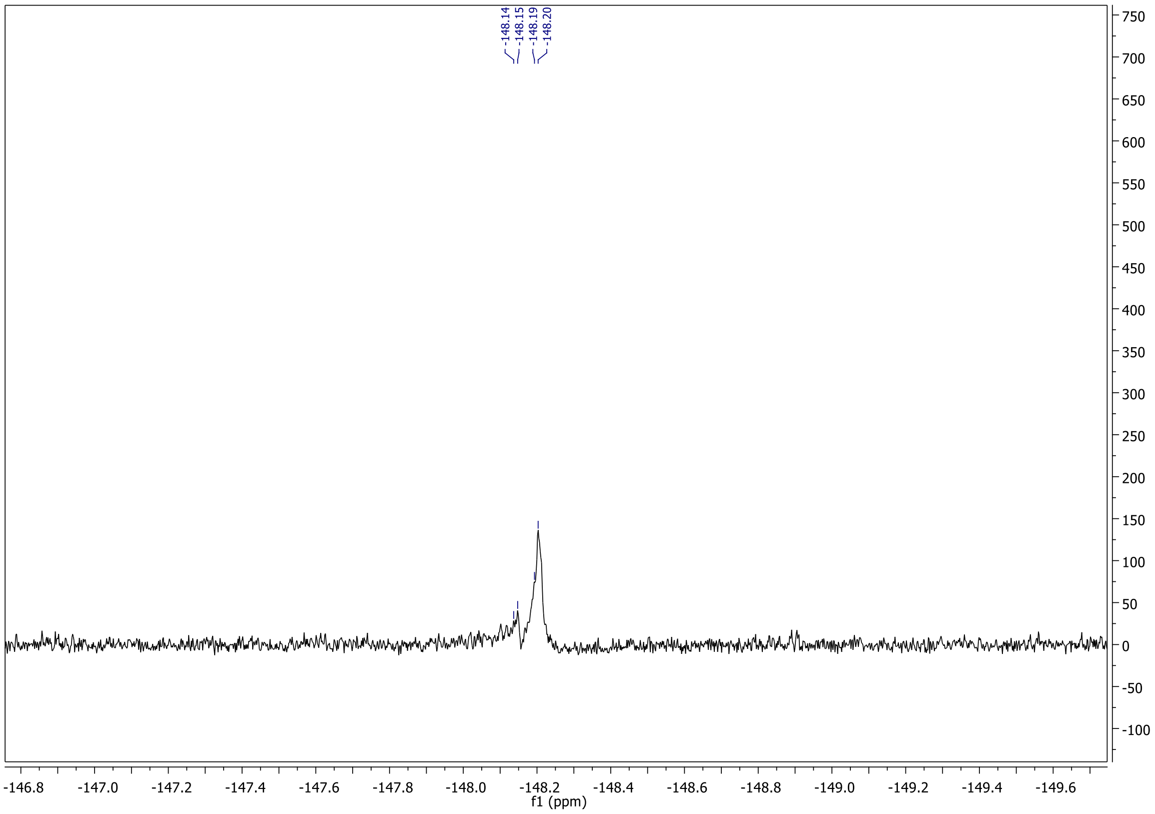

Figure S76: ^{19}F NMR of Compound **32**

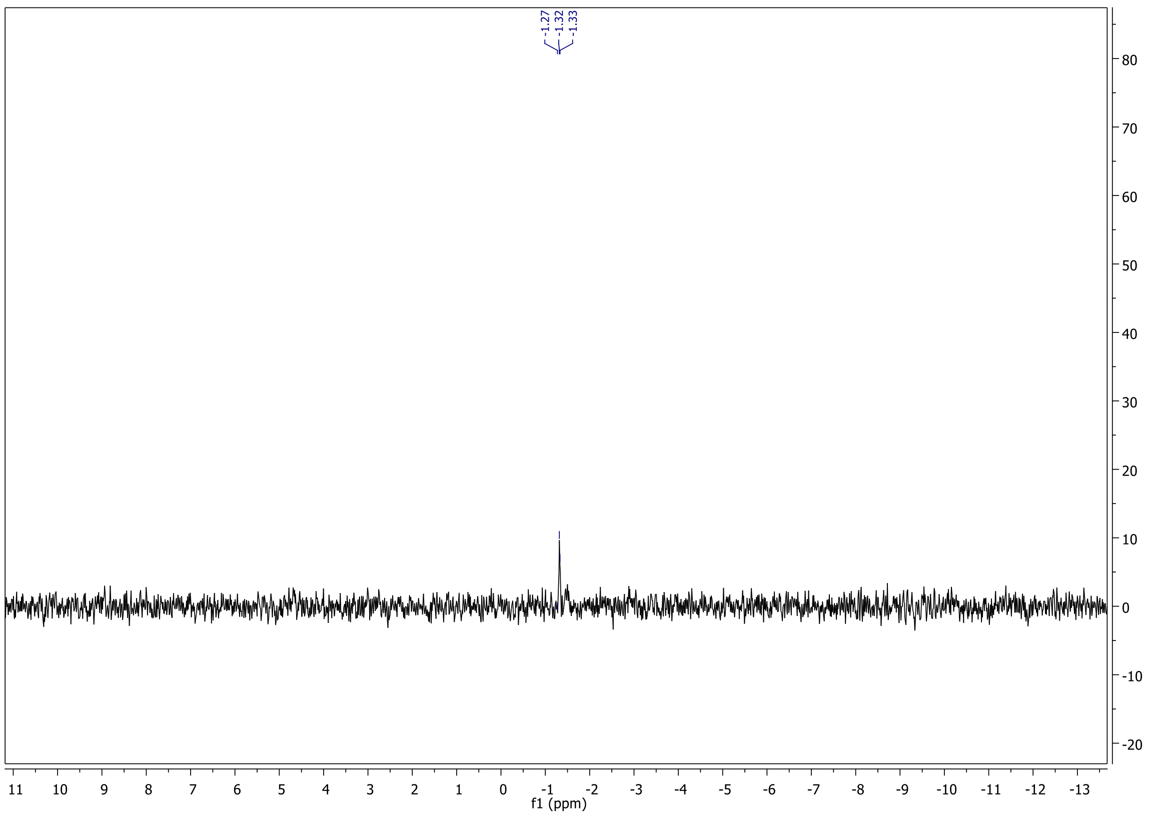

Figure S77: ^{11}B NMR of Compound **32**

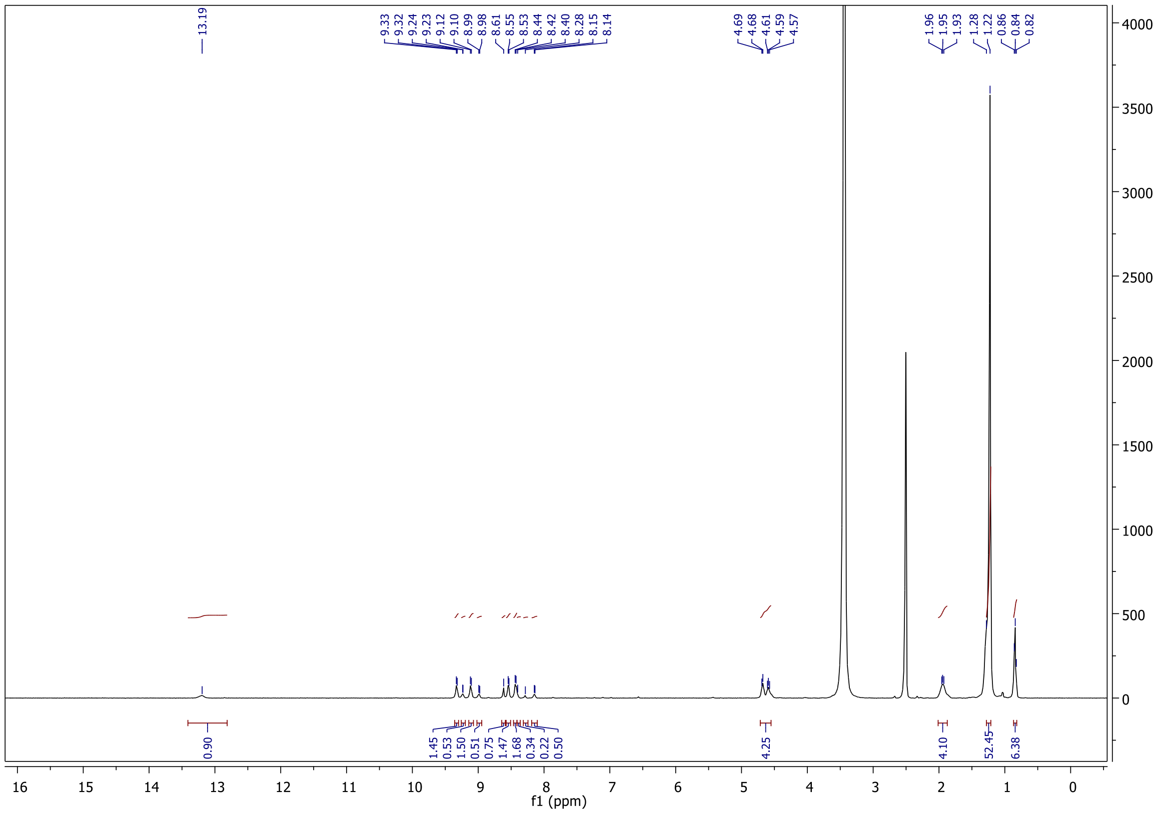

Figure S78: ^{1}H NMR of Compound **33**

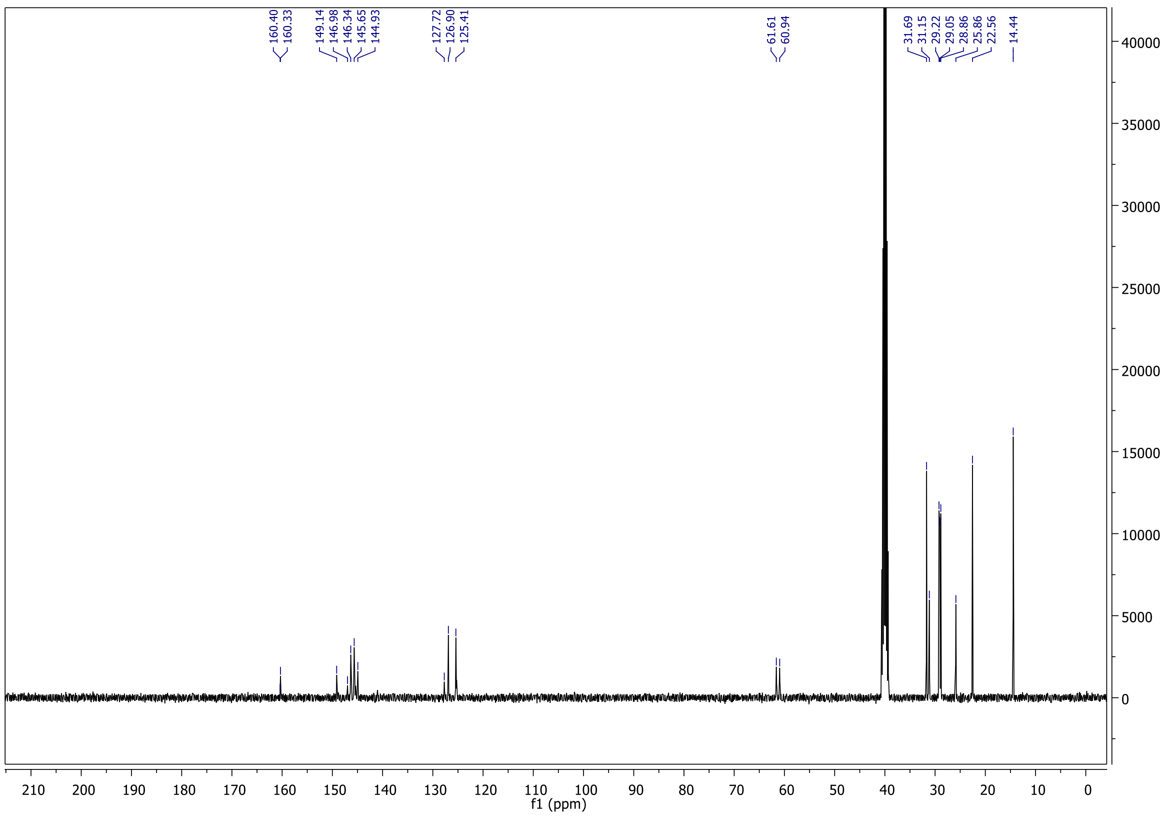

Figure S79: ^{13}C NMR of Compound **33**

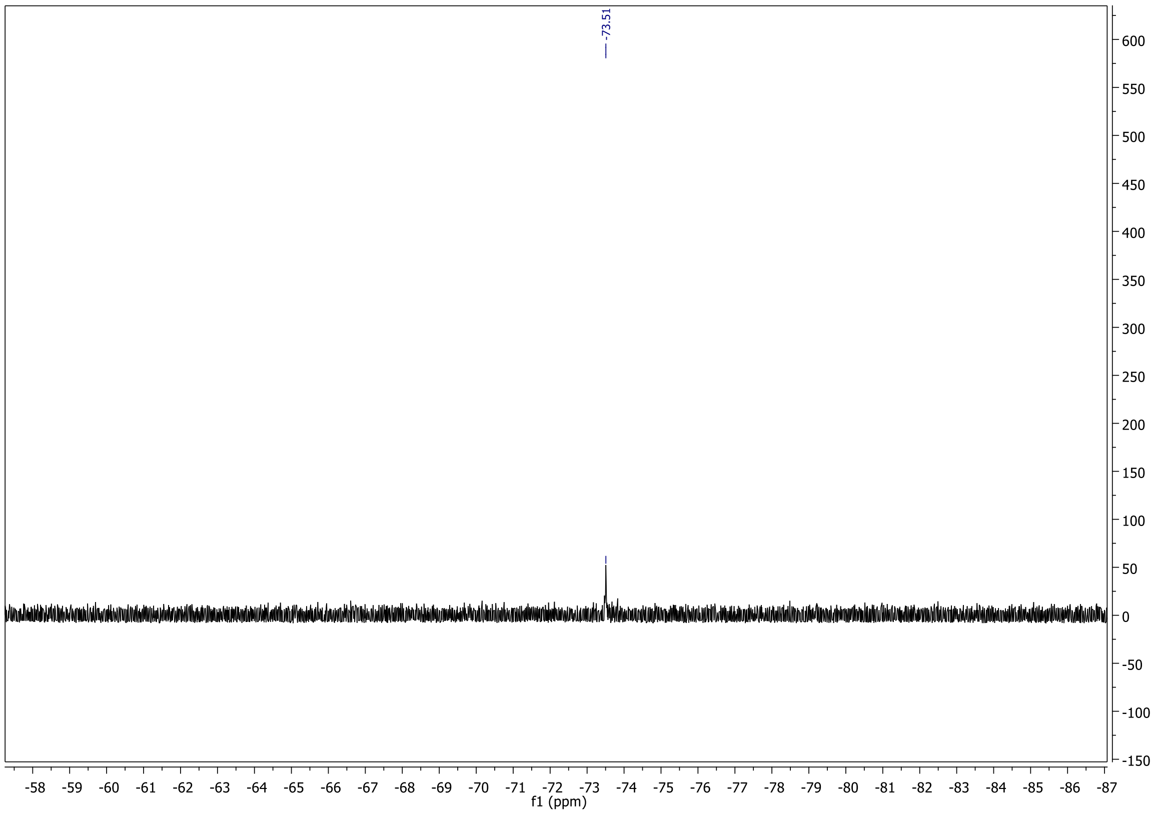

Figure S80: ^{19}F NMR of Compound **33**

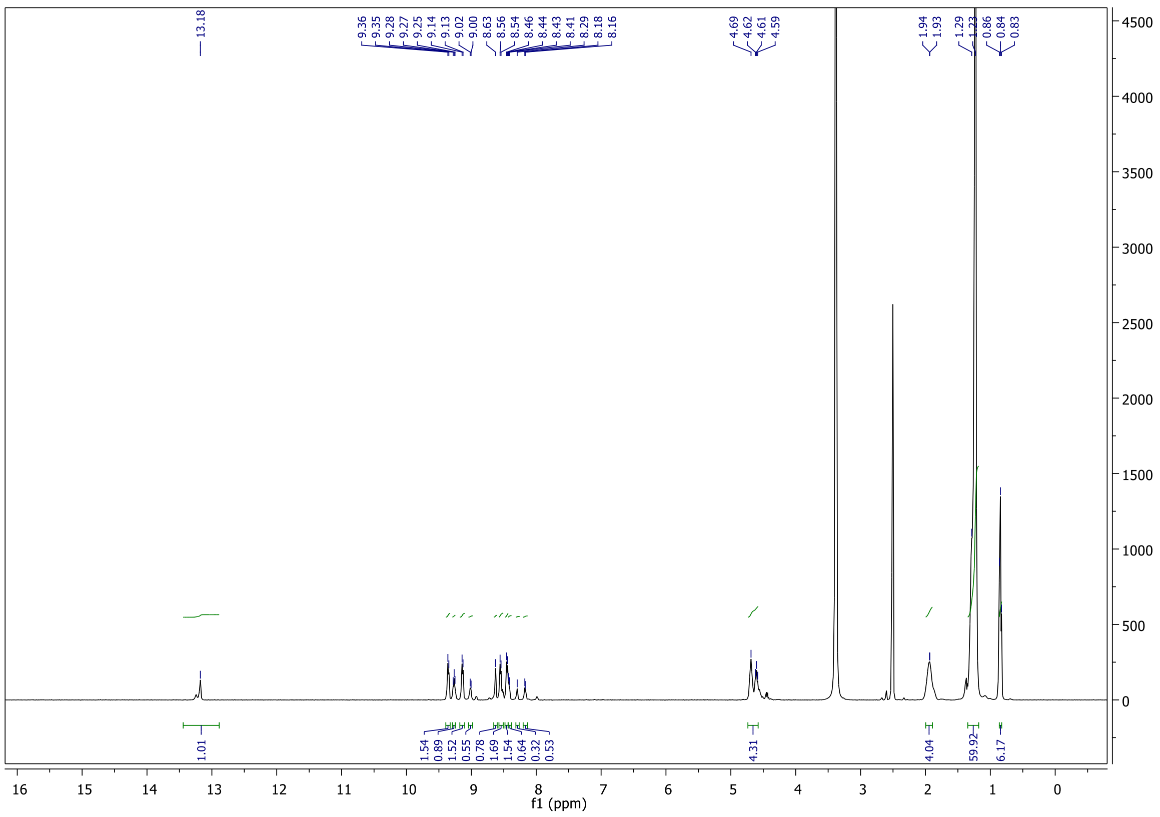

Figure S81: ^{1}H NMR of Compound **34**

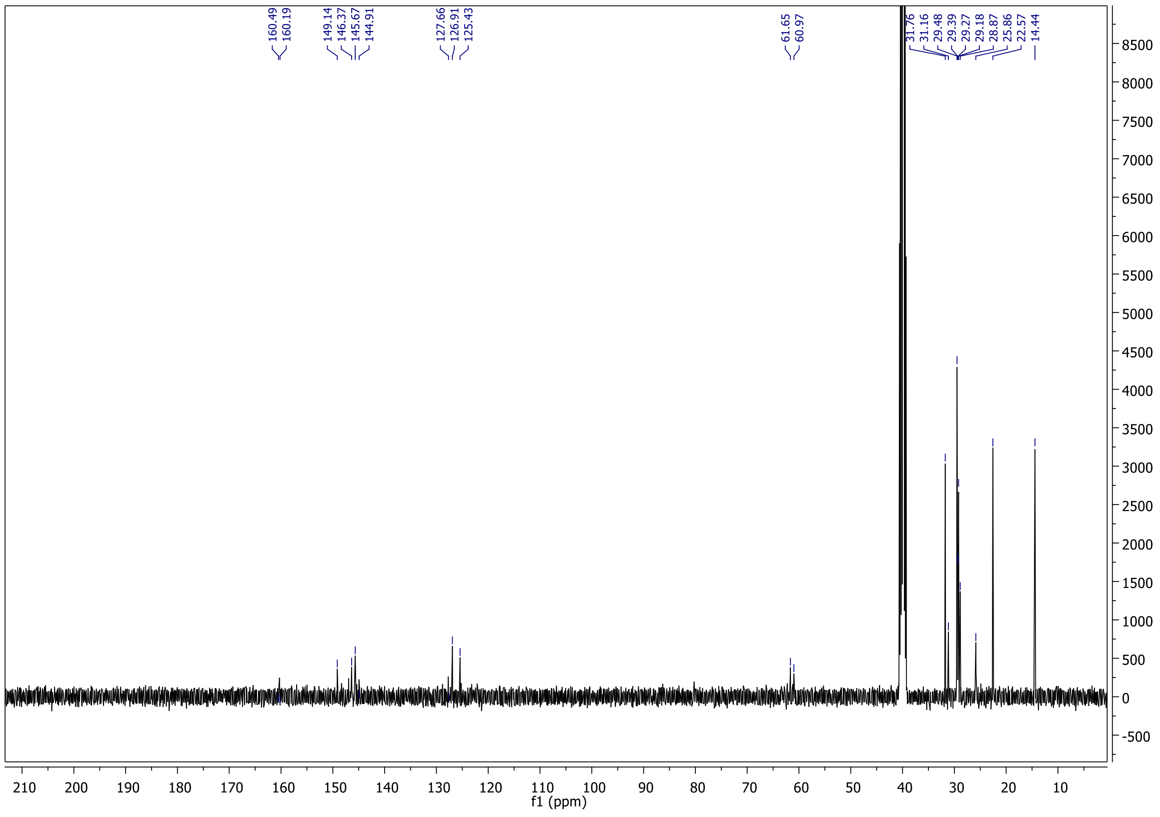

Figure S82: ^{13}C NMR of Compound **34**

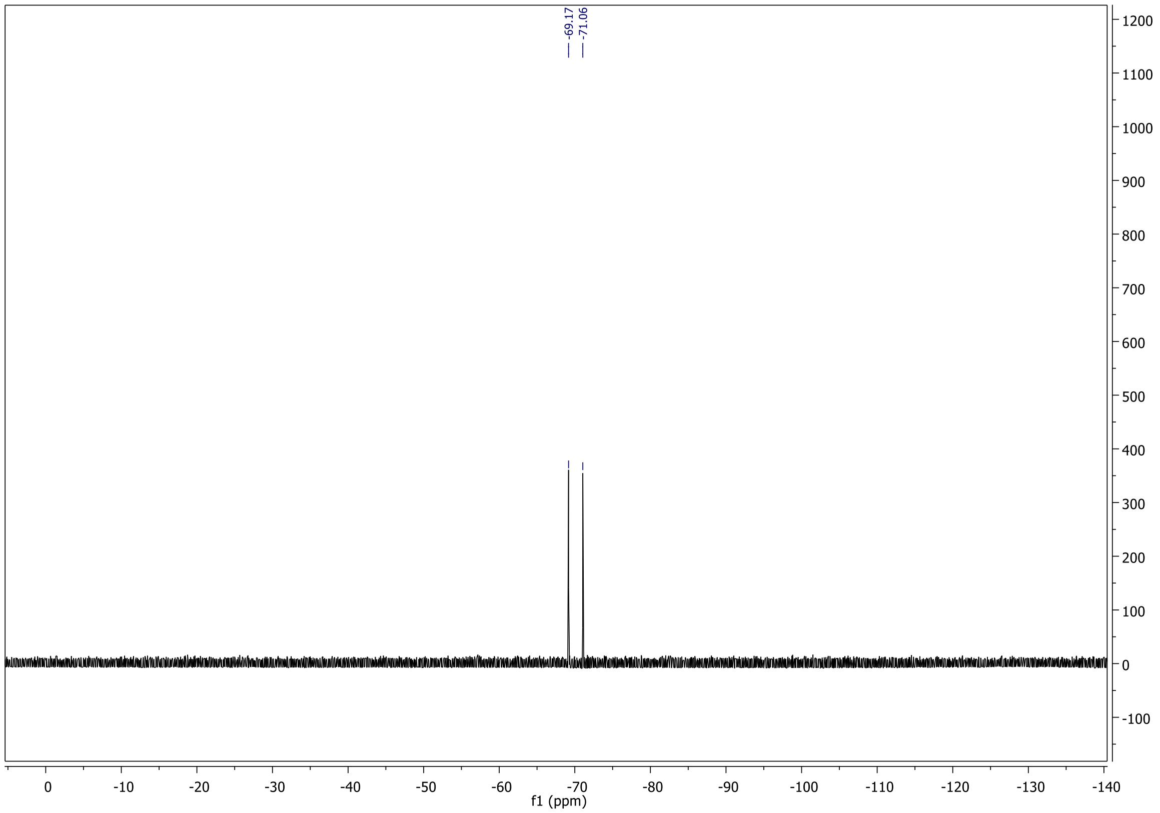

Figure S83: ^{19}F NMR of Compound **34**

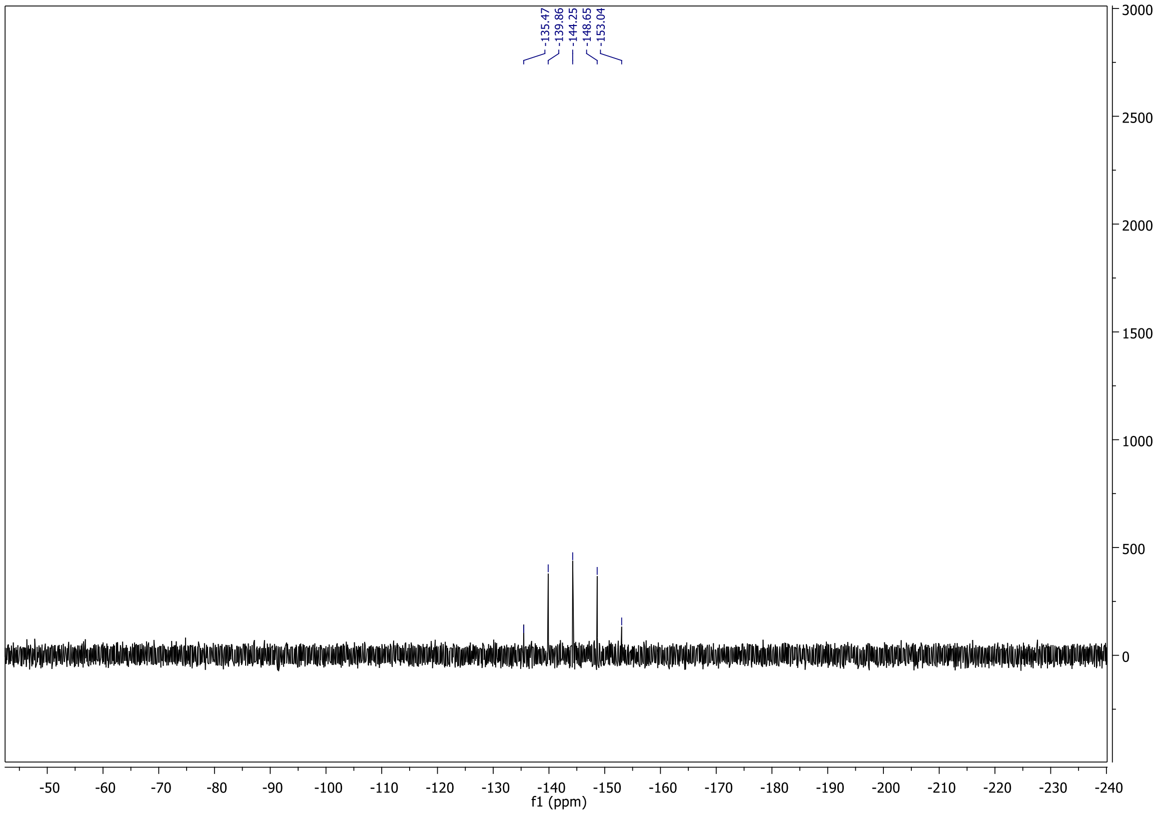

Figure S84: ^{31}P NMR of Compound **34**

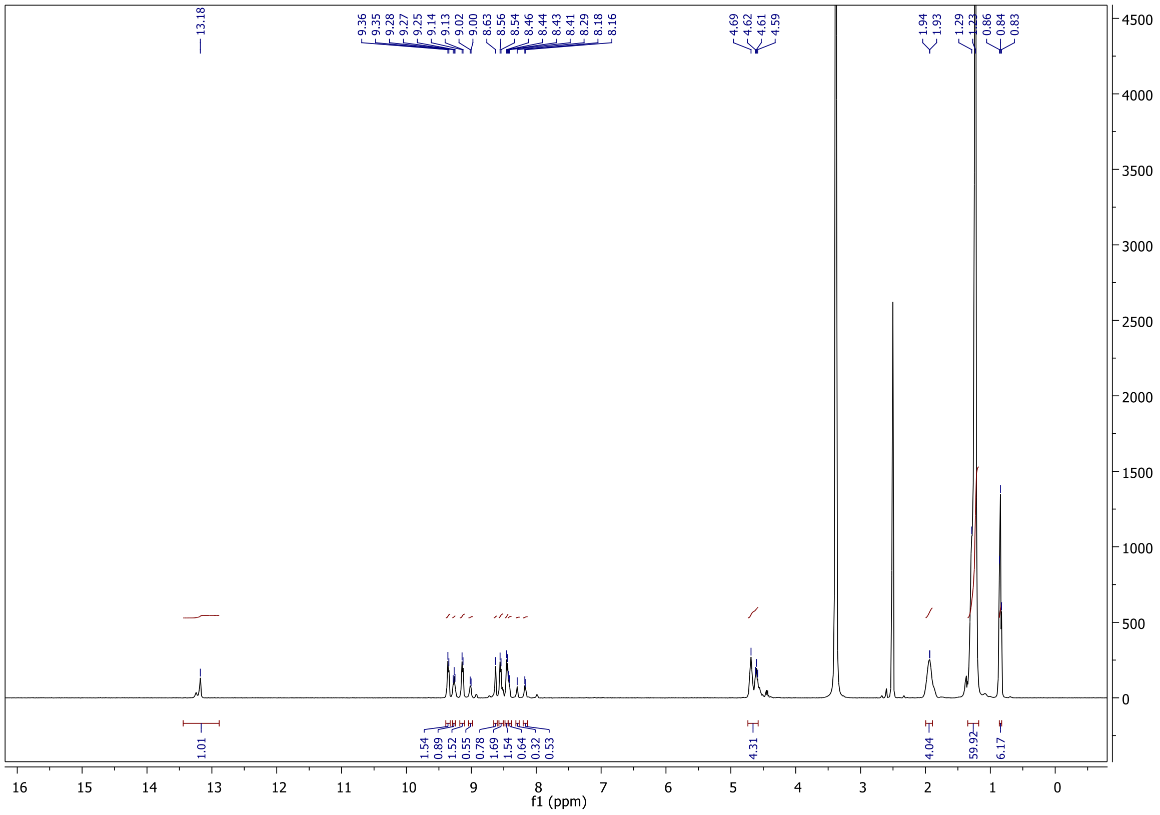

Figure S85: ^{1}H NMR of Compound **35**

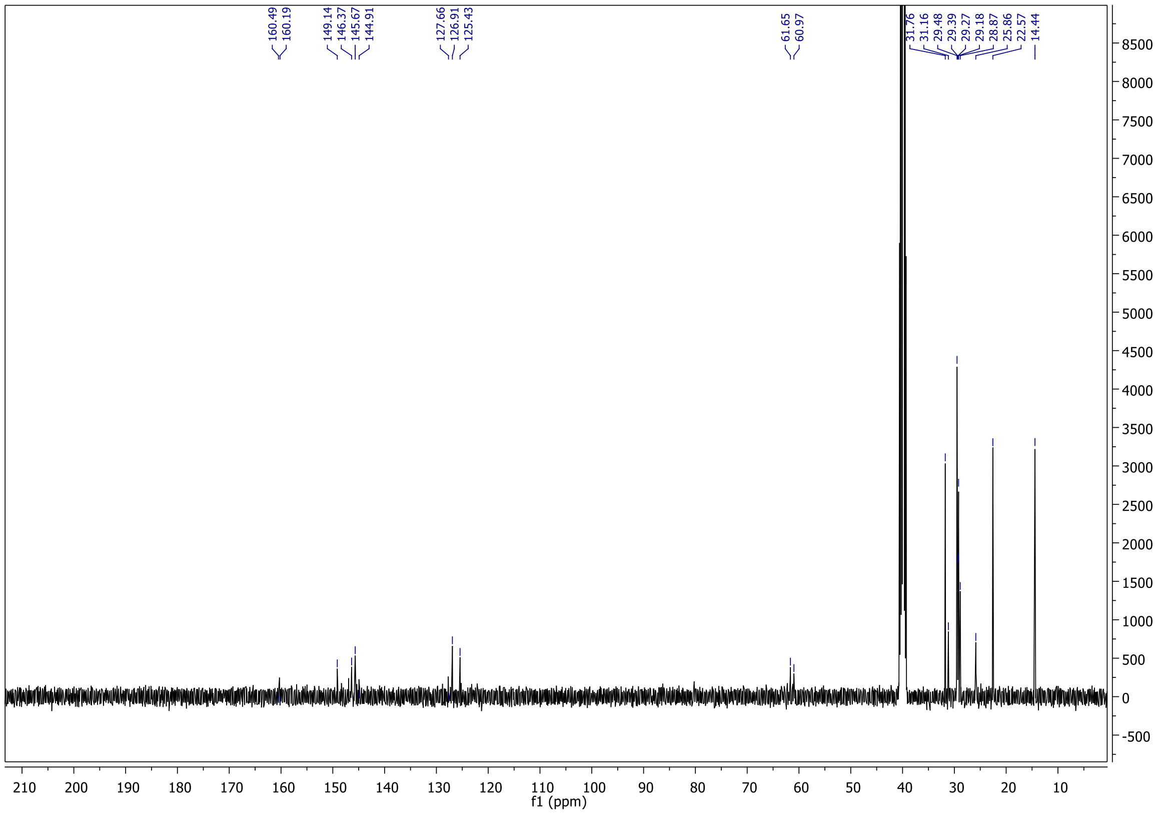

Figure S86: ^{13}C NMR of Compound **35**

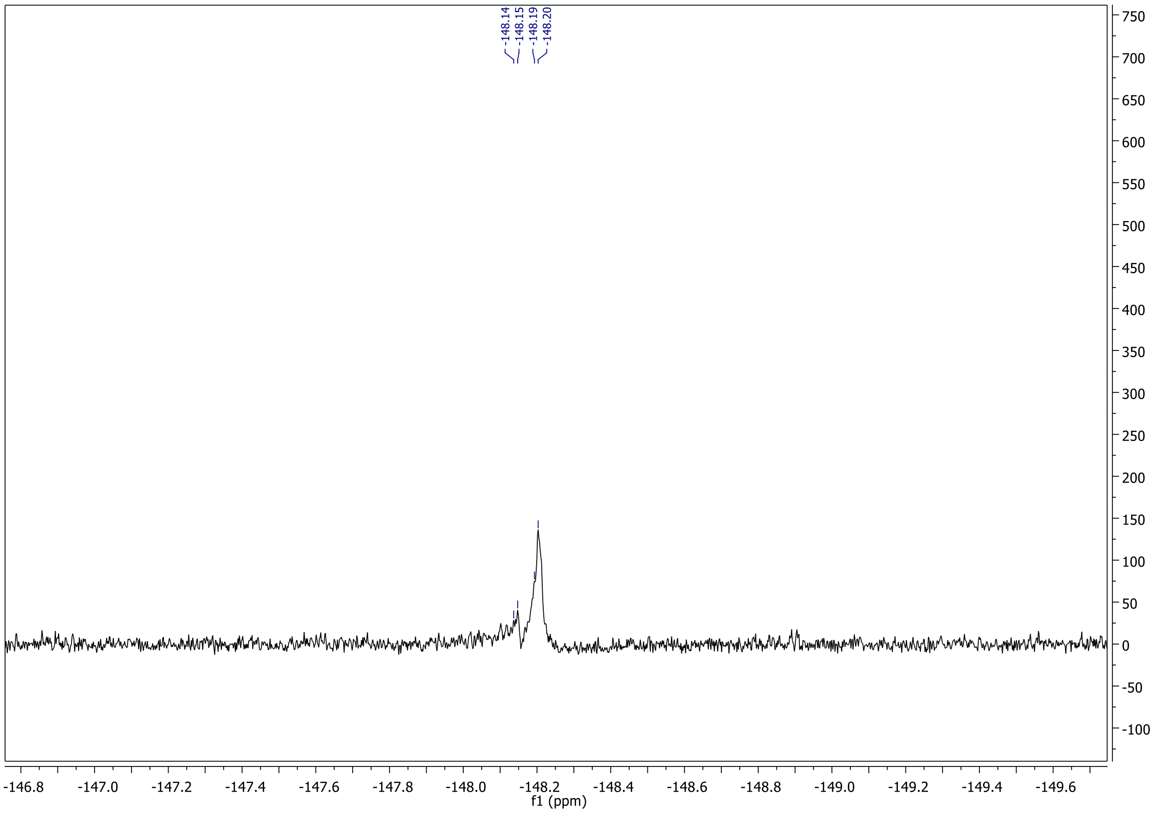

Figure S87: ^{19}F NMR of Compound **35**

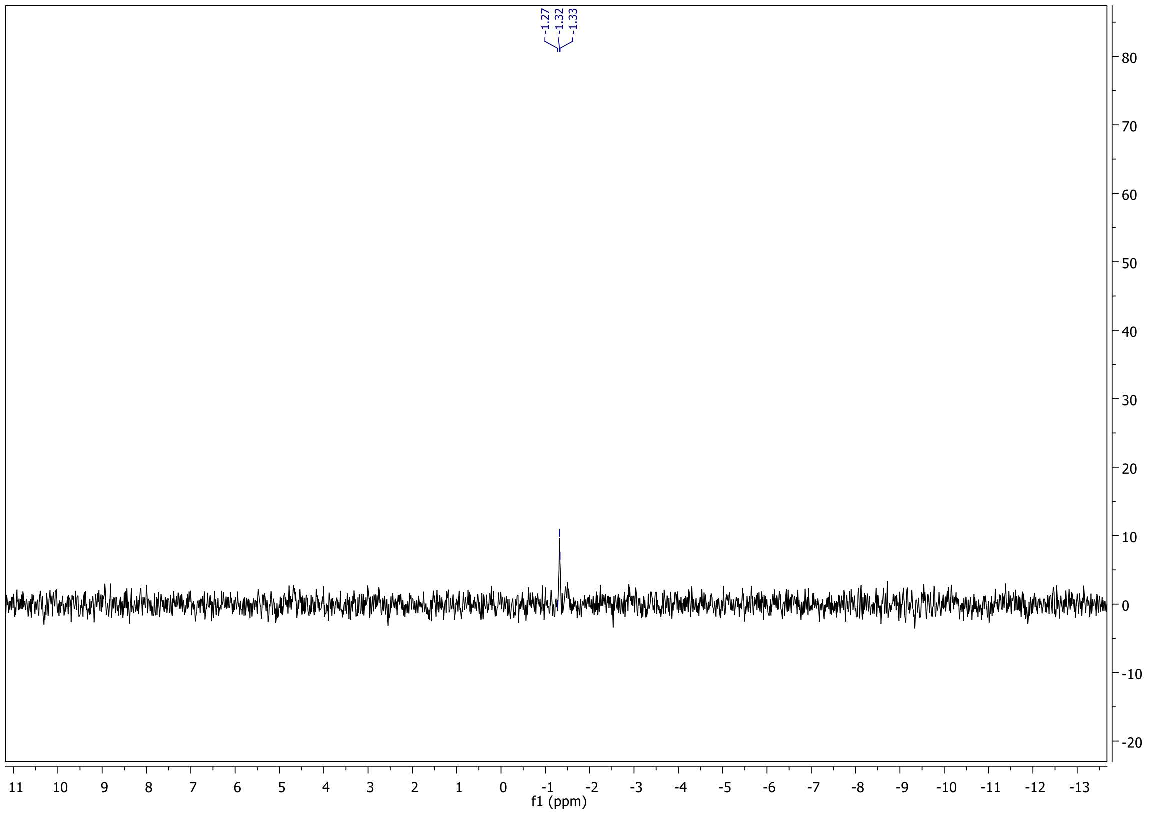

Figure S88: ^{11}B NMR of Compound **35**

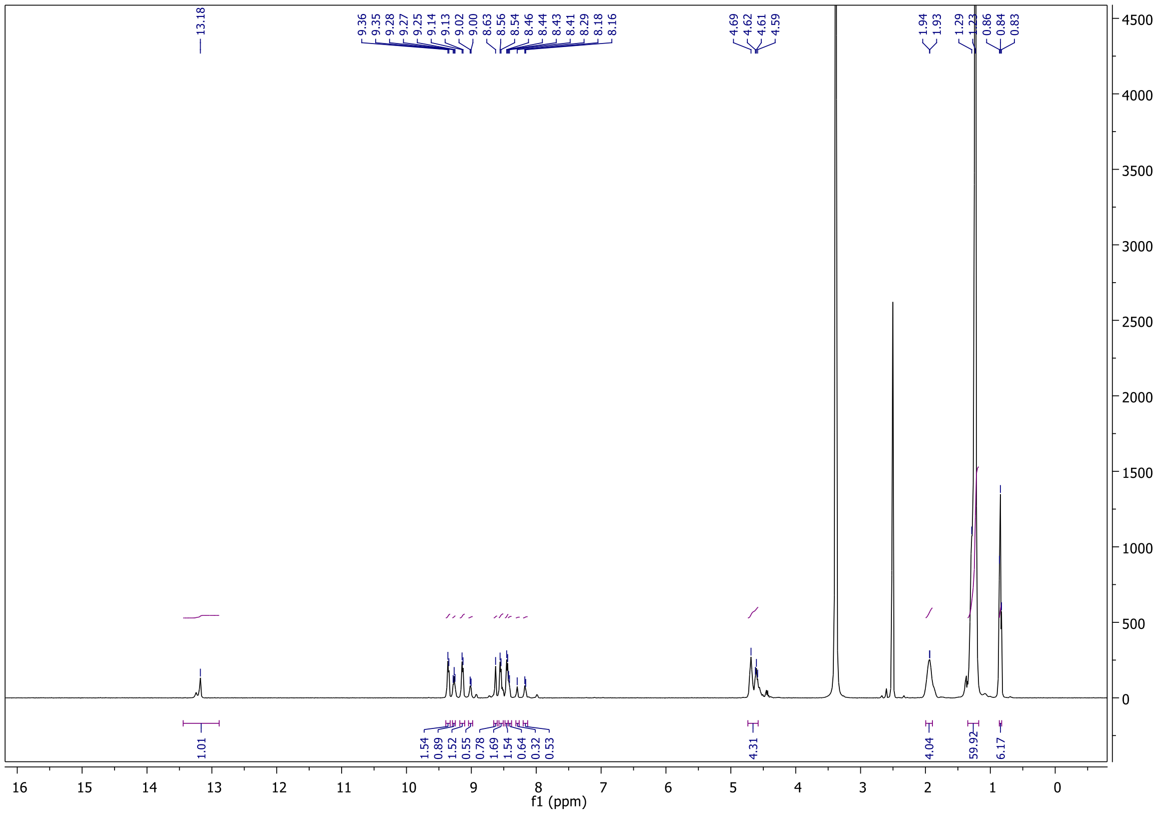

Figure S89: ^{1}H NMR of Compound **36**

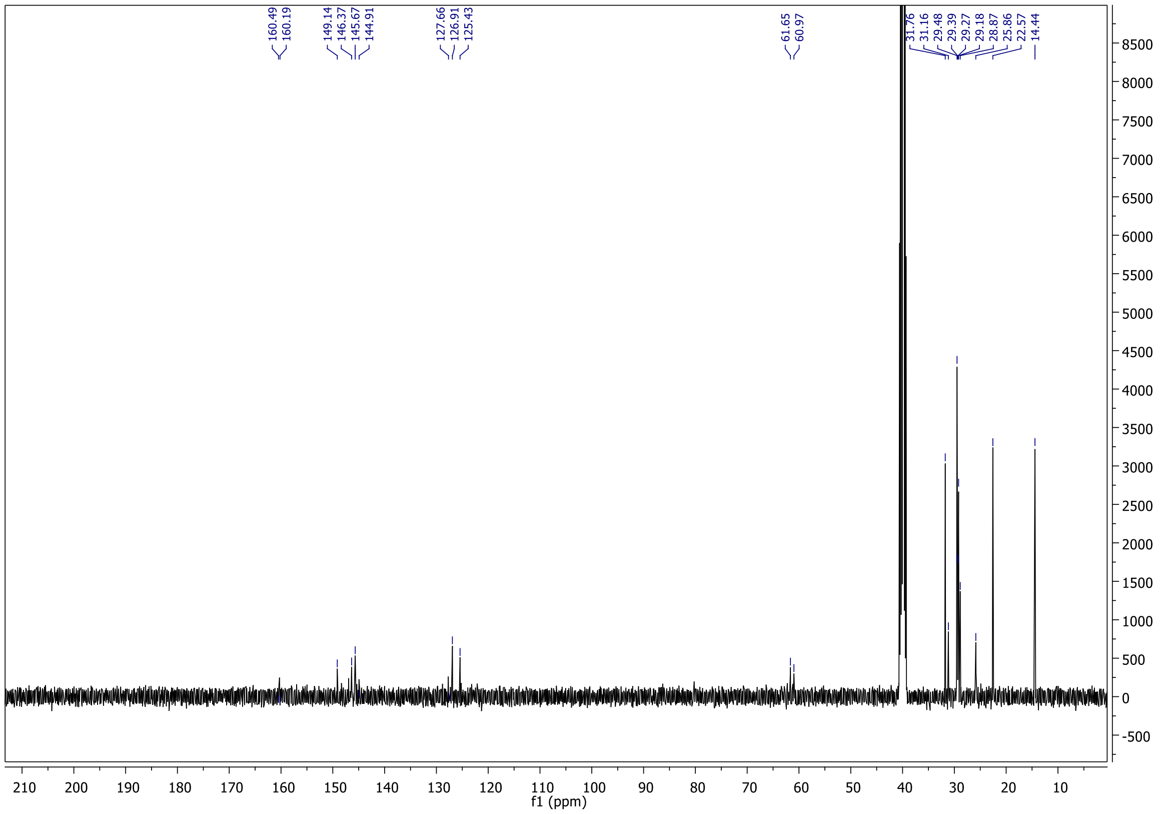

Figure S90: ^{13}C NMR of Compound **36**

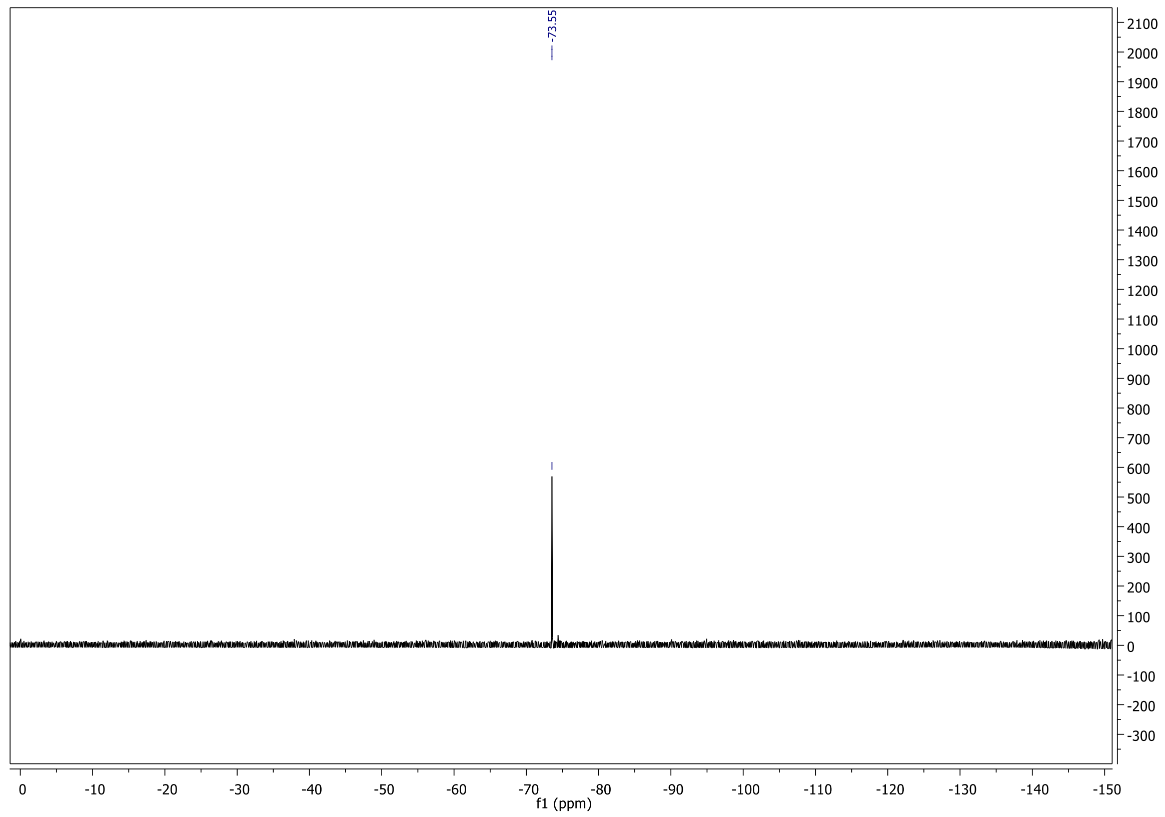

Figure S91: ^{19}F NMR of Compound **36**

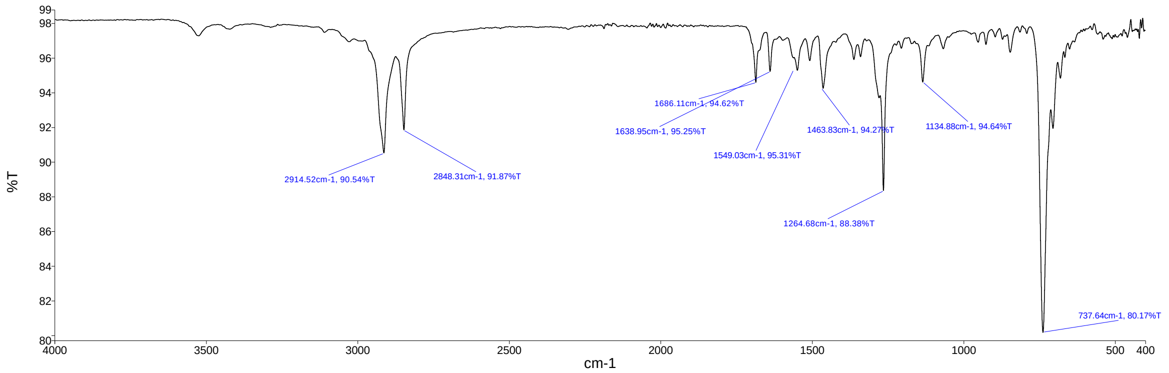

Figure S92: IR of Compound **9**

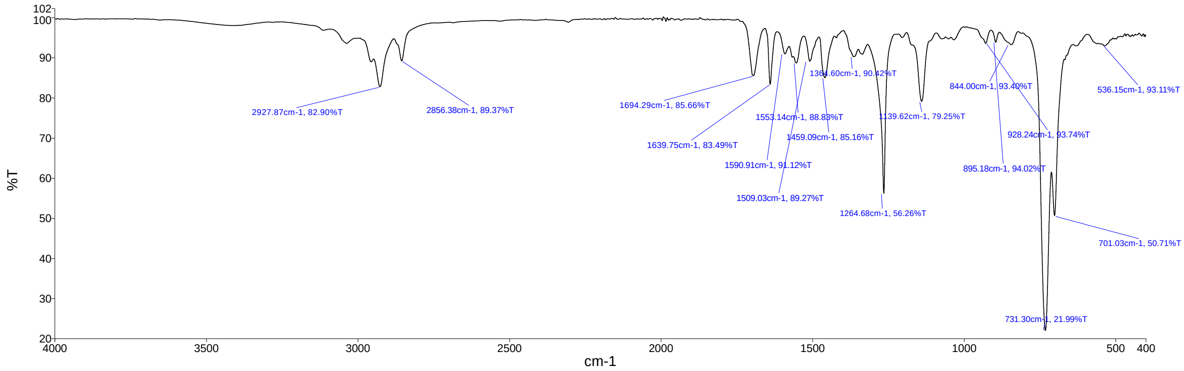

Figure S93: IR of Compound **10**

4. Thermograms data

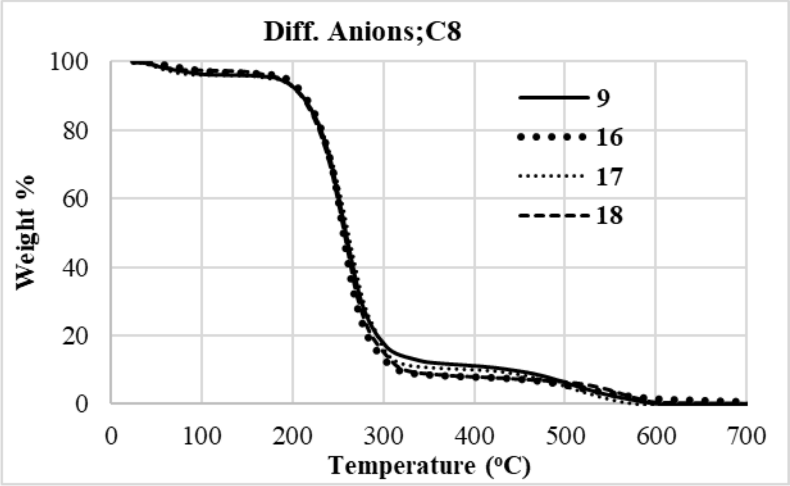

Figure S94: Thermograms of compounds **9** and **16-18**.

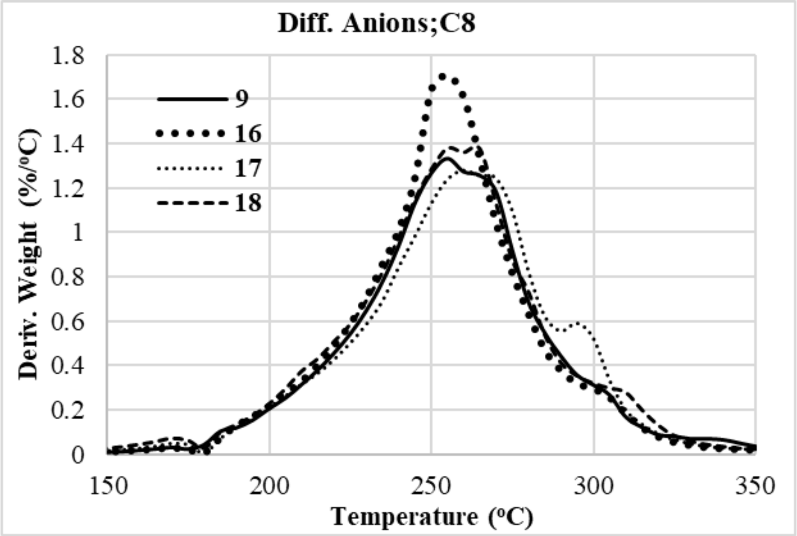

Figure S95: DTG curves of compounds **9** and **16-18**

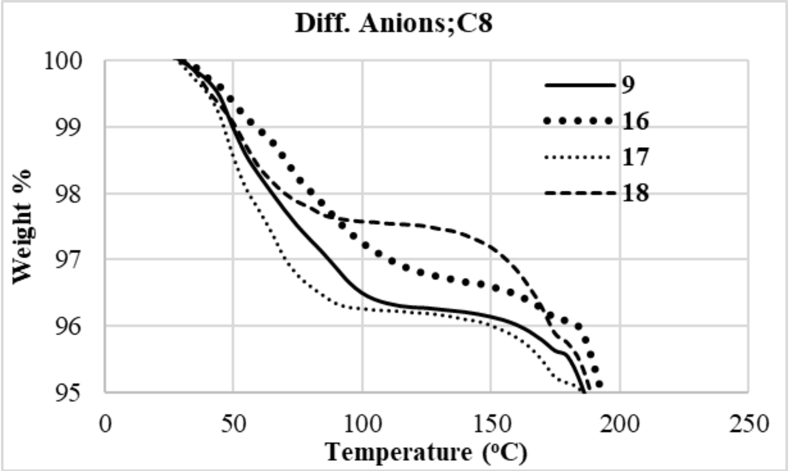

Figure S96: Initial temperature range in TGA of compounds **9** and **16-18**

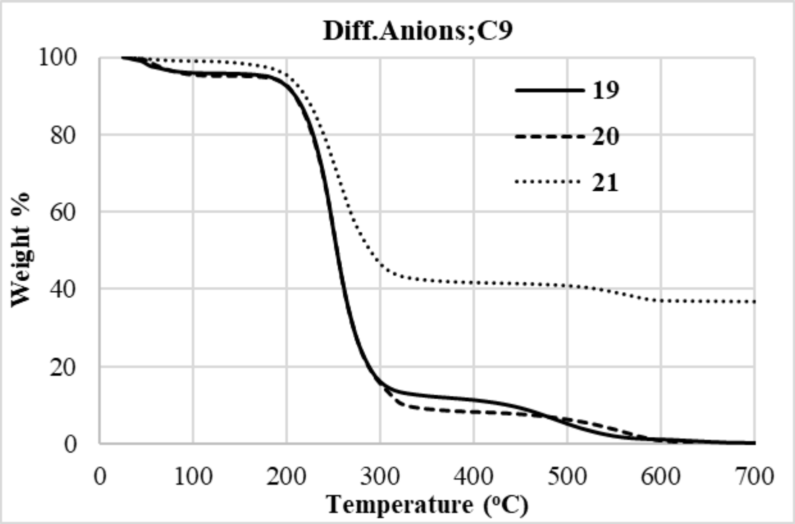

Figure S97: Thermograms of compounds **19-21**.

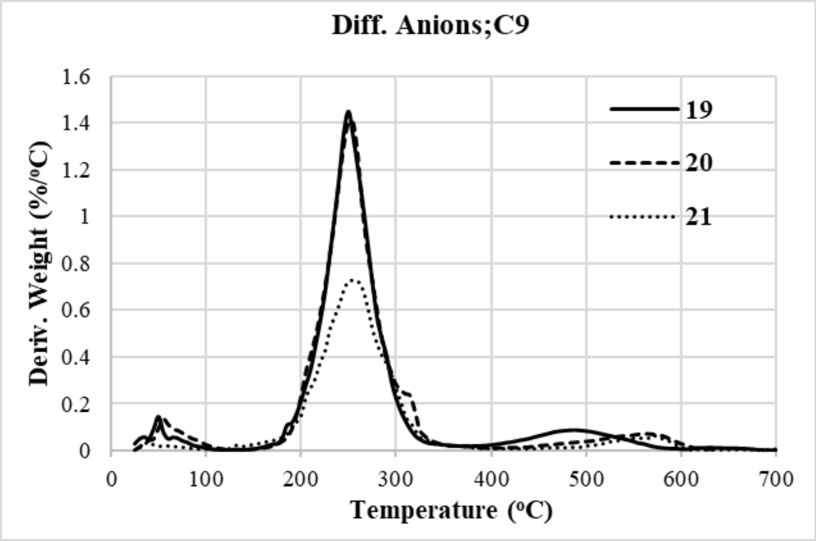

Figure S98: DTG curves compounds **19-21**

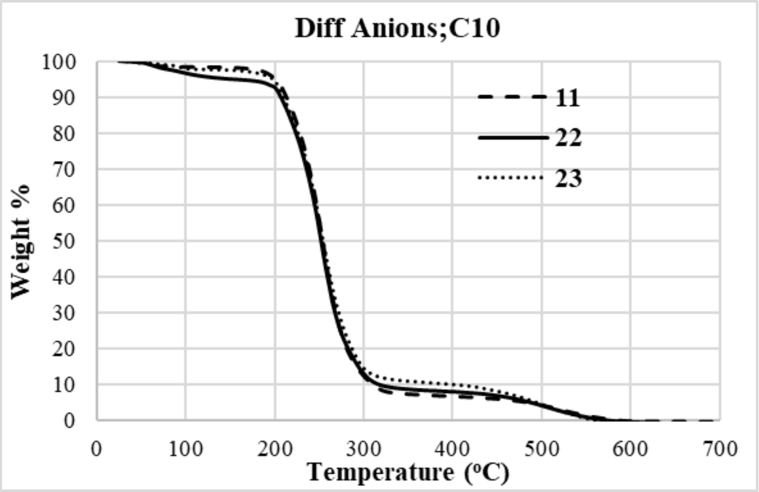

Figure S99: Thermograms of compounds **11** and **22-23**

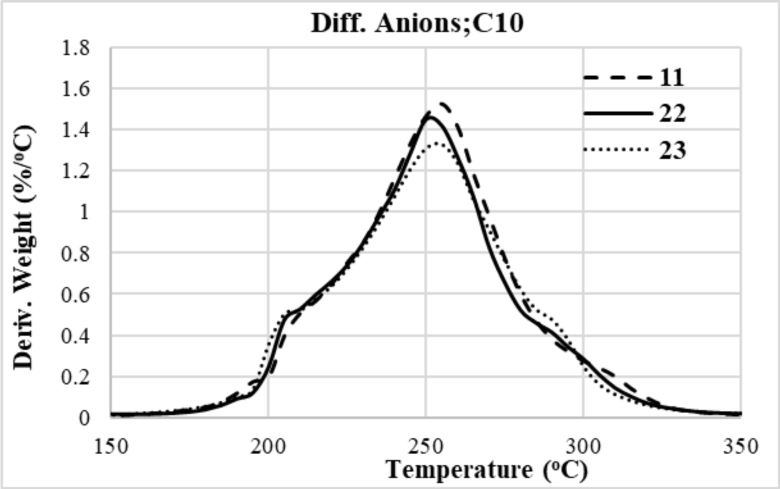

Figure S100: DTG curves compounds **11** and **22-23**

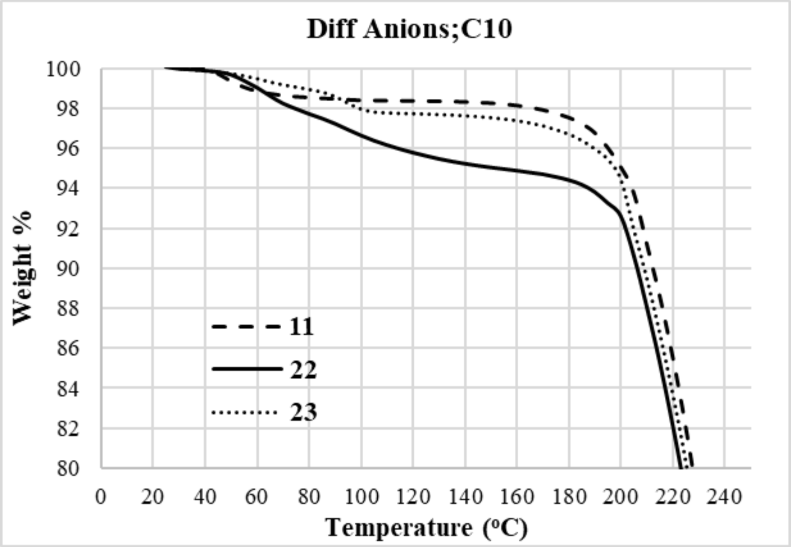

Figure S101: Initial temperature range in TGA of compounds **11** and **22-23**

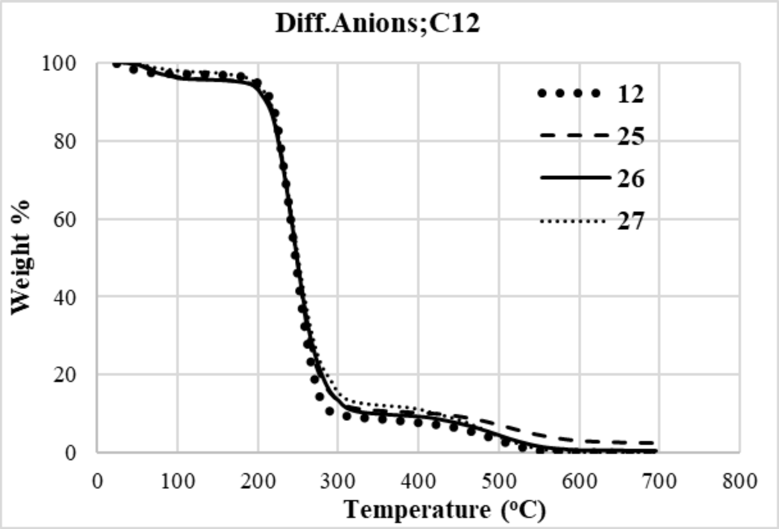

Figure S102: Thermograms of compounds **12** and **25-27**

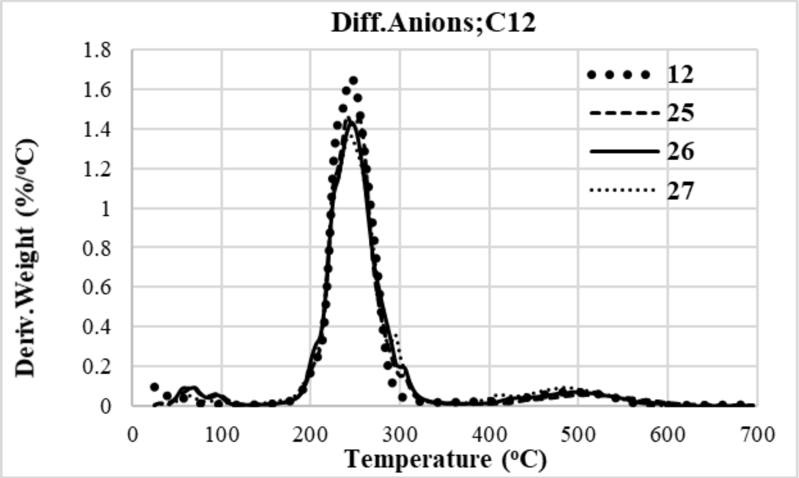

Figure S103: DTG curves compounds **12** and **25-27**

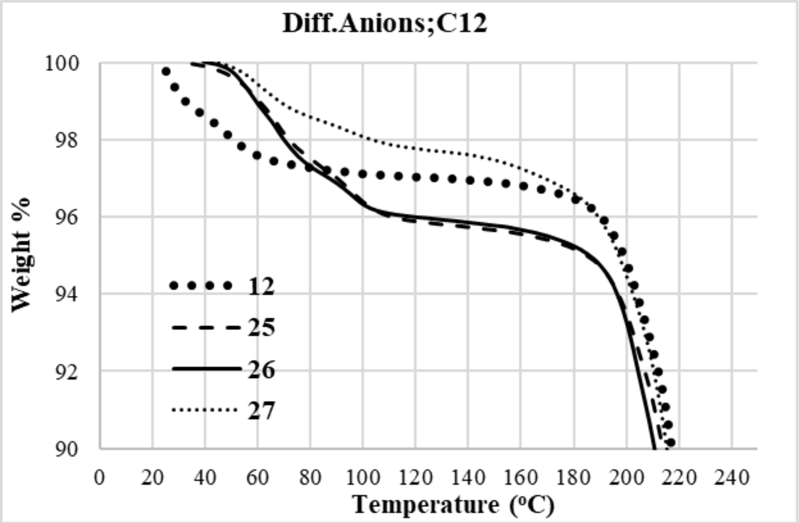

Figure S104: Initial temperature range in TGA of compounds **12** and **25-27**

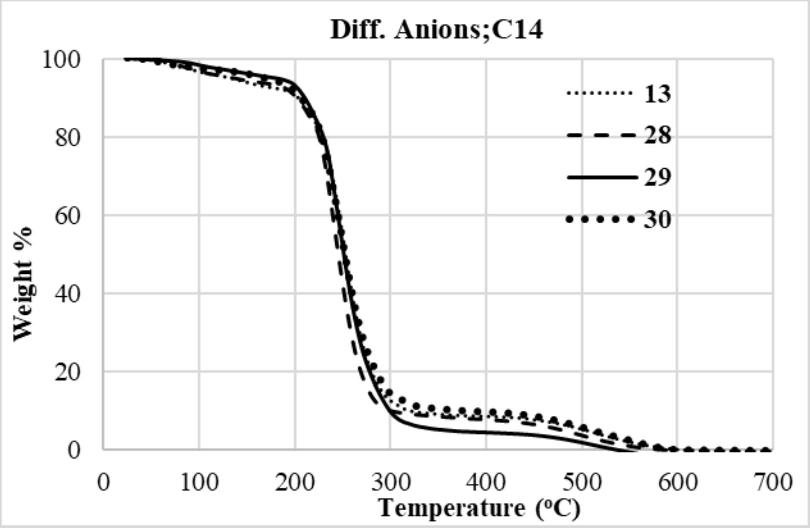

Figure S105: Thermograms of compounds **13** and **28-30**

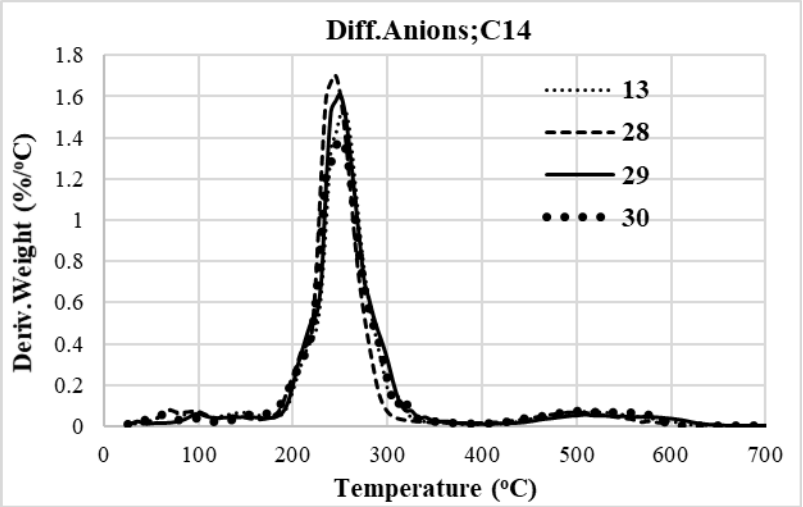

Figure S106: DTG curves compounds **13** and **28-30**

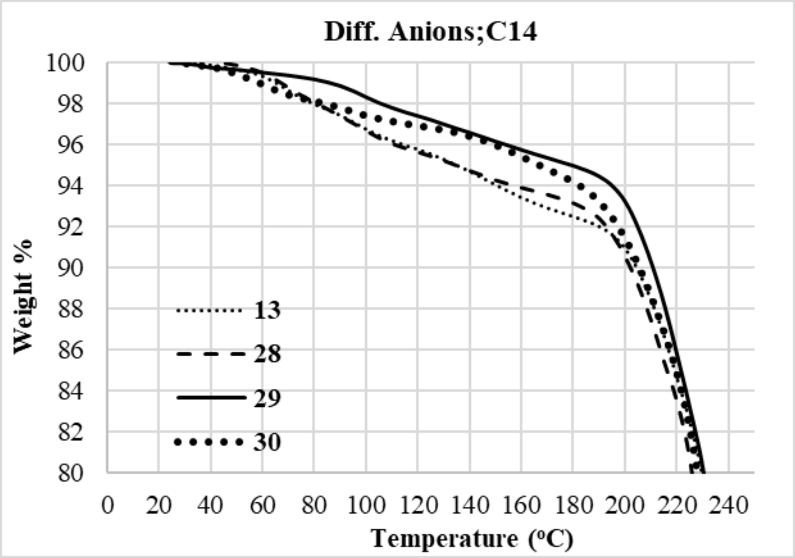

Figure S107: Initial temperature range in TGA of compounds **13** and **28-30**

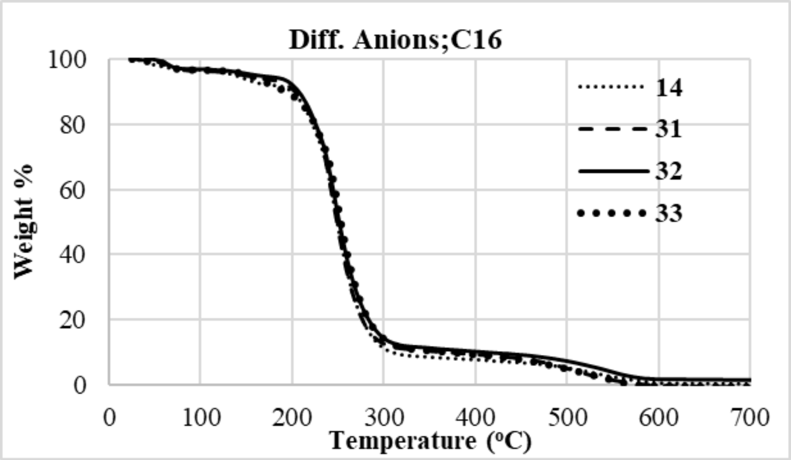

Figure S108: Thermograms of compounds **14** and **31-33**

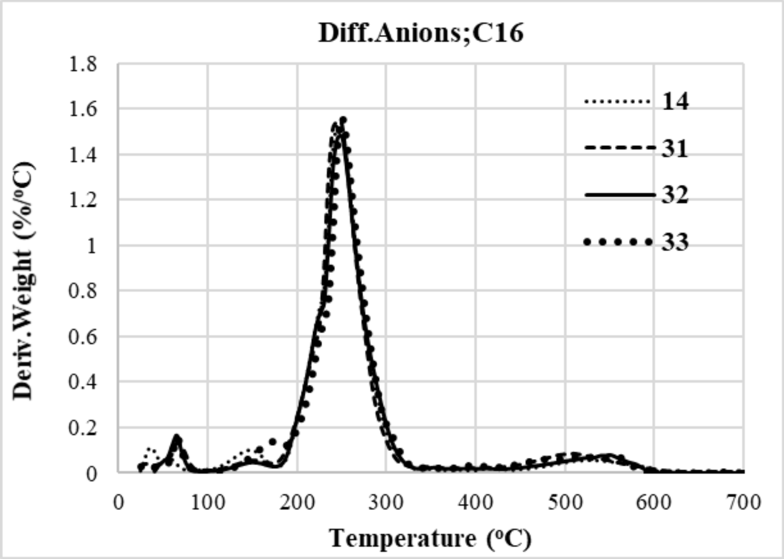

Figure S109: DTG curves compounds **14** and **31-33**

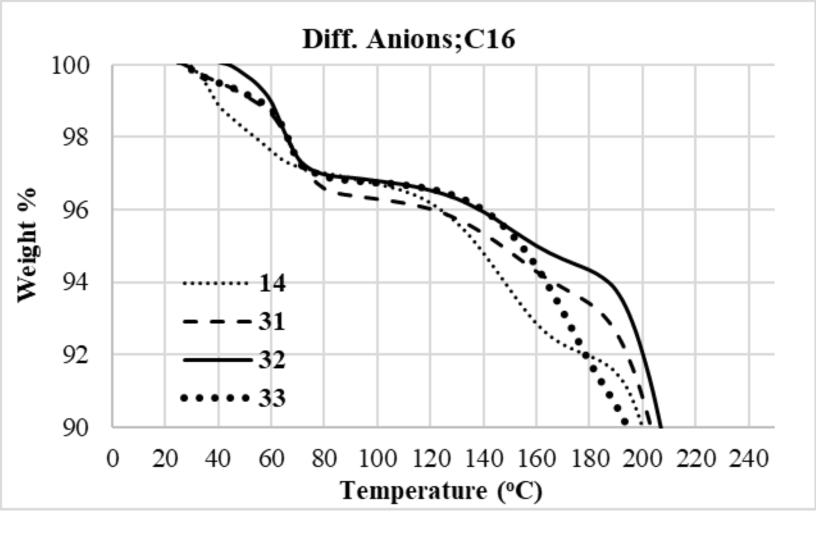

Figure S110: Initial temperature range in TGA of compounds **14** and **31-33**

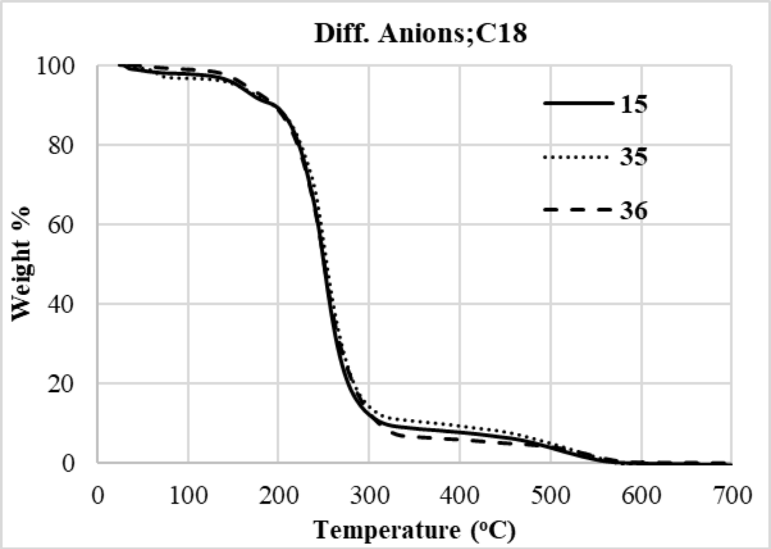

Figure S111: Thermograms of compounds **15** and **35-36**

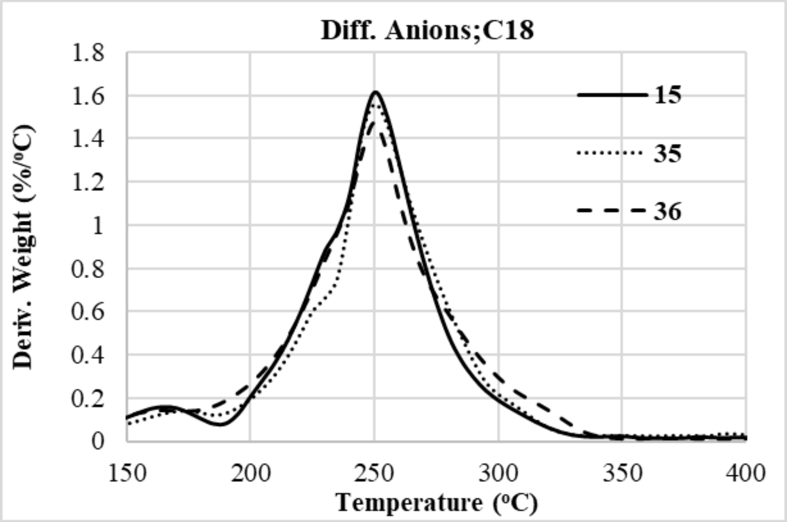

Figure S112: DTG curves compounds **15** and **35-36**

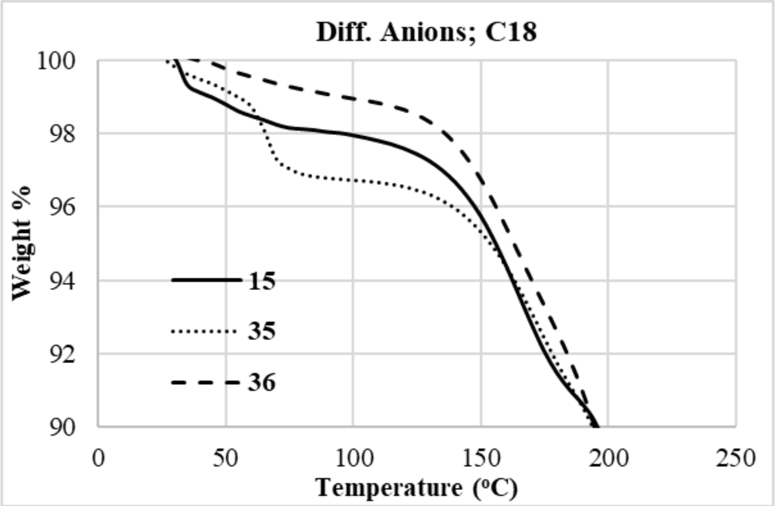

Figure S113: Initial temperature range in TGA of compounds **15** and **35-36**

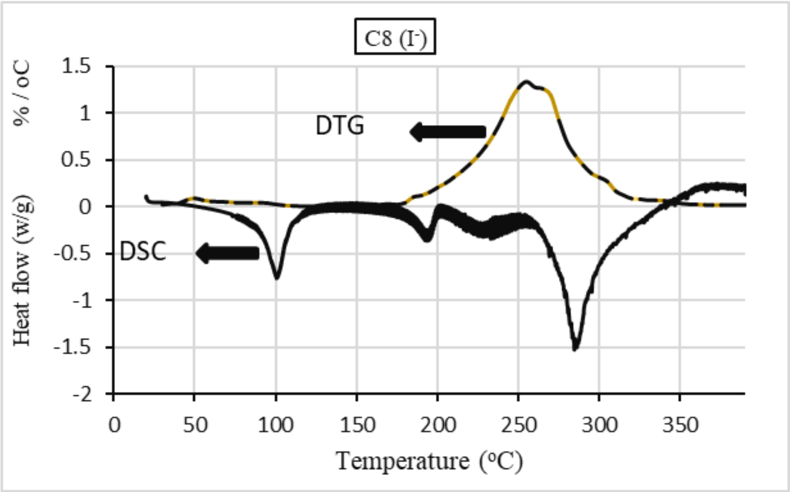

Figure S114: DTG/DSC curves of compound **9**

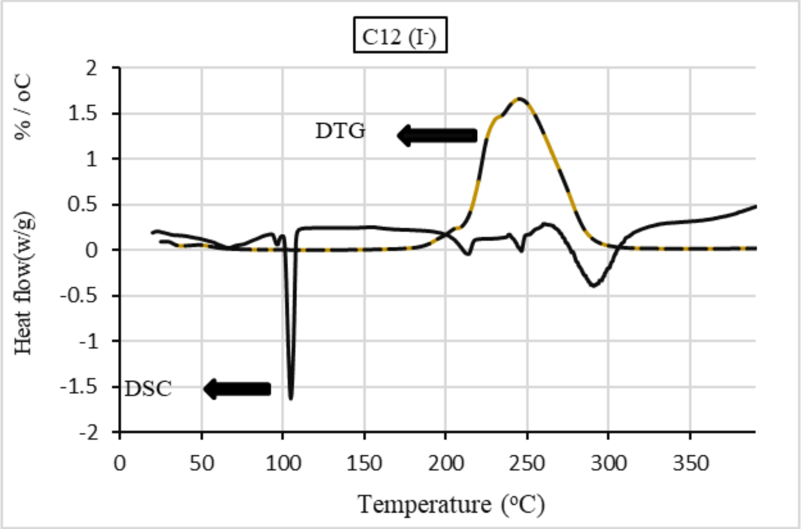

Figure S115: DTG/DSC curves of compound **12**

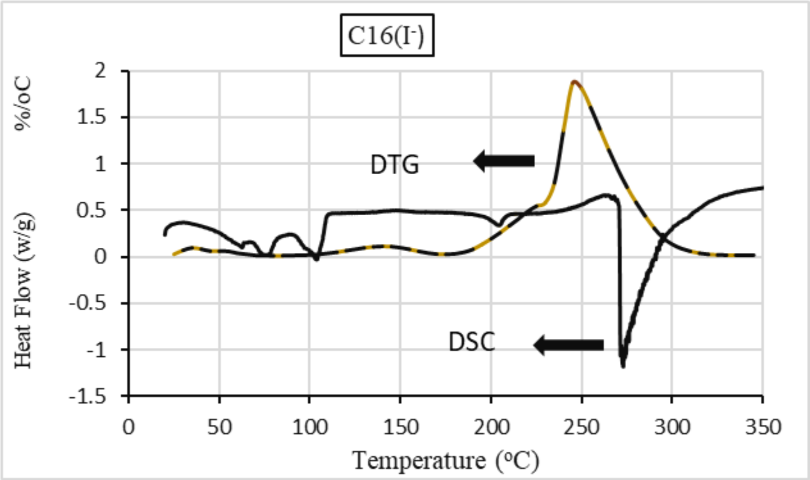

Figure S116: DTG/DSC curves of compound **14**

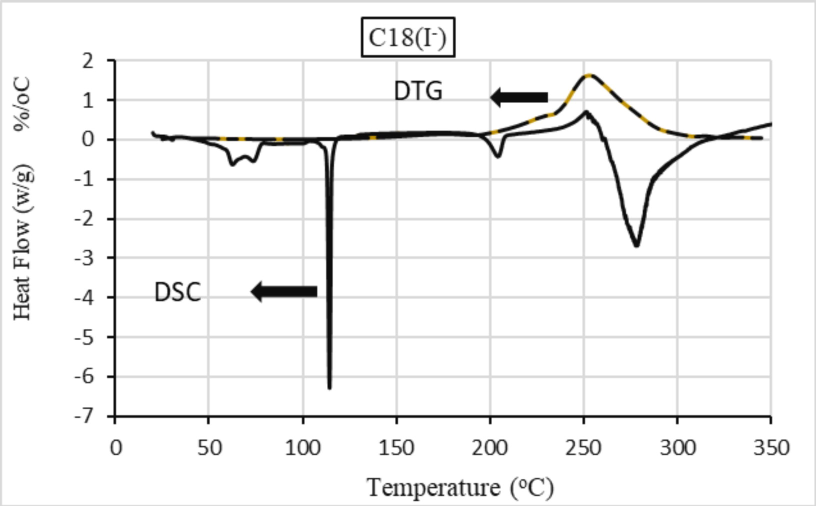

Figure S117: DTG/DSC curves of compound **15**

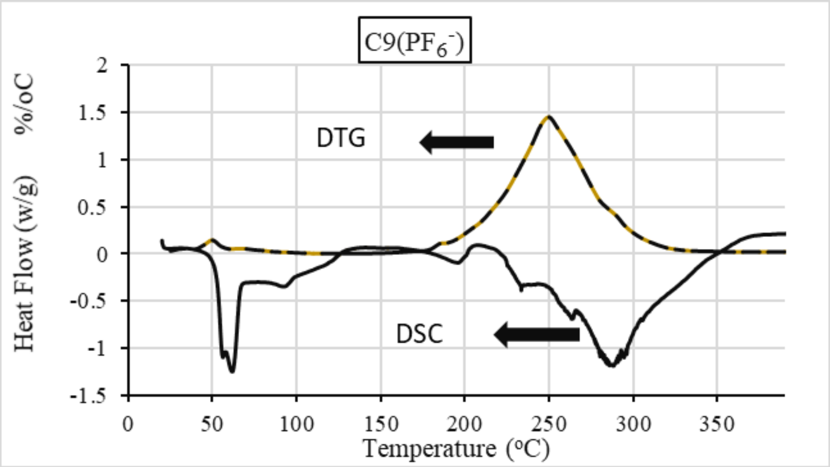

Figure S118: DTG/DSC curves of compound **15**

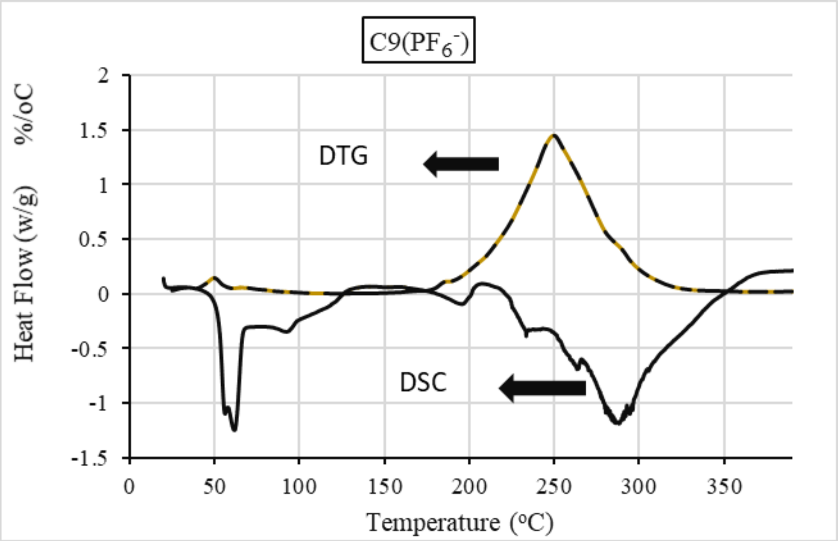

Figure S119: DTG/DSC curves of compound **19**

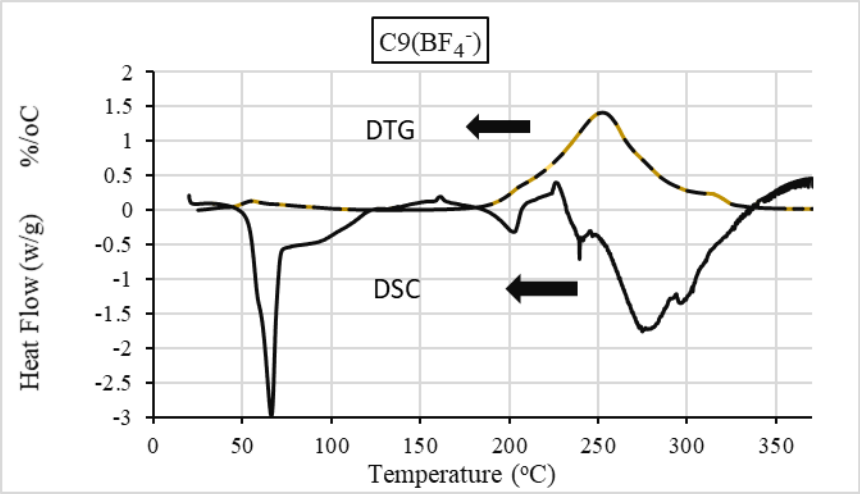

Figure S120: DTG/DSC curves of compound **20**

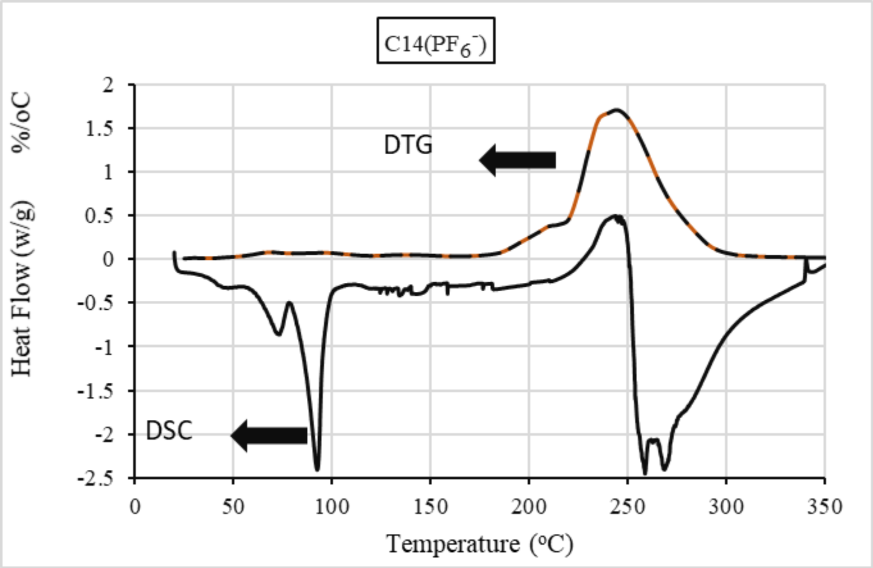

Figure S121: DTG/DSC curves of compound **28**

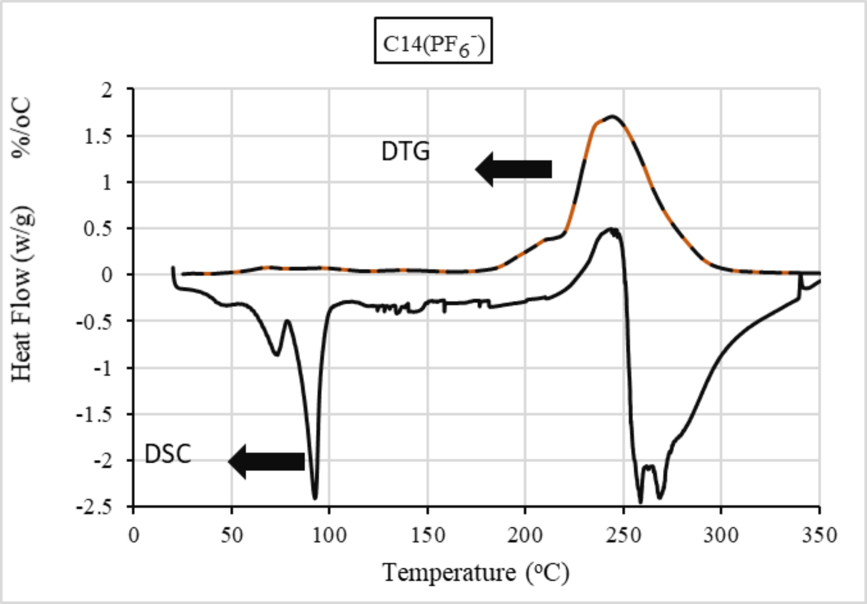

Figure S122: DTG/DSC curves of compound **29**
